# Alternative Performance Measures: A Structured Literature Review of Research in Academic and Professional Journals

**DOI:** 10.1007/s41471-022-00138-8

**Published:** 2022-09-06

**Authors:** Sascha B. Herr, Peter Lorson, Jochen Pilhofer

**Affiliations:** 1 Chair for Accounting, Management Control and Auditing, Department of Business Administration, University of Rostock, Ulmenstraße 69 (Thünen-Haus), 18057 Rostock, Germany; 2 Business School, University of Applied Sciences Saarbrücken (htw saar), Waldhausweg 14, 66123 Saarbrücken, Germany; 3 Professorship for Business Administration and Accounting, Business School, University of Applied Sciences Saarbrücken (htw saar), Waldhausweg 14, 66123 Saarbrücken, Germany

**Keywords:** Alternative performance measures, Non-GAAP (financial) measures, Pro forma earnings, Street earnings, Adjusted earnings, Structured literature review, M40, M41

## Abstract

For more than two decades, the reporting of so-called ‘alternative performance measures’ (APMs) has been a common phenomenon in external financial reporting. APMs are voluntarily disclosed and generally unaudited performance measures. Typically, APMs modify earnings measures calculated in accordance with generally accepted accounting principles (GAAP) by (subjectively) adjusting certain earnings components. In the academic literature, with the information motive on the one hand and the motive of (adversarial) investor influence on the other hand, two alternative explanations for the voluntary reporting of alternative performance measures are discussed, which are difficult, if not impossible, for external stakeholders to disentangle. Taking into account the recent developments in more than 250 published articles in the last decade, this paper critically reviews a wide range of literature from the United States (U.S.), Europe and, to a less extent, Australia/Asia. In particular, we analyse a comprehensive sample of more than 400 research papers published in academic and professional journals as well as other publications which are important in the academic discourse. The purpose of this paper is to identify relevant research gaps that provide starting points for future research. For this purpose, our methodological approach strictly follows structured literature review (SLR) methodology in order to minimise researcher idiosyncrasies. Thus, our SLR facilitates a decided derivation of research gaps based on a reliable and valid analytical framework which has been deductively derived from previous research.

**Supplementary Information:**

The online version of this article (10.1007/s41471-022-00138-8) contains supplementary material, which is available to authorised users.

## Introduction

In the U.S., the publication of APMs has already been a common phenomenon for more than two decades (Pizzo 2020). APMs, also referred to as ‘non-GAAP (financial) measures’ (e.g., Moscariello and Pizzo 2020; Marques 2010) or ‘pro forma earnings’^1^ (e.g., Bhattacharya et al. 2003; Hillebrandt and Sellhorn 2002),^2^ are financial performance measures voluntarily published by companies. Typically, APMs modify an earnings measure calculated in accordance with GAAP by excluding certain earnings components. Already in 1973, the *U.S. Securities and Exchange Commission* (SEC 1973) warned against possible investor confusion through the use of financial performance measures not regulated by law. Nevertheless, in subsequent years the frequency and scope of reporting APMs by companies increased significantly with the beginning of the 1990s, especially in the last two decades (e.g., Pizzo 2020; Baumker et al. 2014). In addition, APM reporting has historically been favoured by a relatively low level of regulation, resulting in significant degrees of freedom (Henry et al. 2020b).

Besides the capital markets in the U.S., European companies also joined the ‘pro forma canon’ of the capital markets from the beginning of the 2000s (e.g., Lorson and Schedler 2002; Küting and Heiden 2003). Already since then, the academic literature has been dealing with this phenomenon (e.g., Fields et al. 1998; Bradshaw and Sloan 2002). The main focus in the academic literature is on whether management uses APM reporting to provide external stakeholders with decision-useful information or to positively influence their perception of companies’ earnings performance. Taken together, early empirical evidence seems to suggest that APMs can be decision-useful for capital markets (e.g., Bhattacharya et al. 2003; Bradshaw and Sloan 2002). However, there also seem to be cases where investors are misled by APM reporting (e.g., Johnson et al. 2014; Dilla et al. 2013; Frederickson and Miller 2004). Such concerns about investors potentially being misled eventually led to regulatory action, beginning in the U.S. with *Regulation G* (Reg. G). Since 2003, listed U.S. companies have been required, inter alia, to reconcile disclosed APMs to the respective directly comparable GAAP measure (SEC 2002). Similar regulations exist in Europe since 2016 with the *Guidelines on Alternative Performance Measures* (ESMA guidelines) issued by the *European Securities and Markets Authority *(ESMA 2015).

Taking into account the recent developments during the last decade, in particular the regulatory initiative at the European level and the liberalisation of Reg. G rules in the U.S., the *research goal* of this paper is to find out how new regulations, definitions and guidance from standard setters may have opened up new perspectives for APM research. For this purpose, we conduct a SLR following the methodology proposed by Massaro et al. (2016). Our SLR builds essentially upon the findings of the previous literature review by Hitz (2010a). We draw our *motivation* from more than 250 research papers on APMs that have been published since 2010, including over 100 articles in academic journals. Our paper aims to critically review this research, covering a wide range of U.S., European and, to a less extent, Australian/Asian literature. In particular, we analyse a comprehensive sample of more than 400 research papers in order to contribute to the synthesis of previous findings and identify promising future research paths. In line with SLR methodology, we intend to answer the following three core research questions:

- *RQ1: How has the literature on APMs developed so far?*
- *RQ2: What is the focus and critique of APM literature?*
- *RQ3: What is the future for APM research?*

Our SLR is novel and *contributes* to the findings of previous literature reviews on APMs in at least four dimensions. *First,* to the best of our knowledge, we analyse the most comprehensive and recent sample of APM research papers, covering more than 400 articles.^3^ When analysing our sample, a particular focus is on reviewing the recent developments in more than 250 articles since the publication of the (traditional authorship) literature review by Hitz (2010a). Based on our comprehensive sample, we provide a thorough analysis of APM research and reveal how the literature on APMs has evolved in more than two decades since the late 1990s.

*Second*, in contrast to previous literature reviews (e.g., Arena et al. 2021; Catuogno and Arena 2020; Marques 2017; Hitz 2010a), our SLR provides for a broader coverage of APM literature. Since accounting research traditionally also takes place in other types of publications (Carmona 2006), we do not limit our sample only on articles published in renowned double-blind peer-reviewed academic journals. Instead, our SLR covers a wide range of different literature on APMs. Undisputedly, peer-reviewed academic journals are the most important medium for scientific publications in accounting research (Fülbier and Weller 2011; Raffournier and Schatt 2010; Easterby-Smith et al. 2012). However, according to the previous literature non-peer-reviewed publications are also of crucial importance for academic discourse (e.g., Carmona 2006; Fülbier and Weller 2011, 2008; Wagenhofer 2006). This is particularly true for (applied) research published in professional journals (Fülbier and Weller 2011; Fülbier and Gassen 2011), which allows to draw attention to undesirable developments in legislation and jurisdiction at an early stage and even prospectively due to shorter publication processes (Kußmaul et al. 2017; Küting et al. 2013). Like professional journals, monographs, contributions to collective works, commentaries, jurisdictional sources and textbooks can also be of crucial importance for the academic discourse (Fülbier and Weller 2011; Wagenhofer 2006; Küpper and Mattessich 2005; Schäffer et al. 2006). For these reasons, we also take into account non-peer-reviewed literature such as articles in professional journals, monographs, contributions to collective works and working/discussion/conference papers. However, due to language limitations, we only include articles written in English or German. Thereby, our SLR opens up a new perspective to the international readership by providing insights into German-language APM research. In order to counterbalance the tendencies that may arise from the incorporation of articles written in German and the inclusion of research articles not published in academic journals, we provide scenario analyses when presenting the results of our SLR (see Table 1). In these scenario analyses, we clearly highlight how the findings would change if the sample did not include any articles written in German (e.g., regarding the research location) or if the sample did not include other research than articles published in academic journals (e.g., regarding the research method).

**Table 1:** Analytical framework and results of analysis of articles on APMs

					Scenario analysis (△)	
**Categories**	Subcategories	K‑alpha	**Results (total sample)**		International articles only	Academic articles only
**A. Bibliographic data**	A1. Articles		**410**		*358*	*228*
	A2. Authors	1.0000	**628**		*564*	*391*
	A3. Institutions	1.0000	**353**		*315*	*237*
	A4. Publication media	1.0000	**135**		*121*	*87*
	A5. Years	1.0000	**1998–2021**		*1998–2021*	*1998–2021*
**B. Research focus**	B1. Cross-sectional and time series analyses of APMs	0.7733	**48**	**11.7%**	*−* *3.3%*	*−* *3.4%*
	B2. Motives for the reporting of APMs		**158**	**38.5%**	*+* *5.3%*	*+* *15.0%*
	B3. Regulation of APM reporting		**46**	**11.2%**	*−* *0.0%*	*+* *2.8%*
	B4. No mention of a specific research focus		**135**	**32.9%**	*−* *1.9%*	*−* *15.8%*
	B5. Other		**23**	**5.6%**	*−* *0.0%*	*+* *1.4%*
**C. Research method**	C1. Empirical—archival (database or archive)	0.8339	**226**	**55.1%**	*+* *3.0%*	*+* *14.6%*
	C2. Empirical—descriptive		**10**	**2.5%**	*−* *1.3%*	*−* *0.7%*
	C3. Empirical—experimental		**14**	**3.4%**	*+* *0.5%*	*+* *1.8%*
	C4. Empirical—case study		**17**	**4.2%**	*−* *1.6%*	*−* *1.5%*
	C5. Empirical—survey		**4**	**1.0%**	*−* *0.4%*	*−* *0.5%*
	C6. Non-empirical—analytical		**1**	**0.2%**	*+* *0.0%*	*+* *0.2%*
	C7. Non-empirical—theory		**83**	**20.2%**	*−* *1.5%*	*−* *9.3%*
	C8. Non-empirical—opinion		**50**	**12.2%**	*+* *1.2%*	*−* *4.3%*
	C9. Method not assignable		**5**	**1.2%**	*+* *0.2%*	*−* *0.3%*
**D. Location**	D1. North America	0.9705	**247**	**60.2%**	*+* *8.8%*	*+* *6.4%*
	D2. Australasia		**41**	**10.0%**	*+* *1.5%*	*+* *5.4%*
	D3. United Kingdom		**14**	**3.4%**	*+* *0.2%*	*−* *0.8%*
	D4. Europe		**91**	**22.2%**	*−* *11.0%*	*−* *12.1%*
	D5. Other		**17**	**4.2%**	*+* *0.6%*	*+* *1.1%*
**E. Accounting regimes**	E1. IFRS	0.8637	**61**	**14.9%**	*−* *5.9%*	*−* *5.7%*
	E2. U.S. GAAP		**206**	**50.2%**	*+* *7.3%*	*+* *6.8%*
	E3. Various accounting regimes		**48**	**11.7%**	*−* *2.5%*	*−* *2.1%*
	E4. No specification		**70**	**17.1%**	*+* *0.5%*	*−* *1.3%*
	E5. Other		**25**	**6.1%**	*+* *0.6%*	*+* *2.2%*
**F. Types of APMs**	F1. Voluntary APM disclosures by companies	0.7937	**121**	**29.5%**	*+* *4.8%*	*+* *7.3%*
	F2. APM disclosures in mandatory financial reports		**102**	**24.9%**	*−* *9.2%*	*−* *7.8%*
	F3. Analyst-reported street earnings		**54**	**13.2%**	*+* *1.9%*	*+* *5.3%*
	F4. APMs and street earnings		**50**	**12.2%**	*+* *1.2%*	*+* *5.8%*
	F5. No specific type of APM mentioned		**83**	**20.2%**	*+* *1.3%*	*−* *10.6%*
**G. Data collection**	G1. Manually collected data	0.8291	**128**	**31.2%**	*−* *3.6%*	*−* *1.0%*
	G2. Machine-collected data		**79**	**19.3%**	*+* *2.8%*	*+* *7.0%*
	G3. Manually collected and machine-collected data		**51**	**12.4%**	*+* *1.2%*	*+* *5.5%*
	G4. No examination of empirical data		**145**	**35.4%**	*−* *0.7%*	*−* *13.0%*
	G5. Not specified		**7**	**1.7%**	*+* *0.2%*	*+* *1.4%*

Another *third* dimension in which our SLR extends previous literature reviews is the strict adherence of a transparent and intersubjective process for evaluating the identified APM literature. In contrast to previous rather subjective traditional authorship reviews, the SLR methodology applied in our paper follows a replicable procedure for the systematic in-depth analysis of a defined body of APM literature that follows predefined steps, including reliability tests as well as measures to ensure validity (Massaro et al. 2016). Therefore, SLR methodology allows to minimise researcher idiosyncrasies and thus facilitates a decided derivation of research gaps based on a reliable and valid analytical framework.

*Fourth*, in contrast to previous literature reviews, we place further analytical emphasis on research subjects such as the research location, accounting regimes, different types of APMs and data collection methods. Moreover, when evaluating the 410 articles identified in the literature search, we use a comprehensive analytical framework which is essentially based on similar analytical frameworks already proven in other (structured) literature reviews in accounting research (e.g., Massaro et al. 2015; Serenko and Dumay 2015; Dumay and Garanina 2013). In order to facilitate a decided derivation of future research paths, we test our analytical framework for reliability and establish measures to ensure validity. This approach enables us to draw conclusions not only on the content, but also on the quantitative distribution of the articles on the different research topics analysed. And finally, unlike other literature reviews, in our SLR on APMs we also analyse bibliographic aspects such as ‘citation classics’ or the ‘superstar’ (‘Mathew’) effect.

The *results* of our SLR show that in the last decade a substantial amount of research has been conducted and published in the field of APM research. In particular, since 2010, the number of published articles on APMs has increased sharply reaching an all-time high in 2020 and 2021, indicating that the relevance of APM research has increased substantially over the last decade. Moreover, our SLR provides evidence that the phenomenon of voluntary reporting APMs has been the subject of numerous research articles in academic journals as well as professional journals, with a focus on U.S. GAAP data in the North American region (‘U.S. setting’). Previous studies in the APM field draw their motivation in particular from the different economic explanations for the voluntary reporting of APMs (‘informative’ vs. ‘strategic’ reporting). Accordingly, the *research focus* of these studies is on the investigation of management motives for voluntary disclosure of APMs as well as their perception by external stakeholders (e.g., analysts or non-professional investors). However, in recent years, an increasing number of studies on regulation of APM reporting, particularly in the U.S., have been published. With regard to the *research methods* applied, the results show a high concentration on empirical methods, in particular archival (database or archive) research, whereas other empirical methods are used much less frequently. In addition, the classification of the articles according to the types of APMs shows that a major focus of previous research has been on the analysis of company-reported APMs, including unaudited voluntary APM disclosures by companies on the one hand and APM disclosures in mandatory financial reports on the other. By contrast, the use of street earnings as a proxy for company-reported APMs has declined in recent years. Finally, the classification according to the data collection method reveals that both manual data collection (i.e., hand-collection of APMs disclosed in company publications) as well as database queries play a significant role in APM research.

In line with the objective of our SLR, as *key takeaways* we would like to emphasise three research topics for future APM research. *First, *we suggest more research on APM disclosures by companies in Europe, especially in light of ESMA’s recent regulatory initiatives, and the Asian region, mainly because of its increased economic power over the last two decades. *Second*, we recommend more research on APM reporting in the IFRS accounting regime (‘IFRS setting’). From the analysis of APMs in an IFRS setting conclusions can be drawn for their further development, in particular with regard to the current regulatory initiative of the *International Accounting Standards Board* (IASB) on the presentation of a company’s performance in the income statement and the disclosures of so-called ‘management performance measures’ (MPMs) in the accompanying notes according to *ED/2019/7 General Presentation and Disclosures* (ED/2019/7) (IASB 2019). *Third*, we propose to synthesise the previous findings of APM research. Two interesting methods that we believe are particularly useful for synthesising the previous research findings in the field of APM are meta-analyses and co-citation analyses.

Our SLR on APMs is structured as follows. Sect. 2 is devoted to the theoretical background of APM reporting. This includes a definition and delimitation of APMs (Sect. 2.1), a short discussion of the economic explanations for voluntary APM disclosures (Sect. 2.2) and a brief outline of the regulatory requirements for APM reporting in the U.S. and in Europe (Sect. 2.3). Sect. 3 provides a description of the SLR methodology applied in this paper. Sect. 4 presents the findings of our SLR. In Sect. 5 we discuss future research paths and in the following Sect. 6, we draw our conclusions. Finally, Sect. 7 describes the limitations inherent in SLR methodology, which must be considered when interpreting our results.

## Theoretical Background

### Definition and Delimitation of APMs

There is no universal and conclusive definition of APMs, but usually the voluntary nature of their disclosure is used to distinguish APMs from GAAP measures (Aubert and Grudnitski 2014; Hitz 2010a). This differentiation of APMs from statutory accounting is expressed in their characterisation as so-called ‘non-GAAP’ (financial) information (U.S. Congress 2002). Typically, APMs are published outside statutory financial reporting and generally not subject to mandatory audit (Bini et al. 2020; Aubert and Grudnitski 2020). The objective of calculating APMs is, at least superficially, to transform a GAAP earnings measure (e.g., line items of the income statement) into a performance measure which exceeds or at least supplements the information content of the respective GAAP measure by excluding certain components (usually expenses) (Hitz 2010a; Hitz and Jenniges 2008). For this purpose, GAAP earnings measures are presented ‘pro forma’ as if certain income and expenses had not been incurred in the income statement (Andersson and Hellman 2007; Heiden 2006).^4^ This typically involves adjusting GAAP earnings for non-recurring, unusual or non-operating components in order to obtain a persistent earnings measure (Hitz 2010a; Bhattacharya et al. 2003; Schiff and Schiff 2003). In addition, cash-effectiveness is sometimes used as a criterion to adjust GAAP earnings for non-cash transactions (Hitz 2010a; Entwistle et al. 2006b; Mulford and Comiskey 2005).

In the literature, various types of adjusted GAAP earnings measures are referred to as APMs (Hitz 2010a).^5^ Differences exist in particular with regard to so-called ‘earnings before measures’ (EB measures), which are typically not considered as original APMs (e.g., Black et al. 2017a; Bhattacharya et al. 2007; Allee et al. 2007). For this reason, following Hitz (2010a), a distinction is made in the following between APMs in the narrow sense and APMs in the broader sense.^6^ APMs in the narrow sense are performance measures which adjust GAAP earnings for specific earnings components based on management’s subjective assessments, sometimes without following a standardised calculation scheme (Hitz 2010a; similarly Sellhorn et al. 2014).^7^ Accordingly, it is typically not possible for external stakeholders to fully comprehend the calculation of APMs in the narrow sense without additional disclosures, such as reconciliations to the most directly comparable GAAP measure (similarly Reimsbach 2014; Sellhorn et al. 2014; Hitz 2010a). Examples of APMs in the narrow sense are ‘earnings before exceptional items’ or ‘adjusted operating earnings’.

APMs in the broader sense also include EB measures which systematically adjust GAAP earnings for interest expenses or income (*i*), taxes (*t*), depreciation (*d*) and/or amortisation (*a*) (Hitz 2010a; Sellhorn et al. 2014; Hummel and Beeler 2013). The resulting earnings measures of the so-called ‘EBIT family’, such as EBT, EBIT, EBITA or EBITDA, are well established, especially in external financial reporting of large, listed companies (Ruhwedel et al. 2018; Hitz 2010a; Hitz and Jenniges 2008). Sometimes EB measures, in particular EBT and EBIT, are disclosed as subtotals on the face of the income statement or can at least be calculated by external stakeholders based on the information provided in the accompanying notes to financial statements. Thus, EB measures are to be regarded as part of statutory accounting (Hitz 2010a; similarly Aubert and Grudnitski 2020; Sellhorn et al. 2014). However, different interpretations and/or different ways of calculating, for example, with regard to interests (e.g., adjustment of interest expenses only vs. adjustment of interest expenses and income) or taxes (e.g., adjustment of income taxes only vs. adjustment of income and other taxes), may lead to differently determined EB measures which are not suitable for cross-company and possibly also for intertemporal comparability (Reimsbach 2014). This is particularly the case if specific earnings components are not adjusted uniformly (e.g., parity adjustment of expenses and income vs. imparity adjustment of expenses only) (Küting and Heiden 2003).

Another difference exists between company-reported APMs and so-called ‘street earnings’, which were originally used in early empirical studies as proxy for company-reported APMs (e.g., Bradshaw and Sloan 2002; Brown and Sivakumar 2003; Doyle et al. 2003). Similar to APMs, street earnings are earnings measures which modify GAAP earnings, but are provided by external service providers for financial analysis rather than by the companies themselves (Hitz 2010a; Kolev et al. 2008; Gu and Chen 2004). Typically, street earnings are calculated based on the combined estimates of analysts covering a particular stock (so-called ‘consensus estimate’) (Thomson Reuters 2009).^8^ Accordingly, street earnings represent independent performance measures in the market which are to be explicitly distinguished from company-reported APMs, particularly because analysts have a different level of information compared to company insiders and are also subject to autonomous publication incentives (Barth et al. 2012; Hitz 2010a; Baik et al. 2009). Such publication incentives of analysts are not discussed further. Instead, our SLR considers street earnings solely in terms of their use as a proxy for company-reported APMs.

### Explanations for Voluntary APM Disclosures: Informative vs. Strategic Reporting

As voluntary disclosures, APM reporting incurs additional costs and thus requires explanation (Hitz 2010a). From previous APM literature, two alternative explanations (i.e., motives) for voluntary APM reporting can be derived with the information motive on the one hand (e.g., Entwistle et al. 2010; Lougee and Marquardt 2004; Bhattacharya et al. 2003) and the strategic motive of (adverse) investor influence on the other hand (e.g., Doyle et al. 2003; Frankel et al. 2011; Brown et al. 2012b), which are difficult, if not impossible, for external stakeholders to disentangle (Miller 2014; similarly Gronewold and Sellhorn 2009). According to the information motive, management’s objective in voluntarily reporting APMs is to provide decision-useful information to external stakeholders supplementing GAAP information. Following the information motive, the perceived deficit of GAAP earnings not, or not sufficiently, fulfilling the objective of providing decision-useful information is to be remedied by voluntary reporting of APMs (Hitz 2010a; see also Wagenhofer and Dücker 2007; Schipper and Vincent 2003). Therefore, according to the information motive, the objective of APM reporting is to calculate a consistent, predictable (i.e., extrapolatable) earnings measure which contains only components that are expected to recur in future reporting periods (Hitz 2010a), such as ‘core earnings’ (e.g., Wieland et al. 2013; Bhattacharya et al. 2003) or ‘persistent earnings’ (e.g., Venter et al. 2013; Choi et al. 2007). This is particularly the case because such earnings measures are assumed to be more comparable between companies and across reporting periods (Hitz 2010a; Andersson and Hellman 2007; Bhattacharya et al. 2004). Furthermore, it is assumed that APMs provided by management are of higher quality due to existing information asymmetries, since management as a company insider has an information advantage (i.e., ‘private’ information) regarding the persistence of certain earnings components compared to external stakeholders (Bradshaw and Sloan 2002).

The ‘dark side’ of APM reporting consists in the motive of (adversely) influencing investors (Sellhorn et al. 2014; Hitz 2010a). This motive is reflected in management’s intention to negatively (i.e., purposefully one-sided) influence investors’ assessment of a company’s earnings situation (Curtis et al. 2014; Hitz 2010a; Entwistle et al. 2006b). Among the instruments of aggressively (i.e., strategically) motivated APM reporting discussed in the literature are, for example, the exclusion of predominantly negative earnings components (Gronewold and Sellhorn 2009) and the emphasis of APMs relative to GAAP earnings (Allee et al. 2007; Bowen et al. 2005). Possible (mis)incentives to deliberately influence investors may arise in particular from the share-based compensation of management (Hitz 2010a). With the voluntary, unaudited reporting of APMs, management may have a potential instrument to strategically manage the perceived earnings performance and thus negatively influence investors (Hitz 2010a). This is the reason that has earned APMs epithets such as “Everything but Bad Stuff” (Turner 2000; similarly White 2015).

However, as already pointed out by Hitz (2010a), the two motives for APM reporting discussed in the literature are obviously not free of overlap even on a theoretical level, since, for example, the orientation of company-reported APMs on the calculation schemes of analysts’ street earnings forecasts is in line with an investor-oriented, value-enhancing reporting strategy, but nevertheless leads to a potential suppression of value-relevant private information by management. However, previous empirical studies find evidence suggesting that “both motives likely co-exist with the particular driver varying across firms and time conditional on prevailing reporting incentives” (Young 2014). Therefore, from a regulatory perspective, the dilemma for standard setters and regulators is to reduce information asymmetries by allowing management a certain degree of freedom in reporting APMs, while at the same time restricting this degree of freedom in reporting in a way that prevents aggressive APM disclosures (Young 2014; similarly Henry et al. 2020b).

### Regulation of APM Reporting

#### United States

In light of concerns regarding the quality of external financial reporting arising from accounting scandals and in response to numerous concerns about the potential for abuse (e.g., Turner 2000), first regulatory measures on the reporting of non-GAAP financial measures (NGFMs) were enacted in the U.S. in the early 2000s. In July 2002, the *U.S. Congress* approved the *Sarbanes-Oxley Act* (SOX) aiming to discipline the reporting of NGFMs (U.S. Congress 2002). Through Sect. 401(b) of SOX, the SEC was mandated to develop regulations for the reporting of NGFMs in order to limit its potential for abuse. Thereupon, in January 2003, the SEC issued a regulatory package consisting of Reg. G as well as amendments to *Regulation S‑K* (Reg. S-K) and the *Exchange Act Form 20‑F *(Form 20-F) (SEC 2002). Reg. G governs the use of NGFMs in reporting by companies listed on U.S. stock exchanges and applies whenever a company discloses (material) information containing NGFMs, such as investor presentations or other documents provided on the investor relations website, including oral reporting (e.g., telephone conferences, TV or radio broadcasts) (SEC 2002). The regulatory content of Reg. G essentially consists of a specific reconciliation requirement that requires companies to reconcile the differences between disclosed NGFMs and the respective directly comparable GAAP measure, preferring a quantitative reconciliation statement (SEC 2002). In contrast to Reg. G, the amendments to Item 10(e) of Reg. S‑K and Form 20‑F are more comprehensive, detailed and specifically govern the reporting of NGFMs in SEC filings (SEC 2002). For example, Reg. S‑K and Form 20‑F specifically require that the GAAP measures most directly comparable to the disclosed NGFMs must be presented with equal or greater prominence (SEC 2002). In addition, management must justify the decision to report NGFMs and explain in detail the reasons why the reported NGFMs provide investors with useful information about a company’s financial condition and results of operations (SEC 2002).^9^

Following the implementation of Reg. G, the SEC continued to regulate the reporting of NGFMs. In June 2003, the SEC published *Frequently Asked Questions Regarding the Use of Non-GAAP Financial Measures* (FAQs) which are intended to further contribute to disciplining the reporting of NGFMs (SEC 2003). Besides the answers to frequently asked application questions, the FAQs contain advice on the practical interpretation of the Reg. G rules (SEC 2003). In this practical interpretation, the SEC took a relatively rigorous approach which initially seemed to discourage companies from reporting NGFMs in SEC filings (Di Fabio and Roncagliolo 2020; similarly Marques 2006; Entwistle et al. 2006b). Nevertheless, companies continued to make extensive use of NGFMs in publications other than SEC filings, especially in rather unregulated publications such as (earnings) press releases (Black et al. 2012). In order to encourage the use of NGFMs in SEC filings, the SEC sought to increase the flexibility of the regulatory requirements by publishing *Compliance and Disclosure Interpretations on Non-GAAP Financial Measures* (C&DIs) in January 2010, that replaced the FAQs published in 2003 (SEC 2018). The C&DIs clarify, for example, that companies are permitted to adjust for recurring earnings components when calculating NGFMs, provided that management deems such adjustments to be appropriate (SEC 2018). Following this liberalisation of the Reg. G rules and the resulting increase in aggressive presentation and inconsistent calculation of NGFMs in subsequent years, the C&DIs were revised repeatedly and published (most recently in April 2018) in updated versions (so-called ‘updated C&DIs’) (SEC 2018). The updated C&DIs clarify, for example, that NGFMs must be reported consistently over time and that asymmetric policies to exclude only expenses of a non-recurring, infrequent or unusual nature while not excluding comparable income are not permitted (SEC 2018).

Besides the SEC, the *Financial Accounting Standards Board* (FASB) is also closely monitoring the NGFM reporting practices and considers the way in which U.S. companies report NGFMs as a starting point for improving U.S. GAAP (Golden 2017). For example, certain non-GAAP adjustments might help the FASB to identify where improvements of U.S. GAAP could be considered (Golden 2017). One way for the FASB to learn from companies’ NGFM reporting is to identify cases where changes to U.S. GAAP would render the need for non-GAAP reporting obsolete (Golden 2017). This should also be seen in light of the fact that companies disclose some NGFM because investors actively request and shape such voluntary disclosures (Golden 2017). In this respect, a change in U.S. GAAP may in these cases potentially contribute to the development of a standardised approach which is more consistent with common reporting practices that investors find useful (Golden 2017). In other words, it would improve the decision-usefulness of financial reporting (Golden 2017). Against this background, the FASB initiated a research project on financial performance reporting that is specifically focused on evaluating different alternatives for requiring more subtotals or more disaggregation in the income statement (FASB 2021; Golden 2017). However, in December 2019, the FASB has directed the staff to pause work on this project to monitor the progress of FASB’s segment reporting project and the IASB’s primary financial statements project (see in detail Sect. 2.3.2), but it will remain on the FASB’s active technical agenda (FASB 2021).

#### Europe

In Europe, according to Art. 4 of the so-called ‘IAS Regulation’,^10^ listed companies are obliged to prepare and publish consolidated financial statements in accordance with the *International Financial Reporting Standards* (IFRS) endorsed by the European Commission. In IFRS accounting, the disclosure of APMs is of particular relevance, since international accounting standards contain numerous degrees of freedom with regard to the presentation and disclosure requirements, especially in the income statement (Pizzo 2020; Young 2014; Hitz and Jenniges 2008). In fact, IAS 1* Presentation of Financial Statements* (IAS 1) does not specify a uniform structure, but only prescribes certain line items that shall be included in the income statement without specifically defining them (IAS 1.82). This minimum classification scheme shall be extended to include additional line items (such as headings, subtotals and disaggregated line items), only if such presentation is relevant to an understanding of a company’s financial performance (IAS 1.85) and is therefore essentially at the discretion of management (Hitz and Jenniges 2008).^11^ Thus, at least formally, IFRS allow the reporting of certain EB measures on the face of the statutory income statement (Lüdenbach et al. 2020; Hitz and Jenniges 2008).^12^ Besides EB measures, IAS 33* Earnings per Share* (IAS 33) permits the presentation of non-GAAP earnings per share (EPS) measures on the face of the income statement or in the accompanying notes under certain conditions (IAS 33.73). In addition to the income statement, APMs are often found in segment reporting as part of the accompanying notes, since under IFRS 8 *Operating Segments* (IFRS 8) segment reporting is based on the ‘management approach’, which requires the segment result to be reported on the basis of internal organisation and reporting (IFRS 8.23; see in detail Schulz-Danso 2020; Lüdenbach et al. 2020). Furthermore, APMs are often reported in group management reports, which supplement IFRS consolidated financial statements. In Germany, for example, according to national regulations, a group management report must contain an analysis of the most significant financial performance indicators which are also used for the internal management of the group (‘management approach’; Sect. 290 (1) s. 1 HGB; GAS 20.102). Similar regulations generally also exist in other European countries in accordance with the EU’s competence to issue directives.

Due to the variety of APMs disclosed in IFRS financial statements and the tendency of companies to adjust common GAAP earnings, the IASB is currently discussing the need to define performance measures within IFRS (IASB 2017). For this purpose, IASB published ED/2019/7 in December 2019, which is intended to replace IAS 1 in the future (IASB 2019).^13^ The main focus of ED/2019/7 is on the presentation of a company’s performance in the income statement. According to ED/2019/7, the minimum classification scheme of the income statement is to be supplemented by four new categories ‘operating’, ‘integral associates and joint ventures’, ‘investing’ and ‘financing’ (IASB 2019). In addition, three new subtotals ‘operating profit or loss’, ‘operating profit or loss and income and expenses from integral associates and joint ventures’ and ‘profit or loss before financing and income tax’ shall be presented in the income statement (IASB 2019). However, since the proposed structure severely restricts the disclosure of individual company performance measures in the income statement, ED/2019/7 provides for mandatory disclosures of MPMs in a single note to the financial statements (IASB 2019).^14^ In addition to the disclosure of MPMs, further information must be provided in the accompanying notes. This, for example, includes a reconciliation between the MPMs disclosed and the most directly comparable (sub)total of the income statement (IASB 2019). These planned amendments to IAS 1 are expected to be applied from the financial year 2023 at the earliest (IASB 2019; Grimm and Heintges 2020).

Besides periodic financial reporting, APM reporting also takes place in event publicity, such as ad-hoc disclosures. In Europe, ESMA is responsible for regulating voluntary APM reporting of listed companies in publication media other than (consolidated) financial statements (Bini et al. 2020). Already in 2005, ESMA’s predecessor institution, the *Committee of European Securities Regulators* (CESR), issued recommendations on the reporting of APMs by listed companies as a first measure to regulate APM reporting in Europe (CESR 2005). However, these recommendations were replaced by the ESMA guidelines in October 2015 (ESMA 2015). The ESMA guidelines are to be applied since July 2016 for the reporting of APMs disclosed by issuers or persons responsible for the prospectus when publishing regulated information and prospectuses (ESMA 2015). As examples of regulated information, the ESMA guidelines mention management reports that must be disclosed under the *Transparency Directive*^15^ and publications under the *Market Abuse Regulation*,^16^ such as ad-hoc disclosures including financial earnings results (ESMA 2015). However, the ESMA guidelines explicitly do not apply to APM reporting in (half-year) consolidated financial statements and other additional regular financial information (ESMA 2015), meaning that a large proportion of the published APMs does not fall within the scope of the ESMA guidelines (Dinh et al. 2018). This exemption from the scope of the ESMA guidelines is due to the IASB’s planned amendments to IAS 1, which are intended to address the issue related to APM reporting (IASB 2019; see in detail Kleinmanns 2016). Essentially, the material scope of the ESMA guidelines is reduced to (group) management reports (ESMA 2015; see also Lüdenbach et al. 2020).

In terms of content, the ESMA guidelines show similarities to Reg. G. For example, according to the ESMA guidelines, companies shall disclose a reconciliation statement from the disclosed APMs to the most directly reconcilable line item, subtotal or total presented in the financial statements, separately disclosing and explaining the material reconciling items (ESMA 2015). Also, companies shall explain why reported APMs are considered useful and must not give APMs priority over GAAP measures in terms of their prominence, emphasis or authority (ESMA 2015). Besides the ESMA guidelines, in 2017 ESMA published *Questions and answers on ESMA Guidelines on Alternative Performance Measures* (Q&As) to answer questions from the public and competent authorities on the practical application of the ESMA guidelines to contribute to its consistent application (ESMA 2017). More recently, in April 2020, ESMA issued Q&As to provide guidance to companies on the application of ESMA guidelines in context of the COVID-19 pandemic (ESMA 2020).

Being defined as ‘guidelines’ under Art. 16 of the so-called ‘ESMA regulation’,^17^ the ESMA guidelines in themselves generally do not have a binding legal character. However, the intention to develop a binding legal character can be deducted both from the specific characteristics on the one hand and from the wording of the ESMA guidelines on the other (European Commission 2014; Van Rijsbergen 2014). Moreover, the ESMA guidelines are in line with the *European System of Financial Supervision* (ESFS), the supervisory structure at the European level that promotes supervisory practices and ensures consistent and uniform application of European law (Bini et al. 2020). Under this mechanism, Art. 16(3) of the ESMA regulation requires the national enforcement authorities of the European Member States to inform ESMA whether they comply or intend to comply with each guideline or recommendation issued by ESMA that applies to them within two months of the issuance of the respective guideline or recommendation (ESMA 2022). If a competent authority does not comply or does not intend to comply with the relevant ESMA guidelines or recommendations, it must inform ESMA of the reasons and ESMA then decides on a case-by-case basis whether to publish those reasons (ESMA 2022). As of January 2022, all 27 European Member States as well as the three so-called ‘EEA EFTA States’ (Norway, Liechtenstein and Iceland) confirmed to ESMA that they will monitor compliance of listed companies with ESMA guidelines (ESMA 2022).

## Research Method

### Structured Literature Review Methodology

**Fig. 1:**
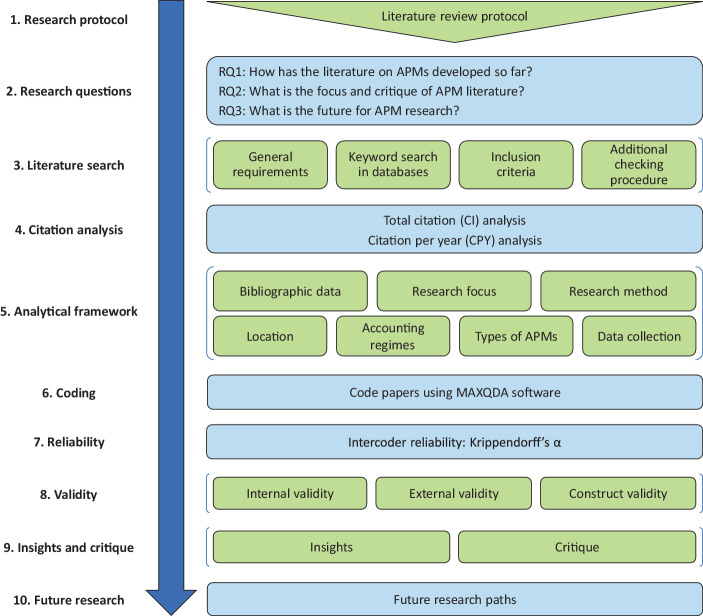
Structured literature review process according to Massaro et al. (2016)

In this paper, we apply SLR methodology according to Massaro et al. (2016). To develop our SLR on APMs, we use the (slightly modified) 10-step process proposed by Massaro et al. (2016), as shown in Fig. 1. According to this 10-step process, a *research protocol* was developed as a *first step* in order to ensure replicability of the SLR (Tranfield et al. 2003).^18^ In a *second step*, the three core *research questions* were defined (see Fig. 1), which draw upon the three tasks of critical research (‘insights’, ‘critique’ and ‘transformative redefinition’) according to Alvesson and Deetz (2000). In a *third step*, a comprehensive *literature search* was conducted (see in detail Sect. 3.2).

The *fourth step* of the SLR is the *citation analysis*. For measuring the impact of the identified articles, authors and journals, we use ‘citations’ (CI) and ‘citations per year’ (CPY) following Dumay (2014). Thereafter, CIs “are the total citation for the article … on a specific day” (Massaro et al. 2016) and CPY “is citations divided by the number of the years between the current year of analysis and publication year” (Massaro et al. 2016).^19^ Like Dumay (2014), we use citation data from *Google Scholar* (as opposed to *ISI Web of Science* or *Scopus*) for two reasons. First, because “Google Scholar … indexes all categories of publications, and counts citations from non-peer-reviewed works, such as practitioner journals, government documents, and newspapers” (Serenko and Bontis 2013; see also Dumay 2014). Second, because “Google Scholar is currently considered a leading tool in citation analysis” (Serenko and Bontis 2013; see also Harzing and van der Wal 2008). For these reasons, we downloaded the articles’ citation data from *Google Scholar* on 29 January 2022. Like in previous SLRs, the articles published within the period just before the SLR was conducted (2021) were excluded from all individual CPY scores because there was not enough time to cite the articles (see in detail Sect. 4.1 and *Annex 5*).

Since a SLR is essentially “a form of content analysis” (Massaro et al. 2015), the *fifth step* is to define categories which are embedded in an *analytical framework*. As advocated by Massaro et al. (2016), we develop a specific analytical framework, which is essentially derived from analytical frameworks that have already proven successful in other (structured) literature reviews in accounting research (e.g., Massaro et al. 2015; Serenko and Dumay 2015; Dumay and Garanina 2013). In order to ensure a precise delineation between the variables of the categories and a precise assignment of the articles, the analytical framework was subjected to a pre-test (similarly Broadbent and Guthrie 2008; Guthrie et al. 2012; Guthrie and Murthy 2009). During this pre-test, the 20 most cited articles were coded by one of the authors, resulting in a slightly modified analytical framework.^20^ Based on the modified analytical framework, the *sixth step* was the *coding* of the articles. In the coding process, one of the authors coded all articles based on the abstracts and, where necessary, the full text of the articles, using *MAXQDA* software. During this process, the categories were only slightly modified. The resulting final analytical framework is presented in Table 1 and individual categories will be explained in Sect. 4.

In order to ensure reliability of the analytical framework and the codes, we conducted *reliability* tests in a *seventh step*. We use *Krippendorff’s α* (*K‑alpha*) as reliability measure because it is robust (Krippendorff 2013; Hayes and Krippendorff 2007) and “can be used regardless of the number of observers, levels of measurement, sample sizes, and presence or absence of missing data” (Hayes and Krippendorff 2007). For the determination of *Krippendorff’s α*, the 382 articles initially included in the sample (before the additional checking procedure) was divided equally between two students enrolled in the master’s programme in ‘Service Management’ at a German university and coded by them independently. Afterwards, *Krippendorff’s α* was calculated for the independent data sets, resulting in the scores as shown in Table 1. For all seven categories, the values are above, or with regard to the categories ‘B. Research focus’ and ‘F. Types of APMs’ at least close to the target reliability measure of *Krippendorff’s α* ≥ 0.8. The average *Krippendorff’s α* is 0.910. Therefore, we assume that the analytical framework and the assigned codes can be considered reliable.

Besides reliability tests, we also establish measures to ensure *validity *in an *eighth step*. In order to establish internal validity, we built on analytical frameworks that have already proven successful in other (structured) literature reviews in accounting research (e.g., Massaro et al. 2015; Serenko and Dumay 2015; Dumay and Garanina 2013). In addition, the systematic coding and continuous logging of the evaluation process in *MAXQDA* ensures transparency and traceability and minimises researcher idiosyncrasies (similarly Winschel and Stawinoga 2019). Furthermore, internal validity is promoted by pre-testing the analytical framework (similarly Winschel and Stawinoga 2019). To establish external validity, the SLR on APMs strictly follows the SLR methodology as proposed by Massaro et al. (2016). Moreover, since the SLR took several months to complete, we implemented an additional checking procedure throughout the analysis, writing and peer-review process to further promote external validity (similarly Winschel and Stawinoga 2019). Construct validity is promoted by the structured approach for the derivation of study criteria as well as for the collection and evaluation of articles. Furthermore, construct validity is supported by using a variety of different keywords and databases, defining *inclusion criteria* to structure the data collection process and protocolising intermediate findings in our research protocol as part of the evaluation process (similarly Winschel and Stawinoga 2019).

### Literature Search and Sampling Strategy

As *third step *of our SLR on APMs, we conducted a comprehensive *literature search* in the five literature databases *Scopus, EconBiz, Google Scholar, EBSCO Business Source Premier* and *ISI Web of Science* by searching for keywords within titles, abstracts and keywords using phrase searches. The keywords used to search the databases were identified in advance in a rudimentary literature search. When conducting the literature search, the pre-determined keywords were supplemented with additional keywords identified and the databases were then searched again based on the newly identified keywords. Although in academic research, the peer-review process may be considered synonymous with the quality of published research (Easterby-Smith et al. 2012), according to Massaro et al. (2016) “other sources can be equally valid to review a particular field and … [t]hus, researchers should consider not confining SLRs solely to journal articles”. Given the importance of other types of publications in accounting research (e.g., Carmona 2006; Fülbier and Weller 2011, 2008; Wagenhofer 2006), we also take into account non-peer-reviewed literature. Thus, when searching the databases, we limited our selection criteria to the source types ‘academic journals’, ‘professional/practitioner journals’, ‘books’, ‘book chapters’ and ‘working/discussion/conference papers’.^21^ Furthermore, we limit our selection criteria to the relevant subject areas in the field of economics, as a search in all subject areas returns too many thematically inappropriate search results that do not substantively focus on APMs. In order to include all articles published by 31 December 2021 lately, no time limits were set. A complete list of the selected keywords, the search strings used to search the databases (including the subject areas) and the number of search results is provided in *Annex 2*.

Besides the international literature, we also include research papers written in German, providing insights into German-language APM research. In order to counterbalance the tendencies that may arise from the inclusion of articles written in German and the inclusion of research articles not published in academic journals, we provide scenario analyses when presenting the results of our SLR (see Table 1). In this context, for the delineation between ‘academic journals’ and ‘professional journals’, we refer to the *VHB-JOURQUAL3*, a ranking of German and international journals from 2014, which is based on a survey of more than 1,100 members of the *German Academic Association of Business Research* (VHB 2015). We consider a journal as ‘academic journal’ if the respective journal is ranked at least ‘C’ by the *VHB-JOURQUAL3* ranking (i.e., in categories ‘A+’ to ‘C’). Conversely, we consider a journal as ‘professional journal’ if the respective journal is ranked ‘D’ or below (i.e., in categories ‘D’ to ‘E’). However, for articles published in an international journal not included in the *VHB-JOURQUAL3* ranking, the classification is based on the information provided on the respective journal’s website. In these cases, we only classify a journal as ‘academic journal’ if the submission process of the respective journal demonstrably includes an anonymised double-blind peer review process.

To be included in our sample, the articles identified in the literature search must fulfil some general requirements (*inclusion criteria*). First, the articles included in the sample must be written in English or German. Second, the research topic of the article must substantively focus on APMs. For the application of the second criterion, the title, abstract and, where necessary, full text of the articles were reviewed in detail to determine whether they address the research topic of APMs in terms of content. Although typically not considered APMs in empirical studies (e.g., Black et al. 2017a; Bhattacharya et al. 2007; Allee et al. 2007), our keyword search also considers EB measures. First, because a clear distinction between EB measures and APMs is not always possible, especially when APMs are determined based on EB measures (e.g., ‘EBITDA before special items’ or ‘sustainable EBIT’). Second, because we intend the SLR to contribute to the current debate of the IASB on the reporting of performance measures (see Sect. 2.3.2). Articles not included in the sample due to the application of the *inclusion criteria* were documented in a *Microsoft Excel* file along with the reason for exclusion.

The literature search resulted in a total of 2,337 articles. Of these, 1,927 articles were removed from the sample due to the application of the *inclusion criteria*, leaving a sample with a total of 410 articles.^22^ For all 410 articles included in the sample a full version was obtained and stored in an *EndNote* database with complete referencing details (similarly Guthrie et al. 2012; Guthrie and Murthy 2009; Broadbent and Guthrie 2008). Furthermore, in order to complete the sample, an additional checking procedure was conducted in which the five databases were continuously searched for newly published articles throughout the analysis, writing and peer-review process (similarly Winschel and Stawinoga 2019), resulting in 52 articles being added to the final sample of 410 articles.

## Results: Insights and Critique

### Evolution of the Literature on APMs (Articles, Years and Citation Analysis)

In this Sect. 4.1, the findings on the evolution of APM literature are presented in order to answer *RQ1* (*How has the literature on APMs developed so far?*). To answer this question, we use the findings on the number of articles, years and citation data.^23^ However, one problem that goes along with the use of CI is that “older articles have a greater opportunity of being cited” (Aguinis et al. 2010). In order to counterbalance this tendency, the citation analysis provided in this Sect. 4.1 is primarily based on CPYs (similarly Dumay 2014; Massaro et al. 2015; Dumay et al. 2016).

**Fig. 2:**
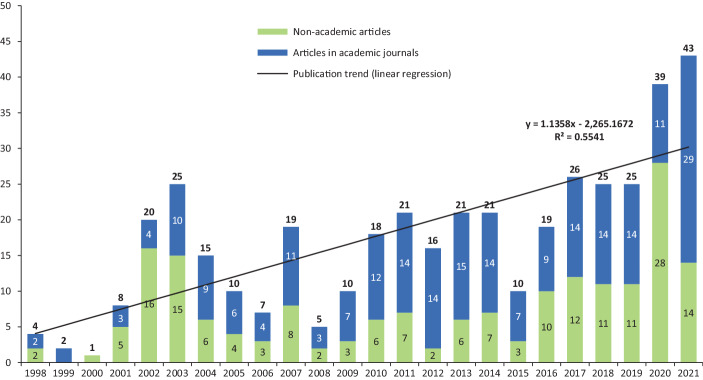
Number of articles on APMs and publication trend

The results of the literature search show an increase in the number of articles on APMs, especially beginning from the 2000s onwards (see Fig. 2). Although there is a decline in the following years, from 2010 onwards the number of articles rises sharply, reaching a peak of 39 articles in 2020 and 43 articles in 2021. However, of the 39 articles published in 2020, 16 articles are contributions from a single collective work (Moscariello and Pizzo 2020). Across all years, a total of 410 articles could be identified. Of these, almost two thirds (64.9%) were published after 2010, indicating an increasing publication trend and thus an increased relevance of APM research. This may be associated with various reasons. On the one hand, the increase in impairment and restructuring charges included in GAAP earnings because of the economic downturn during the global economic crisis from 2007 onwards may have led to more companies reporting APMs, either to disguise poor earnings performance or to better inform investors about the persistence of earnings during the economic downturn (similarly Webber et al. 2013). On the other hand, companies in the U.S. may be less concerned about SEC scrutiny, especially after the liberalisation of Reg. G rules by the C&DIs in 2010 (similarly Webber et al. 2013). These developments in APM reporting practice may be reflected in the number of articles on APMs and the publication trend with a certain time lag, due to the period from companies reporting APMs to the publication of articles in journals (e.g., for time consuming data collection and analysis as well as the publication process).

Since 2001, articles in academic journals (‘academic articles’) and articles in professional journals (‘professional articles’) have both been published in each year. Across all years, about half of the sample is composed of academic articles (55.6%) and professional articles (44.4%), indicating that besides academic research, the APM topic is of particular interest to practitioners. Of all 182 non-academic articles, three fifths (60.4%) were published in professional journals, with the English-language ‘CPA Journal’ (19 articles) having the highest number of published articles, followed by the German-language ‘Zeitschrift für Internationale Rechnungslegung’ (IRZ; 17 articles). With regard to the remaining two fifths (39.6%) of all non-academic articles, these are hitherto unpublished working/discussion/conference papers (18.2%), contributions to collective works (12.6%) and monographs (8.8%).

Among the sample, 25 articles have at least four times the arithmetic mean for CPYs (see Table 2). For CIs, 21 articles have at least four times the arithmetic mean (see Table 3). As can be seen from the two tables, there are 17 articles common to both rankings (see the italicised articles in Tables 2 and 3). Consequently, these frequently cited works may be considered ‘citations classics’ in the field of APM research (Garfield 1989). Among these ‘citation classics’, the article by Hirshleifer and Teoh (2003), which offers an approach to analytical modelling of financial reporting and disclosure, stands out as having by far the highest number of citations for both CPYs and CIs. Furthermore, when analysing CPYs, it becomes obvious that more recent articles appear in Table 2 with highest CPYs. The CPY analysis thus suggests that there is an interest among researchers to cite the latest articles on APM research.^24^ In particular, the articles by Christensen et al. (2019) and Anilowski Cain et al. (2020) are both included in the ‘top 25’ articles with the highest CPYs, despite being recently published. Besides the articles in Table 2, the articles by Black et al. (2021a) and Curtis et al. (2021) have already been cited 56 times each.^25^ Given the time lag between submission until publication, it is extraordinary that these two articles received this many citations shortly after publication, suggesting that they have already had an impact on the academic debate. In terms of journal ranking, 12 out of the 17 articles considered ‘citation classics’ were published in academic journals with highest ranking (A+) according to the *VHB-JOURQUAL3* ranking and are considered to be “excellent and global leading scientific journals in business research” (VHB 2021). Among these journals, the ‘Accounting Review’ stands out, publishing two fifths (41.2%) of all articles considered ‘citation classics’, suggesting that articles published in highly ranked journals according to the *VHB-JOURQUAL3* ranking tend to have a greater impact on the research field. In terms of authors, *Theodore E. Christensen* sticks out, having co-authored more than a third of all 25 articles (36.0%) with the highest CPYs.

**Table 2:** ‘Top 25’ articles on APMs with the highest CPYs by *Google Scholar *at least four times above average (5.05)

	Article	Title	Journal	JQ3	CPY	CI	CPY/∅	CI/∅
*1*	*Hirshleifer and Teoh *(2003)	*Limited attention, information disclosure, and financial reporting*	*Journal of Accounting and Economics*	*A+*	*114.1*	*2053*	*22.6*	*39.8*
*2*	*Badertscher *(2011)	*Overvaluation and the Choice of Alternative Earnings Management Mechanisms*	*Accounting Review*	*A+*	*57.4*	*574*	*11.4*	*11.1*
3	Bentley et al. (2018)	Disentangling Managers’ and Analysts’ Non-GAAP Reporting	Journal of Accounting Research	A+	53.7	161	10.6	3.1
4	Black et al. (2018)	Non-GAAP reporting: Evidence from academia and current practice	Journal of Business Finance & Accounting	B	52.3	157	10.4	3.0
*5*	*Bradshaw and Sloan *(2002)	*GAAP versus The Street: An Empirical Assessment of Two Alternative Definitions of Earnings*	*Journal of Accounting Research*	*A+*	*50.5*	*960*	*10.0*	*18.6*
*6*	*Palmrose and Scholz *(2004)	*The Circumstances and Legal Consequences of Non-GAAP Reporting: Evidence from Restatements*	*Contemporary Accounting Research*	*A*	*45.7*	*777*	*9.0*	*15.1*
*7*	*Doyle et al. *(2013)	*Do managers define non-GAAP earnings to meet or beat analyst forecasts?*	*Journal of Accounting and Economics*	*A+*	*39.9*	*319*	*7.9*	*6.2*
*8*	*Bhattacharya et al. *(2003)	*Assessing the relative informativeness and permanence of pro forma earnings and GAAP operating earnings*	*Journal of Accounting and Economics*	*A+*	*34.0*	*612*	*6.7*	*11.9*
9	Bradshaw et al. (2018)	Analysts’ GAAP earnings forecasts and their implications for accounting research	Journal of Accounting and Economics	A+	34.0	102	6.7	2.0
*10*	*Doyle et al. *(2003)	*The Predictive Value of Expenses Excluded from Pro Forma Earnings*	*Review of Accounting Studies*	*A*	*31.8*	*572*	*6.3*	*11.1*
*11*	*Lougee and Marquardt *(2004)	*Earnings Informativeness and Strategic Disclosure: An Empirical Examination of “Pro Forma” Earnings*	*Accounting Review*	*A+*	*30.3*	*515*	*6.0*	*10.0*
*12*	*Bowen et al. *(2005)	*Emphasis on Pro Forma versus GAAP Earnings in Quarterly Press Releases: Determinants, SEC Intervention, and Market Reactions*	*Accounting Review*	*A+*	*27.8*	*444*	*5.5*	*8.6*
13	Leung and Veenman (2018)	Non-GAAP Earnings Disclosure in Loss Firms	Journal of Accounting Research	A+	27.3	82	5.4	1.6
14	Black et al. (2017b)	The Relation Between Earnings Management and Non-GAAP Reporting	Contemporary Accounting Research	A	27.3	109	5.4	2.1
*15*	*Brown et al. *(2012b)	*Investor Sentiment and Pro Forma Earnings Disclosures*	*Journal of Accounting Research*	*A+*	*26.8*	*241*	*5.3*	*4.7*
*16*	*Elliott *(2006)	*Are Investors Influenced by Pro Forma Emphasis and Reconciliations in Earnings Announcements?*	*Accounting Review*	*A+*	*26.5*	*397*	*5.2*	*7.7*
*17*	*Frederickson and Miller *(2004)	*The Effects of Pro Forma Earnings Disclosures on Analysts’ and Nonprofessional Investors’ Equity Valuation Judgements*	*Accounting Review*	*A+*	*25.8*	*438*	*5.1*	*8.5*
18	Curtis et al. (2014)	The Disclosure of Non-GAAP Earnings Information in the Presence of Transitory Gains	Accounting Review	A+	23.6	165	4.7	3.2
19	Christensen et al. (2019)	Non-GAAP reporting following debt covenant violations	Review of Accounting Studies	A	21.5	43	4.3	0.8
*20*	*Kolev et al. (*2008*)*	*SEC Scrutiny and the Evolution of Non-GAAP Reporting*	*Accounting Review*	*A+*	*21.1*	*274*	*4.2*	*5.3*
*21*	*Frankel et al. *(2011)	*Non-GAAP earnings and board independence*	*Review of Accounting Studies*	*A*	*21.0*	*210*	*4.2*	*4.1*
22	Anilowski Cain et al. (2020)	Detecting Opportunistic Special Items	Management Sience	A+	21.0	21	4.2	0.4
*23*	*Black and Christensen *(2009)	*US Managers’ Use of ‘Pro Forma’ Adjustments to Meet Strategic Earnings Targets*	*Journal of Business Finance & Accounting*	*B*	*20.4*	*245*	*4.0*	*4.8*
*24*	*Brown and Sivakumar *(2003)	*Comparing the Value Relevance of Two Operating Income Measures*	*Review of Accounting Studies*	*A*	*20.4*	*367*	*4.0*	*7.1*
*25*	*Bhattacharya et al. *(2007)	*Who Trades on Pro Forma Earnings Information?*	*Accounting Review*	*A+*	*20.3*	*284*	*4.0*	*5.5*

**Table 3:** ‘Top 21’ articles on APMs with the highest CIs by *Google Scholar *at least four times above average (51.57)

	Article	Title	Journal	JQ3	CPY	CI	CPY/∅	CI/∅
*1*	*Hirshleifer and Teoh *(2003)	*Limited attention, information disclosure, and financial reporting*	*Journal of Accounting and Economics*	*A+*	*114.1*	*2053*	*22.6*	*39.8*
*2*	*Bradshaw and Sloan *(2002)	*GAAP versus The Street: An Empirical Assessment of Two Alternative Definitions of Earnings*	*Journal of Accounting Research*	*A+*	*50.5*	*960*	*10.0*	*18.6*
*3*	*Palmrose and Scholz *(2004)	*The Circumstances and Legal Consequences of Non-GAAP Reporting: Evidence from Restatements*	*Contemporary Accounting Research*	*A*	*45.7*	*777*	*9.0*	*15.1*
*4*	*Bhattacharya et al. *(2003)	*Assessing the relative informativeness and permanence of pro forma earnings and GAAP operating earnings*	*Journal of Accounting and Economics*	*A+*	*34.0*	*612*	*6.7*	*11.9*
*5*	*Badertscher *(2011)	*Overvaluation and the Choice of Alternative Earnings Management Mechanisms*	*Accounting Review*	*A+*	*57.4*	*574*	*11.4*	*11.1*
*6*	*Doyle et al. *(2003)	*The Predictive Value of Expenses Excluded from Pro Forma Earnings*	*Review of Accounting Studies*	*A*	*31.8*	*572*	*6.3*	*11.1*
*7*	*Lougee and Marquardt *(2004)	*Earnings Informativeness and Strategic Disclosure: An Empirical Examination of “Pro Forma” Earnings*	*Accounting Review*	*A+*	*30.3*	*515*	*6.0*	*10.0*
*8*	*Bowen et al. *(2005)	*Emphasis on Pro Forma versus GAAP Earnings in Quarterly Press Releases: Determinants, SEC Intervention, and Market Reactions*	*Accounting Review*	*A+*	*27.8*	*444*	*5.5*	*8.6*
*9*	*Frederickson and Miller *(2004)	*The Effects of Pro Forma Earnings Disclosures on Analysts’ and Nonprofessional Investors’ Equity Valuation Judgements*	*Accounting Review*	*A+*	*25.8*	*438*	*5.1*	*8.5*
*10*	*Elliott *(2006)	*Are Investors Influenced by Pro Forma Emphasis and Reconciliations in Earnings Announcements?*	*Accounting Review*	*A+*	*26.5*	*397*	*5.2*	*7.7*
*11*	*Brown and Sivakumar *(2003)	*Comparing the Value Relevance of Two Operating Income Measures*	*Review of Accounting Studies*	*A*	*20.4*	*367*	*4.0*	*7.1*
12	Gu and Chen (2004)	Analysts’ treatment of nonrecurring items in street earnings	Journal of Accounting and Economics	A+	18.9	322	3.7	6.2
*13*	*Doyle et al. *(2013)	*Do managers define non-GAAP earnings to meet or beat analyst forecasts?*	*Journal of Accounting and Economics*	*A+*	*39.9*	*319*	*7.9*	*6.2*
*14*	*Bhattacharya et al. *(2007)	*Who Trades on Pro Forma Earnings Information?*	*Accounting Review*	*A+*	*20.3*	*284*	*4.0*	*5.5*
*15*	*Kolev et al. *(2008)	*SEC Scrutiny and the Evolution of Non-GAAP Reporting*	*Accounting Review*	*A+*	*21.1*	*274*	*4.2*	*5.3*
16	Heflin and Hsu (2008)	The impact of the SEC’s regulation of non-GAAP disclosures	Journal of Accounting and Economics	A+	19.0	247	3.8	4.8
*17*	*Black and Christensen *(2009)	*US Managers’ Use of ‘Pro Forma’ Adjustments to Meet Strategic Earnings Targets*	*Journal of Business Finance & Accounting*	*B*	*20.4*	*245*	*4.0*	*4.8*
*18*	*Brown et al. *(2012b)	*Investor Sentiment and Pro Forma Earnings Disclosures*	*Journal of Accounting Research*	*A+*	*26.8*	*241*	*5.3*	*4.7*
19	Bhattacharya et al. (2004)	Empirical Evidence on Recent Trends in Pro Forma Reporting	Accounting Horizons	B	13.7	233	2.7	4.5
20	Marques (2006)	SEC interventions and the frequency and usefulness of non-GAAP financial measures	Review of Accounting Studies	A	14.5	217	2.9	4.2
*21*	*Frankel et al. *(2011)	*Non-GAAP earnings and board independence*	*Review of Accounting Studies*	*A*	*21.0*	*210*	*4.2*	*4.1*

### Focus and Critique of the APM Literature

The structured approach of the SLR aims to answer specific pre-defined questions rather than just summarising the entire body of knowledge (Petticrew and Roberts 2006). Therefore, Sect. 4.2 intends to answer RQ2 (*What is the focus and critique of APM literature?*) by determining the focus of previous APM research. For this purpose, the results in Table 1 are presented in detail.

#### Research Focus

**Fig. 3:**
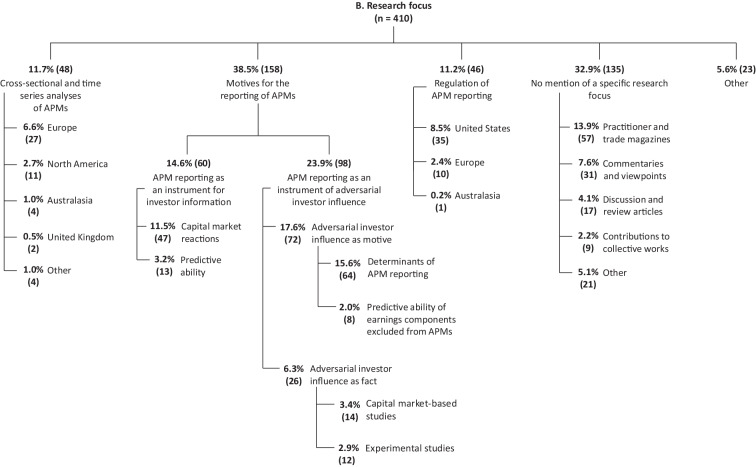
Research focus of the articles on APMs

The subcategories of the research focus category are based on the classification of research articles on APM reporting according to Hitz (2010a). This categorisation was revised inductively during the coding process and slightly adjusted. As shown in Fig. 3, the focus of APM research activities to date has essentially been on three basic interests: *(i) cross-sectional and time series analyses of APMs, (ii) motives for the reporting of APMs *and* (iii) regulation of APM reporting*. Although the coding is based on the primary research focus of the articles as stated by the authors, the results on subordinate (i.e., secondary, tertiary, etc.) research foci are also considered in the following.^26^

##### (i) Cross-Sectional and Time Series Analyses of APMs

Cross-sectional and time series analysis is addressed in 11.7% of the articles, especially in the early stages of APM research. Specifically, these articles address questions of prevalence, quantitative characteristics and the evolution of APMs over time. First-published articles in this category are mainly focused on APM reporting in *North America* (2.7%), especially in the U.S. (2.2%). Overall, these studies for the North American region show that the frequency and scope of APM reporting increased significantly beginning in the early 1980s (e.g., Bhattacharya et al. 2004; Zhang and Zheng 2011). For example, Entwistle et al. (2005) demonstrate that 77% of S&P 500 companies report pro forma earnings in their year-end earnings press releases for financial years ending between February 2001 and January 2002. However, there is consistent evidence of a significant decline in APM reporting after the introduction of Reg. G in 2003, but this was rather short-term in nature and started to increase again in subsequent years after Reg. G came into force, at the latest from the liberalisation of Reg. G rules by the C&DIs in 2010 (see in detail the findings on *(iii) regulation of APM reporting*).

Besides the North American region, most of the 48 articles (11.7%) conducting cross-sectional and time-series analysis deal with APM reporting in *Europe* (6.6%), especially in Germany (3.7%). This, however, is mainly due to the inclusion of German-language articles in our total sample. When looking at the subsample including only international articles, the proportion of articles conducting cross-sectional and time-series analysis for Germany would amount to only 1.0%. This is because of the 15 articles (3.7%) conducting cross-sectional and time-series analysis for Germany, the vast majority (13 out of 15 articles) are written in German. Besides Germany, there are only a few articles dealing with APM reporting in the European context, namely in Switzerland (1.0%), Austria (0.5%) and the Czech Republic (0.2%). In addition, six articles (1.5%) analyse APM reporting of European companies from several countries, such as companies listed in the *EURO STOXX 50* index (e.g., Kriete et al. 2003). Similar to the findings for the North American region, descriptive evidence from European companies supports the proposition that APM reporting is a well-established part of capital market communication, especially among large, listed companies (similarly Coulton et al. 2016).

Besides the two most studied regions, North America and Europe, there are a handful of articles that conduct cross-sectional and time-series analyses for the *Australasian* region (1.0%) and the *United Kingdom* (0.5%), most of which show comparable results. For example, the CFA Society (2015) documents that the majority of FTSE 100 companies uses IFRS-adjusted measures and that over the ten-year period from 2005 to 2014, the aggregate adjusted net profit was 17% higher compared to the IFRS equivalent. Furthermore, through a survey of its members (i.e., investment professionals), the CFA Society (2015) found that 61% of respondents routinely use management-reported IFRS-adjusted measures for their analyses, although at the same time around 60% of the respondents indicated that they have more confidence in IFRS measures. With regards to *Australia*, Harrison and Morton (2010) find that adjusted EPS disclosures represent nearly one-third of all EPS disclosures in annual reports and are disclosed voluntarily by more than one-third of the 94 companies analysed. Moreover, analysis of ASX 500 companies’ full-year profit announcements from 2000 to 2014 by Coulton et al. (2016) suggests that the frequency of non-GAAP reporting experienced a significant upward trend following the implementation of IFRS in Australia in 2005. Regarding *New Zealand*, Rainsbury et al. (2015) document that the proportion of listed companies reporting GAAP-adjusted earnings measures was only 5% in 2004 and 2005, increasing in subsequent years to reach a peak of 20% in 2011 and returning to 16% in 2012. In a recent study, Carvajal et al. (2021) provide empirical evidence for the long-term period 2004–2018, finding that the popularity of NGFMs in annual reports of listed New Zealand companies on the NZX main board increased significantly from 2004 (below 33%) to 2012 (almost 59%), but then declined substantially again until 2018 (under 48%). Besides the United Kingdom and the Australasian region, there are only few articles conducting cross-sectional and time-series analysis of APMs for *other* regions (1.0%), such as Brazil (Alcalde et al. 2013; Vasconcelos de Andrade and Dal-Ri Murcia 2019).

##### (ii) Motives for the Reporting of APMs

The main focus of APM research is on the motives for APM reporting, being investigated by three fifths (38.5%) of the 410 articles. Of these 158 articles, 14.6% examine the potential of *APM reporting as an instrument for investor information*, mostly (11.5%) by analysing *capital market reactions* (i.e., capital market-based regression and correlation studies). Specifically, these studies examine whether investors price the disclosure of (unexpected) APMs into capital market reactions (i.e., *information content* and *value relevance*). The *information content* of APMs is usually analysed by means of event studies, which examine the capital market reaction to a certain new piece of information in a short time window around the time of publication, seeking to isolate causal relationships between APM reporting and capital market reactions (see in detail Hitz 2010a). Early articles analysing the information content of APMs mostly provide evidence that both company-reported APMs (e.g., Bhattacharya et al. 2003; Lougee and Marquardt 2004) and analyst-reported street earnings (e.g., Brown and Sivakumar 2003; Doyle et al. 2003; Gu and Chen 2004) appear to have higher (incremental) information content compared to GAAP earnings. For example, in one of the first studies to analyse both pro forma earnings and street earnings by examining “short-window abnormal returns around earnings announcement dates”, Bhattacharya et al. (2003) find “that pro forma earnings are significantly more informative to investors than GAAP operating earnings” (similarly Lougee and Marquardt 2004). These findings are extended by Bowen et al. (2005), whose overall results suggest that the (relative) form of presentation and emphasis of pro forma earnings figures influences their information content, which is also supported by Allee et al. (2007). This early evidence overwhelmingly suggesting that APMs have higher value relevance compared to GAAP earnings, is also supported by more recent studies. For example, Bradshaw et al. (2018) find evidence that “investors respond more strongly to non-GAAP earnings relative to GAAP earnings” and that they “view non-GAAP earnings as a more informative summary metric of firm performance”.

In contrast to the study of information content, the attribute of *value relevance* merely refers to the association of these variables over longer time periods and leaves open the question of whether the information reflected in share prices was first transmitted to the market via accounting or via other, more timely information channels (Hitz 2010a). The criterion of value relevance thus provides information on whether the capital market prices in the information contained in financial statements but leaves open whether periodic financial reporting is the appropriate vehicle for this provision of information (see in detail Hitz 2010a). Studies analysing the value relevance of APMs provide evidence that both company-reported APMs (e.g., Bradshaw and Sloan 2002; Brown and Sivakumar 2003; Venter et al. 2014) and analyst-reported street earnings (e.g., Entwistle et al. 2010; Wieland et al. 2013) have relatively higher value relevance compared to GAAP earnings. For example, Entwistle et al. (2010) examine the value relevance of pro forma earnings, GAAP earnings and street earnings obtained from *Thomson Reuters’ I/B/E/S *database (I/B/E/S earnings). They find that each of the earnings measures is value relevant, though their “results indicate that pro forma earnings are more value relevant than I/B/E/S earnings, which in turn is more value relevant than GAAP earnings”. More recently, for example, for a sample of Australian ASX 500 companies voluntarily disclosing NGFMs in their full-year profit announcements from 2000 to 2014, Ribeiro et al. (2019), show that “[c]ompared to GAAP earnings, non-GAAP measures are more persistent, more predictable, smoother, and more value relevant”.

Alongside capital market-based studies, 3.2% of the 410 articles examine the potential of APM reporting as an instrument for investor information based on the *predictive ability*, which is an immanent part of the decision-usefulness paradigm of capital market-oriented accounting.^27^ Specifically, these studies address the question of whether APMs are suitable for forecasting the amount and timing of expected future cash surpluses or earnings (Hitz 2010a). This declared objective is taken up in empirical studies by examining the predictive ability of APMs and their components ex post based on time series of reported earnings variables with regard to future cash surpluses or earnings (see in detail Hitz 2010a). In one of the first empirical studies of this kind, Brown and Sivakumar (2003) find that I/B/E/S earnings are more predictive than GAAP earnings (similarly Gu and Chen 2004; Doyle et al. 2003). For company-reported APMs, Lougee and Marquardt (2004) provide first evidence that pro forma earnings have (incremental) predictive ability for pro forma earnings and GAAP earnings, but only when GAAP earnings informativeness is low and there are no incentives for ‘opportunistic’ reporting (proxied by whether GAAP earnings have not declined to the comparative period). More recently, for example, Sinnewe et al. (2017) provide empirical evidence for the period from 2006 to 2011 suggesting that non-IFRS earnings contain important information about future cash flow predictability.

Besides the articles analysing APM reporting as an instrument for investor information, the majority (23.9%) of the 158 articles on APM reporting motives examine *APM reporting as an instrument of adversarial investor influence*. About these articles, a fundamental distinction must be made between studies analysing *adversarial investor influence as motive* and studies investigating *adversarial investor influence as a fact* (similarly Hitz 2010a). Among the 98 articles analysing the motive of adversarial investor influence, the majority (17.6%) analyse potential *adversarial investor influence as motive*, while a minority (6.3%) examine *adversarial investor influence as a fact*. The analysis of *adversarial investor influence as motive* is mainly conducted by analysing *determinants of APM reporting* (15.6%), while only a few articles examine the *predictive ability of earnings components excluded from APMs* (2.0%). The research focus of the 64 articles analysing determinants of APM reporting is to examine company characteristics or framework conditions which show a systematic relationship with management’s decision to voluntarily disclose APMs, as well as the specific design of APMs (see in detail Hitz 2010a).

One of the *first* determinants studied is the achievement of *strategic earnings benchmarks* such as GAAP earnings, analysts’ expectations or break-even (e.g., Ciccone 2002; Lougee and Marquardt 2004; Entwistle et al. 2005). Early evidence for the pre-Reg. G period shows that U.S. companies missing earnings benchmarks such as earnings decreases or consensus analyst forecasts are more likely to report pro forma earnings (Lougee and Marquardt 2004). For the post-Reg. G period, Yi (2012) finds that NGFM disclosures by companies with historically low return-GAAP earnings ratios are more pronounced than in the pre-Reg. G period. A *second* intensively studied determinant is *management incentives* such as (variable) management compensation (e.g., Bansal et al. 2013; Grey et al. 2013; Isidro and Marques 2013; Islam et al. 2019). For example, Bansal et al. (2013) find evidence that managers with higher risk incentives through stock-based compensation arrangements are more likely to voluntarily disclose NGFMs. Islam et al. (2019) find that all three components of CEO remuneration (i.e., base, short-term and long-term incentives) have a significant impact on the decision to report NGFM in profit announcements. More recently, Black et al. (2021b) found a negative association between long-term incentive plan pay-outs and the likelihood and magnitude of aggressive NGFM exclusions, indicating that managers tend to report NGFM information less aggressively when they focus more on long-term rather than short-term value.

A *third* determinant analysed is *industry affiliation*, the analysis of which suggests that APM disclosures cluster in certain industries and companies with specific characteristics (similarly Coulton et al. 2016). For example, in a recent study, Isidro and Marques (2021) examine the influence of industry-level product market competition using traditional measures of industry competition (concentration, price-cost margin and set up costs) on management’s NGFM disclosure decisions. They find that the intensity of industry competition affects the likelihood of NGFM and the extent of non-GAAP exclusions, but overall their findings suggest that industry competition has a positive impact on non-GAAP disclosure transparency. Taken together, the studies examining industry affiliation as a determinant suggest that APM reporters tend to be young companies and companies which are concentrated in the tech, high-tech and business services sector (e.g., Bhattacharya et al. 2003; Zhang and Zheng 2011). A *fourth* determinant analysed intensively, is *corporate governance mechanisms*. Studies on this determinant seem to support the proposition that companies with weak corporate governance mechanisms (e.g., less board independence) are more likely to optimistically report APMs, suggesting that strong corporate governance protects investors from misleading APM disclosures (e.g., Jennings and Marques 2011; Seetharaman et al. 2014). For example, in a recent study, Lee (2021) finds evidence that companies where the chair of the independent audit committee holds more external directorships have stronger monitoring and thus provide higher quality NGFM disclosures.

Besides these four determinants, which have already been studied more intensively, a particular focus in the APM literature is also placed on the following determinants:

- the *accounting standards* applied such as IFRS accounting in general (Sek and Taylor 2011; Solsma and Wilder 2015; Shibasaki and Toyokura 2020) as well as fair value measurement (Malone et al. 2016) and accounting reporting complexity (Brown et al. 2020) in particular;
- *countries’ institutional, economic* and *cultural factors* (Isidro and Marques 2015; Visani et al. 2020; Bini et al. 2020);
- *equity valuation* (Badertscher 2011; Hsieh 2010);
- *company-size* (Bouwens et al. 2019; Bini et al. 2020) and *other company-specific attributes* (Charitou et al. 2018; D’Angelo et al. 2018);
- *rounding phenomenon* (He 2018; Wu et al. 2012);
- *media attention* (Koning et al. 2010);
- *investor sentiment* (Brown et al. 2012b);
- *managerial reputation* (Cheng 2017) and *sentiment* (Chen 2019);
- *debt covenant violations* (Christensen et al. 2019);
- *CEO turnover* (Jarva et al. 2019);
- *analyst coverage* (Christensen et al. 2021); and
- *the CEO’s personal narcissism* (Abdel-Meguid et al. 2021).

In contrast to articles analysing determinants of APM reporting, eight articles (2.0%) examine the *predictive ability of earnings components excluded from APMs*. Specifically, these articles address the question of whether components excluded from GAAP earnings due to their supposedly non-recurring, unusual or non-operational nature do in fact not unfold predictive ability for future cash flows or earnings (Hitz 2010a). In one of the first studies, Doyle et al. (2003) find that earnings components excluded from I/B/E/S earnings have lower but still significant and substantial predictive ability for future cash flows (similarly Kolev et al. 2008). Similarly, Yi (2012) finds “that the negative association between income-increasing non-GAAP adjustment and abnormal returns exists in the pre-Reg. G period”. However, the results also show that for the post-Reg. G period, this tendency is no longer evident (Yi 2012). Overall, the empirical findings on the predictive ability of earnings components excluded from APMs seem to indicate that companies tend to exclude both transitory as well as recurring items (Coulton et al. 2016). On the one hand, these findings can be attributed to management’s lack of competence in adjusting earnings, but on the other hand, it can also be reconciled with the proposition of strategically motivated adversarial investor influence, especially since the excluded components have negative explanatory power for future cash flows (similarly Hitz 2010a).

The investigation of the motive of *adversarial investor influence as a fact* is examined in 6.3% of the 410 articles. Of these 26 articles, about half are capital market-based studies (3.4%). Such studies generally do not question the findings of capital market-based studies analysing the information content or value relevance of APMs, but rather examine whether investor reactions to APM disclosures are appropriate given the information content of voluntarily disclosed APMs. Specifically, based on the aforementioned findings suggesting that earnings components excluded from APMs have significant predictive ability for future cash flows or earnings, these studies examine the extent to which this fact is reflected in capital market prices (see in detail Hitz 2010a). For example, Landsman et al. (2007) find significant negative incremental explanatory power of street earnings exclusions through regression of stock market value, suggesting that investors do not fully recognise negative predictive ability (similarly Doyle et al. 2003). In addition, several studies examine systematic variations in investor reactions to APM reporting as determinants of differentiated capital market reactions. These studies can be distinguished on the company side into management incentives to strategically use APM reporting and on the investor side into the perception of APMs by professional (i.e., institutional) investors compared to non-professional (i.e., retail) investors (Hitz 2010a). Studies on management incentives to strategically use APM reporting suggest that investors assess the trustworthiness of APMs differentially regarding potentially strategic reporting motives of managers. For example, on the one hand, Brown et al. (2012a) find that the strategic character of earnings announcement timing is recognised by investors, while on the other hand Hsu and Kross (2011) provide an indication that investors do not fully understand that APMs may be aggressively framed through the inclusion or exclusion of special items. Capital market-based studies on the perception of APMs by different investor groups, such as professional (i.e., institutional) investors and non-professional (i.e., retail) investors, provide further insights on the influencing potential of APMs. For example, Allee et al. (2007) find that less-sophisticated investors trade incrementally more, while more-sophisticated investors actually trade less (or in the opposite direction) when a pro forma earnings number is present. Similar evidence suggesting that non-sophisticated investors tend to react more strongly to APMs is also provided, amongst others, by Bhattacharya et al. (2007) and Jennings and Marques (2011). Accordingly, if one assumes that professional investors deliberately do not trade around the publication date of APM announcements due to their expertise, the empirical findings provide an indication of a misjudgement of APMs by non-professional investors and thus document a possible potential to influence (similarly Hitz 2010a). Should aggressive APM disclosures be practised by management primarily for self-serving reasons, an adverse influence on non-professional investors could not be ruled out. Therefore, the perception of APMs by professional investors compared to non-professional investors is also examined in detail in experimental studies.

Experiments investigating the motive of *adversarial investor influence as a fact *are conducted in twelve articles (2.9%). Most of these *experimental studies* investigate the influence of APM reporting on different investor groups by distinguishing between professional investors (mostly represented by financial analysts) and non-professional investors (mostly represented by M.B.A. students). Overall, the experimental results support the empirical evidence that not only the mere presence of APMs (Frederickson and Miller 2004), but also the emphasis (i.e., the graphical display) that management places on APMs (Elliott 2006; Dilla et al. 2013; Johnson et al. 2014) influences the judgements and decisions of non-professional investors in particular. As the experimental evidence shows, non-professional investors tend to overreact to the presence of APMs, especially when the corresponding GAAP earnings are a loss (Andersson and Hellman 2007) or managers emphasise APMs. In contrast, professional investors seem to understand that management strategically uses APM disclosures (e.g., Reimsbach 2014). Moreover, previous experimental studies provide evidence that APMs influence non-professional investors’ valuations through unintended cognitive effects, as their information processing is influenced by the presence of APMs (Frederickson and Miller 2004) or their emphasis (Elliott 2006). Also, experimental evidence suggests that non-professional investors should not be understood as a homogeneous group, but that the relative level of financial reporting knowledge and information viewing behaviour have a significant impact on their judgements and decisions (Dilla et al. 2014). In contrast to non-professional investors, professional investors’ decisions do not appear to be significantly influenced by the presence or emphasis of APMs. However, professional investors seem to consider APMs more reliable when a GAAP earnings reconciliation is present (Frederickson and Miller 2004).

##### (iii) Regulation of APM Reporting

About one-tenth (11.2%) of the 410 articles deal with the regulation of APM reporting, with the vast majority of 8.5% analysing the regulatory activities in the U.S. Typically, these studies use the issuance of Reg. G in 2003 and/or the issuance of the C&DIs in 2010 to examine how usage (i.e., *frequency*), *presentation, quality of exclusions* and *market perception* of NGFMs have changed in response to the regulatory changes. Overall, it can be concluded that Reg. G has led to a decline in (i) the *frequency* of NGFM reporting, (ii) the relative proportion of NGFMs exceeding GAAP earnings and (iii) the average size of non-GAAP exclusions. For example, Entwistle et al. (2006b) find that following the introduction of Reg. G the proportion of S&P 500 companies reporting pro forma earnings in annual earnings press releases declined by about 30% from 77% in 2001 to 54% in 2003 (similarly Nichols et al. 2005; Marques 2006; Heflin and Hsu 2008). However, this decline in the *frequency* of NGFM reporting by listed U.S. companies was rather short-term in nature and increased again in subsequent years after Reg. G came into force, at the latest from the liberalisation of Reg. G rules by the C&DIs in 2010 onwards. For example, Henry et al. (2020a) document that following the C&DIs in 2010, the proportion of S&P 100 companies disclosing NGFMs increased significantly, especially from 2010 (54%) to 2011 (66%) and from 2011 to 2012 (71%), before settling at a stable level between 72% and 75% from 2013 to 2016.

In terms of *presentation* of NGFMs, a significant reduction in the (relative) emphasis of NGFMs compared to GAAP earnings and a decrease in the use of potentially misleading non-GAAP terminology can be observed. For example, Entwistle et al. (2006b) find that under Reg. G by 2003 there are 44% fewer companies reporting NGFMs in the headline and 77% fewer companies discussing NGFMs in a dominant manner in the full press release. Lastly, the regulatory interventions are also associated with higher *quality of exclusions*, improved *market perception* and appear to have led to more accurate pricing of securities, particularly due to the requirement for reconciliation between NGFMs and GAAP earnings by Reg. G. For example, Black et al. (2017a) provide evidence that after Reg. G came into force “managers generally exclude fewer recurring items upon which analysts disagree in the post-SOX regulatory period and that managers are also less likely to make recurring exclusions to meet strategic earnings targets” (similarly Heflin and Hsu 2008; Shiah-Hou and Teng 2016). Moreover, Yi (2012) documents that incremental information content of NGFM has increased after Reg. G, but the negative associations between earnings increasing non-GAAP exclusions and future returns have decreased, mainly due to companies that stopped disclosing NGFMs after the SEC intervention.

In *Europe*, legislators have also responded to the increasing extent and frequency of APM reporting. So far, the regulatory activities in Europe are analysed in ten articles (2.4%), providing similar results as for U.S. regulation. For example, Magli et al. (2017) conduct a survey of employees in investor relations departments of Italian listed industrial companies regarding the impact that companies expect from the first-time application of the ESMA guidelines in 2016. They find that “[t]he entities do not expect significant changes because, in most cases (98%), the entities that use APMs already reconcile these with financial statements figures”. Also before the ESMA guidelines came into force, Ruhwedel et al. (2017) analysed the 2015 annual reports of 72 non-financial DAX and MDAX companies with regard to their need for adoption to the ESMA guidelines. In contrast to the results of the survey of Italian companies by Magli et al. (2017), based on their descriptive evidence, Ruhwedel et al. (2017) conclude that the ESMA guidelines imply a need for adoption for many DAX and MDAX companies in their annual reports. In a follow-up study, Ruhwedel et al. (2018) examine the effects of the first-time adoption of the ESMA guidelines for financial years 2014 to 2016, also in annual reports of DAX and MDAX companies. Their descriptive evidence shows that the disclosure behaviour of companies has changed significantly following the implementation of the ESMA guidelines, which is expressed in (i) a decline in the number of disclosed APMs, (ii) an improved level of transparency and (iii) a reduced extent of earnings modifications (Ruhwedel et al. 2018). Further evidence on the impact of the ESMA guidelines on European companies is provided by Vinciguerra et al. (2020), who find a slight decrease in the number of companies reporting APMs. Moreover, they find that positive APM adjustments of expenses prevail when reconciling APMs to GAAP earnings, which Vinciguerra et al. (2020) cautiously interpret as an indication that companies use APMs to communicate an improved earnings performance compared to GAAP earnings performance.

Apart from the findings for the U.S. and Europe, there is only one article on the regulation of APM reporting in *Australasia*, in which Rainsbury (2017) examines the effects of the *Guidance Note*: *Disclosing Non-GAAP Financial Information *(FMA guidance note) introduced by the *New Zealand Financial Markets Authority* (FMA) in 2012. She finds evidence that, following the introduction of the FMA guidance note, “companies reporting non-GAAP information are complying more with the disclosure criteria and giving more emphasis to reporting GAAP earnings compared with non-GAAP earnings” (Rainsbury 2017). Besides the aforementioned cross-industry regulations, Baik et al. (2008) and Fortin et al. (2008) examine the impact of regulatory measures in the real estate investment trust (REIT) industry in the U.S., where the reporting of sector-specific APMs, such as ‘funds from operations’ (FFO), had existed for more than a decade in a rules-based self-regulatory environment (see in detail Coulton et al. 2016).

##### (iv) Other

The remaining 158 articles (38.5%) are predominantly articles that do *not mention a specific research focus*. Essentially, these are 135 articles (32.9%) without any indication of a specific research focus. In particular, these are articles in practitioner and trade magazines (13.9%), commentaries and viewpoints (7.6%), discussion and review articles (4.1%) and contributions to collective works (2.2%) not applying a scientific research methodology (see Fig. 3). The remaining 23 articles (5.6%) in the *other* category deal with research topics that do not fit into the categories shown in Fig. 3. Particularly, these include studies analysing the impact of APMs on investment decisions in general (e.g., Laurion 2020; Wong and Wong 2010; Fridson 1998), the relationship between earnings management and APMs (e.g., Black et al. 2017b; Elshafie et al. 2010) and differences between APMs and street earnings (e.g., Bentley et al. 2018; Batta and Muslu 2017).

Within our *scenario analysis*, when looking at the subsample that includes only academic articles, we find a substantial shift in the focus of APM research towards the analysis of the *motives for APM reporting* (+ 15.0%) and a moderate shift towards the *regulation of APM reporting* (+ 2.8%; see Table 1). In turn, the proportion of articles that do *not mention a specific research focus* (particularly practitioner and trade magazines, commentaries and viewpoints, discussion and review articles) decreases substantially (− 15.8%), and the proportion of articles that conduct *cross-sectional and time series analyses of APMs* also decreases, but only moderately (− 3.4%). A similar shift in the focus of APM research towards the analysis of the *motives for APM reporting *(+ 5.3%) also emerges when looking at the subsample that includes only international articles, although this shift would be much more moderate compared to the subsample including only academic articles. These shifts in the research focus when looking at the two subsamples seem quite plausible insofar as both the articles written in German as well as the professional articles included in our sample are predominantly theoretical in nature (i.e., commentaries, viewpoints, discussion and review articles).

*In summary*, from the classification of articles according to the research focus, we can learn that the focus of APM research to date has been on the motives for APM reporting, cross-sectional and time series analyses of APMs and the regulation of APM reporting. *Cross-sectional and time series analyses of APMs* are mainly conducted in the early stages of APM research for the North American region (and here particularly the U.S.). Specifically, these articles address questions of prevalence, quantitative characteristics and the evolution of APMs over time. However, the main focus of APM research is on the *motives for the reporting of APMs*. Here, most of the articles analyse APM reporting as an instrument for investor information particularly based on the analysis of capital market reactions, while APM reporting as an instrument of adversarial investor influence is primarily examined by analysing the determinants of APM reporting. A third research focus is the analysis of *regulatory impacts on APM reporting*, but again this focus is on the North American region (especially the U.S.) and only a few studies are available for the European and Australasian region.

#### Research Method

**Fig. 4:**
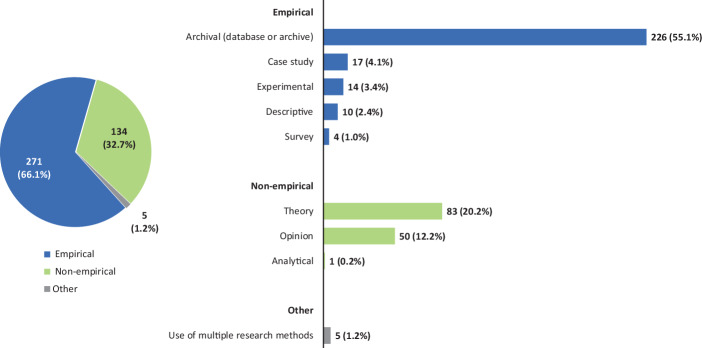
Research methods of the articles on APMs

Based on the methodology according to the *European Accounting Association *(EAA 2019), the research methods applied in the 410 articles can be divided into three main categories: *empirical, non-empirical* and *other*, which in turn can be further divided into subcategories. As Fig. 4 shows, *empirical *methods are by far the most frequently used research approaches, applied in two thirds (66.1%) of all 410 articles analysed. Of these 271 empirical studies, the vast majority of more than half (55.1%) of all 410 articles apply *archival (database or archive)* research. In these 226 articles, sophisticated statistical methods are used to infer relationships in the population (also referred to as ‘inductive statistics’ or ‘inferential statistics’) (Smith 2017). Specifically, these articles apply methods that test hypotheses about relations between variables in the population (methods of statistical hypothesis testing) and/or estimate variables in the population (methods of statistical parameter estimation) to data that has either been collected manually (i.e., by hand), particularly by document analysis of company publications (e.g., annual reports, earnings announcements or press releases) or machine-collected data obtained from commercial databases (e.g., analyst or company databases; see in detail Sect. 4.2.5).

The second most prevalent but much less frequently used empirical research method, applied in 17 articles (4.1%), is *case study *research, which is concerned with examining the role and tasks of accounting in its natural context (Smith 2017). For example, Aselta and Engel (2018) develop a teaching case for understanding the impact of NGFMs and related SEC regulations on the nature, derivation, usage and regulation of NGFMs. Moreover, 14 articles (3.4%) apply *experimental *methods. Experiments have the advantage over other empirical methods that the influence of the variation of individual variables or characteristics can be examined in isolation and thus controlled for other influencing factors (Döring and Bortz 2016). These experiments on APMs address the motive of adverse investor influence as a fact, in particular the perception of APMs and related (investment) decisions of professional and non-professional investors (see in detail Sect. 4.2.1). Another empirical research method applied in ten articles (2.4%) is the *descriptive*-statistical analysis of primary data which has been collected manually (i.e., hand-collected) through the systematic analysis of information disclosed in corporate publications (e.g., earnings announcements, press releases, quarterly and annual financial statements). In these ten articles, different descriptive methods such as tables, charts or statistical parameters (e.g., proportions, frequencies and measures of location and dispersion) are used to organise, summarise and present the sample data (Blasius and Baur 2019). In contrast to archival (database or archive) research methods, however, the findings obtained in these articles applying descriptive-statistical methods do not allow for generalisation, but (merely) for a description of certain characteristics in the sample and thus rather serve as a preliminary stage for hypothesis testing and theory building (Blasius and Baur 2019; Häder 2015).

Of all empirical methods, *survey* research has so far been used least frequently, in only four articles (1.0%). Generally, in this method, researchers ask a (usually randomly selected) sample of people about their background, past experiences or behaviour through questionnaires or interviews (Frankfort-Nachmias et al. 2015). Specifically, of the articles using survey research, three studies use questionnaires for data collection. For example, Magli et al. (2017) use questionnaires sent to the investor relations departments of Italian listed industrial companies to evaluate the expected impact of the first-time application of the ESMA guidelines. Papa et al. (2016) use questionnaires to survey members of the *CFA Institute*, including portfolio managers, buy-side and sell-side analysts. Moreover, the CFA Society (2015) combines analysis of quantitative data taken from *S&P Capital IQ *with survey data of 292 CFA UK members. Interviews have so far only been used in one article applying survey research, in which (expert) interviews with corporate finance specialists (2) and sell-side financial analysts (8) were conducted to collect data (Fülbier et al. 2021). However, interviews are used in combination with other research methods in three other articles (*use of multiple research methods*). For example, Leibfried et al. (2018) combine quantitative data analyses with semi-structured interviews of accounting heads (4) and financial analysts (2). Similarly, Davern et al. (2018) combine quantitative data analysis with semi-structured interviews of investors (7), regulators (5) and practitioners (5). Also, Davern et al. (2019) combine archival methods with field interviews of investors (7), regulators (5) and auditors (5).

*Non-empirical *methods are used in a third of the 410 articles analysed (32.7%). Most of these articles (20.2%) are *theoretical* in nature and discuss the purpose, definitions and/or trends of APM reporting (e.g., Pizzo 2020; Sherman and Young 2018; Papa 2017; Ciesielski and Henry 2017) or regulatory implications (e.g., Adams and Meckfessel 2021; Di Fabio and Roncagliolo 2020; Parrino 2019), as far as possible, from an objective perspective. These theoretical articles also include seven literature reviews (e.g., Arena et al. 2021; Catuogno and Arena 2020; Marques 2017). In contrast to these (rather) objective theoretical articles, however, about one tenth (12.2%) of the analysed sample are articles in which the authors present their subjective *opinion*, for example on theoretical research aspects (e.g., discussion and review articles) or practical aspects of APM reporting, such as current regulatory initiatives by ESMA or IASB (e.g., commentaries and viewpoints). Most of these articles are commentaries/viewpoints (8.3%) and a minority are discussion and review articles (4.1%). Besides objective and subjective theoretical articles, *analytical* methods have so far only been used in one article (0.2%). In this study, Hirshleifer and Teoh (2003) derive their conclusions through an explicit analysis of a mathematical model that reflects both legitimate reasons for APM reporting and the possibility of manipulating APM disclosures to exploit limited investor or analyst attention. Interestingly, this analytical study by Hirshleifer and Teoh (2003) has both the highest CPYs and CIs (see Tables 2 and 3). The remaining five articles (1.2%) in the *other* category apply *multiple research methods*. For example, Vinciguerra et al. (2020) use a mixed method design, combining quantitative and qualitative methods to analyse the impact of the ESMA guidelines on APM reporting behaviour of European companies. Moreover, three articles combine quantitative data analyses with interviews (Davern et al. 2019, 2018; Leibfried et al. 2018) and one article combines analysis of quantitative with survey data (CFA Society 2015).

*In summary*, from the classification of the articles according to the research method, we can learn that there is a high concentration on *archival (database or archive)* research (55.1%), whereas other empirical methods are only used in about one tenth (11.0%) of the 410 articles analysed. Moreover, the results show a concentration on *theoretical* (20.2%) and *opinion* articles (12.2%), which is mainly due to the non-academic literature included in our sample. Thus, about two-thirds of the theoretical articles (69.9%) and opinion articles (64.0%) are professional articles. Accordingly, when looking at the subsample that includes only academic articles within our *scenario analysis*, there is a substantial shift in the focus of APM research from *non-empirical* methods, namely *theoretical* articles (− 9.3%) and *opinion* articles (− 4.3%), to empirical methods, especially *archival* studies (+ 14.6%) and to a much lesser extent to *experimental* studies (+ 1.8%). However, when looking at the subsample that includes only international articles, this shift in the focus of APM research is much more moderate. Specifically, for this subsample, there is a shift towards *archival* studies (+ 3.0%) and, to a lesser extent, towards *opinion* articles (+ 1.2%). In turn, the subsample of international articles contains lower proportions of *case studies* (− 1.6%), *theoretical *articles (− 1.5%) and *descriptive* studies (− 1.3%) compared to the total sample. These shifts in the research focus when looking at the two subsamples seem plausible insofar as the research focus in professional journals, especially in professional articles written in German, tends to be on *theoretical* methods (i.e., *theoretical* and *opinion* articles) and rather less sophisticated empirical methods, including *case studies* and *descriptive *analysis.

#### Research Location and Accounting Regimes

**Fig. 5:**
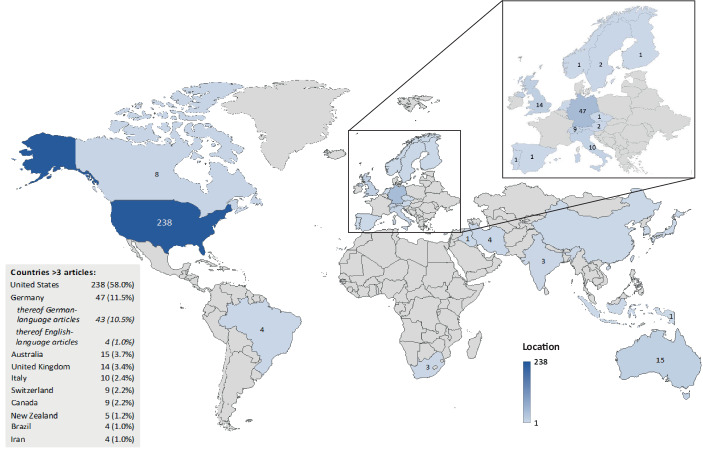
Research location of the articles on APMs

The results for the research location show that *North America* is by far the most frequently analysed region, representing three-fifths (60.2%) of all 410 articles (see Table 1). Almost all of these 247 articles deal with an analysis of NGFM reporting in the U.S. (58.0%) and only a handful in Canada (2.2%) (see Fig. 5). The second most analysed region is *Europe* (including Norway, Finland and Sweden), accounting for more than two-fifths (22.2%) of all articles analysed, including Germany, which is the European country with most articles (11.5%). This, however, is mainly due to the inclusion of the articles written in German in our sample. Of all 47 articles analysing APMs reported by German companies, 43 articles (10.5%) are written in German. These 43 German-language articles are almost exclusively professional articles. These findings are consistent with previous evidence, suggesting that non-English-speaking researchers tend to publish their ‘best’ results in international journals (Ellis 2010). Accordingly, when looking at the subsample that includes only international articles within our *scenario analysis*, there is a substantial shift in the focus of the analysed literature from *Europe*, particularly *Germany* (− 11.0%), to *North America* (+ 8.8%) and, to a lesser extent, the *Australasian* region (+ 1.5%). A similar shift in the focus of APM research from *Europe*, particularly *Germany* (− 12.1%), to *North America* (+ 6.4%) and *Australasia* (+ 5.4%) also emerges when looking at the subsample including only academic articles. Besides Germany, the two European countries *Italy* (2.4%) and *Switzerland* (2.2%) are also examined more intensively. However, eight out of the ten articles analysing APMs in an Italian context are contributions from a single collective work (Moscariello and Pizzo 2020). Following Europe, the third most studied region is *Australasia*, representing one tenth (10.0%) of the 410 articles analysed. Most of these 41 articles focus on APM reporting in *Australia* (3.7%), *New Zealand* (1.2%), *Iran* (1.0%) and *Hong Kong* (0.7%). The least researched regions so far are the *United Kingdom* (3.4%) and countries in the *other* category (4.1%) such as Brazil (1.0%) and South Africa (0.7%).

**Fig. 6:**
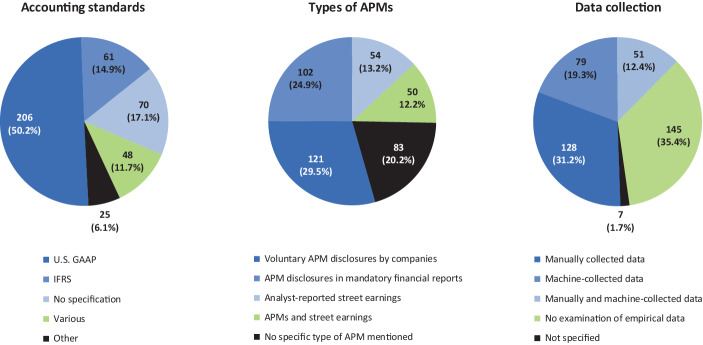
Accounting standards, types of APMs and data collection of the articles on APMs

Like the research location, the accounting regimes analysed in the 410 articles show a concentration on the North American region. About half (50.2%) analyse NGFMs in light of *U.S. GAAP* (see Fig. 6). Less than one sixth (14.9%) of the sample examines APMs in the context of *IFRS*. Besides U.S. GAAP and IFRS, another 11.7% deal with *various accounting regimes*. In particular, these include studies analysing APM reporting in the period(s) before and after the first-time application of IFRS in the respective countries (e.g., Isidro and Marques 2013; Venter et al. 2014; Lont et al. 2020). Furthermore, 6.1% analyse APM reporting regarding *other*, usually national, accounting regimes, particularly *Australian Accounting Standards* (AAS; 2.7%), *Generally Accepted Accounting Practice in the UK* (UK GAAP; 1.0%), *New Zealand Generally Accepted Accounting Practice *(NZ GAAP; 0.7%) and the *German Commercial Code* (HGB; 0.5%). However, 17.1% of the articles do *not specify *whether they focus on a specific accounting regime. Within our *scenario analysis* regarding the accounting regimes analysed, we observe a shift of the research focus from *IFRS* to *U.S. GAAP*, when looking at the subsamples that include only academic articles or only international articles. In addition, there would be slight shift from articles analysing *various* accounting regimes to *other*, usually national, accounting regimes.

*In summary*, from the classification of the articles according to the research location and the accounting regimes we can learn that there is a clear research focus on the internationally relevant accounting regimes U.S. GAAP and IFRS. The tendency of previous APM research to focus on U.S GAAP data becomes even clearer when looking at the two subsamples that include only academic articles or only international articles.

#### Types of APMs

The 410 articles analysed use different types of APMs and/or methods to approximate APMs. In this context, two fundamentally different types of earnings measures need to be distinguished: APMs reported by companies (i.e., management) on the one hand and street earnings reported by analysts on the other (see in detail Sect. 2.1). With respect to the analysed sample, more than half of all 410 articles (54.4%) analyse company-reported APMs (i.e., GAAP earnings adjusted by management). Of these 223 articles, about half (29.5%) analyse unaudited *voluntary APM disclosures by companies* (e.g., in earnings announcements or press releases) and the other half (24.9%) analyse *APM disclosures in mandatory financial reports* (e.g., financial statements, especially the management discussion and analysis; see Fig. 6). Regarding the specific types of APMs in these 223 articles analysing company-reported APMs, about half (49.3%) examine several different APMs, while 28.3% focus on EB measures such as EBIT (e.g., Wong and Wong 2010; Höllerschmid et al. 2005) or EBITDA (e.g., Cormier et al. 2017; Bouwens et al. 2019). Besides these, the most frequently analysed company-reported APMs are adjusted EPS measures (9.4%; e.g., ‘non-GAAP EPS’), earnings exclusions (6.7%; e.g., ‘non-GAAP exclusions’), funds from operations (FFO; 2.7%) and adjusted net income measures (1.8%; e.g., ‘non-GAAP earnings’).

In addition to company-reported APMs, more than one-tenth of the articles (13.2%) analyse *analyst-reported street earnings*. In these articles, ‘street EPS’ (7.1%) (i.e., analyst-adjusted EPS) and ‘street adjustments/exclusions’ (4.1%) (i.e., earnings components excluded by analysts) are the most commonly used street earnings figures. Such analyst-reported street earnings were initially used as a proxy for management-reported APMs (e.g., Bradshaw and Sloan 2002; Doyle et al. 2003; Gu and Chen 2004). Accordingly, APMs and street earnings were initially considered as one category of earnings measures and sometimes jointly referred to as APMs (similarly Marques 2017; Bradshaw and Sloan 2002). For example, when analysing the motives, earnings components excluded by analysts were used to examine whether they are suitable for forecasting future earnings (e.g., Doyle et al. 2003; Kolev et al. 2008; Landsman et al. 2007). Reasons for approximating company-reported APMs by using analyst-reported street earnings are that street earnings are an easy-to-access, machine-readable dataset provided by analyst databases (e.g., *Thomson Reuters’ I/B/E/S* or *First Call Research Data*) in a timely and cost-saving manner, allowing for the analysis of large samples (similarly Abdel-Meguid et al. 2021; Hitz 2010a). Moreover, the definition and determination of analyst-reported street earnings is often more consistent over time compared to company-reported APMs (Landsman et al. 2007). However, the use of street earnings as proxy for company-reported APMs may lead to potentially biased performance measures and therefore risks systematically underestimating the variation of APMs. This for example, because analysts reject exclusions if they believe the exclusions are strategically motivated and do not serve the purpose of informing investors (similarly Abdel-Meguid et al. 2021). Moreover, Bentley et al. (2018) provide evidence that street earnings sometimes differ from GAAP earnings when managers do not make any exclusions at all, implying that street earnings do not (always) represent the exclusions made by managers. Additionally, analysts are subject to specific incentives and pursue individual motives when determining street earnings. For these reasons, we believe that street earnings should be considered as a separate, independent category of performance measures (similarly Hitz 2010a). Consequently, most recent studies meanwhile explicitly distinguish between company-reported APMs and analyst-reported street earnings in a way, so that street earnings are no longer used as a proxy for company-reported APMs (similarly Marques 2017). However, besides these articles that exclusively analyse either company-reported APMs or analyst-reported street earnings, about one tenth (12.2%) of the sample analyses both categories of earnings measures (i.e., *APMs and street earnings*) in combination. The remaining 20.2% of the 410 articles deal with APMs in general *without mentioning a specific type of APM*.

*In summary*, from the classification of the articles according to the types of APMs, we can learn that a major focus of previous research has been on the analysis of company-reported APMs (54.4%). In this context, a distinction is to be made between unaudited *voluntary APM disclosures by companies* on the one hand and *APM disclosures in mandatory financial reports* on the other. Besides company-reported APMs, some studies, especially earlier ones, use analyst-reported street earnings to approximate company-reported APMs or analyse both categories of earnings measures (i.e., company-reported APMs and analyst-reported street earnings). A somewhat more differentiated picture emerges within our *scenario analysis *when looking at the subsamples that include only academic articles and only international articles respectively. If we include only academic articles in our analysis, there is a shift in the research focus from *APM disclosures in mandatory financial reports* (− 7.8%) and articles dealing with APMs in general *without mentioning a specific type of APM* (− 10.6%) to *voluntary APM disclosures by companies* (+ 7.3%) and a more frequent use of *analyst-reported street earnings* in the analysis. This is mainly because the non-academic articles included in our sample are, on the one hand, theoretical and opinion articles not referring to a specific type of APM and, on the other hand, descriptive articles conducting cross-sectional and time-series analyses of APMs. In contrast, sophisticated archival studies regularly found in academic journals more often analyse unaudited voluntary APM disclosures by companies and analyst-reported street earnings, since such studies examine larger samples and also regularly use databases to collect data. A similar shift in the focus of the APM literature analysed emerges when looking at the subsample that includes only international articles (see Table 1).

#### Data Collection

The use of different types of APMs is accompanied by different methods of data collection. With regard to the analysed sample, about two thirds (64.6%) of the 410 articles analyse APMs using empirical data (see Fig. 6). In these 265 articles, in 31.2% the empirical data are obtained by manual (i.e., hand) collection of data (*manually collected data*). Specifically, in these articles the APMs analysed are mainly obtained through document analysis of corporate publications such as earnings announcements or press releases (13.2%) and mandatory annual reports (11.0%). Such data, collected directly from company publications, is intended to more accurately capture management-adjusted APMs. However, manual data collection of APMs is typically both costly as well as time-consuming and also carries the risk of discretionary data collection and analysis (similarly Hitz 2010a). About one fifth (19.3%) of the 410 articles analysed use data obtained through database queries (*machine-collected data*). The databases used here are, on the one hand, analyst databases (6.8%), in particular *Thomson Reuters’ I/B/E/S* (6.1%). On the other hand, databases in which financial (key) figures from corporate publications are made available on a large scale and databases with which corporate publications of a certain sample (e.g., via a selection index) can be systematically searched are used to obtain empirical data (5.4%). The most commonly used databases for this purpose are *directEDGAR, LexisNexis *and *CRSP/Compustat Merged Database*. Differences arise here depending on the databases used and the search terms as well as the evaluation level (i.e., whether only certain parts or the entire documents are searched for APMs) (similarly Hitz 2010a). For example, Bhattacharya et al. (2007) search for APMs throughout the whole press releases, while Johnson and Schwartz (2005) search for APMs only in the headlines and first paragraphs. A combination of database queries in company and analyst databases to obtain empirical data is carried out in 7.1% of the 410 articles analysed. In contrast, 12.4% of the 410 articles analysed combine manually collected data (especially through document analysis of company publications) with machine-collected data from analyst and company databases (*manually collected and machine-collected data*). However, seven articles (1.7%) do *not specify* how the empirical data analysed were obtained. The majority of the 145 articles (35.4%) *not examining empirical data* are quite predominantly those in which non-empirical (i.e., theoretical) methods are used (see in detail Sect. 4.2.2).

*In summary*, from the classification of the articles according to the method of data collection, we can learn that both manual data collection as well as database queries play a significant role in APM research so far. With regard to manually collected data, the data has so far mainly been obtained through the analysis of company publications (i.e., hand collected), while in terms of databases, analyst databases play an important role (especially *Thomson Reuters’ I/B/E/S*). However, a somewhat more differentiated picture emerges within our *scenario analysis *when looking at the subsamples that include only academic articles and only international articles respectively. If we include only academic articles in our analysis, there is a shift in the focus of previous APM research from articles *not examining empirical data* (− 13.0%) to articles analysing *machine-collected data* (+ 7.0%) and articles examining both *manually collected as well as machine-collected data *(+ 5.5%). This is mainly because articles published in non-academic journals are predominantly theoretical and opinion articles not analysing empirical data. In contrast, sophisticated archival studies regularly published in academic journals usually use larger samples and also make more frequent use of machine-collected data. A similar shift in the focus of the APM literature emerges when looking at the subsample that includes only international articles (see Table 1).

## Discussion of Future Research Paths

In this Sect. 5, we identify future research paths to answer RQ3 (*What is the future for APM research?*). To answer this question, we draw on the findings presented above in order to facilitate a decided derivation of research gaps based on our reliable and valid analytical framework.

### *Implication 1:* Developing a Research Synthesis

Our results show a high concentration of the 410 articles analysed on three research topics. So far, the focus of APM research is essentially on (i) motives for APM reporting (38.5%), (ii) cross-sectional and time series analyses of reported APMs (11.7%) and (iii) regulation of APM reporting (11.2%), indicating the need for a research synthesis (Denyer and Tranfield 2006). So far, there is no research that attempts to synthesise the results of several studies in a systematic manner. According to Stanley (2001), research synthesis is an effective tool for “searching through mountains of potentially contradictory research to uncover the nuggets of knowledge that lie buried underneath”. Depending on the epistemological position, different approaches can be used to synthesise research results, such as meta-analyses or co-citation analyses, which all have the objective in common to summarise the results of a large number of empirical studies in a coherent manner (Tranfield et al. 2003).

### *Implication 2:* Studying New Research Topics

Besides research synthesis, we suggest scholars to focus on APM research topics which, to the best of our knowledge, have not yet been researched or have been researched only little.

#### a) Regulation of APM Reporting in Europe

More research should be conducted on the impact of the ESMA guidelines on APM reporting by European listed companies (similarly Marques 2017; Magli et al. 2017). Compared to the empirical evidence on the regulatory activities in the U.S., to date there is scarce empirical evidence on the intended (and the potentially unintended) regulatory consequences of the ESMA guidelines. Hitherto, the conducted empirical studies on the practical impact of ESMA guidelines are almost exclusively descriptive in nature (e.g., Ruhwedel et al. 2018). For these reasons, we call for archival (i.e., explanatory) research to better assess the usefulness and economic consequences of the regulatory measures at the European level. In this context, it seems promising to replicate previous methodologies from U.S. studies to draw comparisons between the regulatory impact in the U.S. and in Europe. Such comparative studies could be of interest to scholars but also to practitioners, because comparisons between Reg. G and ESMA guidelines could reveal insights that contribute to the mutual improvement of both regulatory frameworks. Also, when analysing the impact of the ESMA guidelines, scholars should consider including APMs in the broader sense such as EB measures because, compared to SEC’s definition of NGFMs in Reg. G, the definition of APMs in the ESMA guidelines also includes EB measures (ESMA 2015). Furthermore, similar studies on the impact of regulatory measures would also be conceivable for Switzerland, regarding the *Directive on the Use of Alternative Performance Measures* of *SIX Swiss Exchange* (SIX Swiss Exchange 2019; similarly Oesch and Walser 2019) and the FMA guidance note in New Zealand (FMA 2017).

#### b) Determinants of APM Reporting

We recommend deepening the analysis of (potential) determinants of APM reporting that have not yet been explored or only to a limited extent. For example, research by Bansal et al. (2013) suggests that CEOs closer to retirement are less concerned about their reputation for transparent and accurate voluntary financial disclosures. Thus, the interaction between a manager’s age, disclosure incentives and disclosure policies may be a promising area for future research. In addition, industry affiliation as a determinant of APM reporting has been analysed in early studies, but overall industry-related effects still offer considerable room for further investigation. For example, future studies could investigate whether there are specific industry-related practices or characteristics that may influence APM disclosures (similarly Marques 2017). Furthermore, we note that ethical aspects of voluntary APM reporting have been addressed only rudimentarily in the context of APM research (e.g., Black 2016). Therefore, we suggest that ethical aspects and their implications should be researched more intensively, in particular the influence of corporate ethical aspects on APM reporting on the one hand as well as the consequences of strategically motivated (aggressive) APM reporting on the other (similarly Catuogno and Arena 2020).

#### c) Manager-Analyst Interactions

We propose a more in-depth analysis of the interaction between management and analysts (similarly Black et al. 2018). As previous studies suggest, managers and analysts often agree on the exclusion of specific earnings components when determining APMs (or street earnings), but it has also been found that managers’ and analysts’ decisions regarding the persistence of earnings exclusions sometimes diverge, with various reasons for this being discussed (e.g., Bentley et al. 2018; Bradshaw 2011; Berger 2005; Easton 2003). Thus, more in-depth analysis of the differences between managers’ and analysts’ earnings adjustments and the reasons for them could help disentangle managers’ and analysts’ motives in publishing APMs (similarly Black et al. 2018). However, as already noted by Beyer et al. (2010), one of the biggest challenges in this context is probably the consideration of interactions between the different sources of information, since to date little is known about the relationships between companies’ voluntary disclosure policies, mandatory disclosure requirements and the information provided by analysts. To investigate these relationships, researchers need to consider the interactions between the objectives of companies, managers, regulators, analysts, investors and other stakeholders, as well as the incentives these stakeholders have in determining the information environment observed in equilibrium (similarly Beyer et al. 2010). Developing such a comprehensive theory of accounting choice would require understanding when and how accounting information is used and the incentives of regulators, managers, analysts and other stakeholders. In addition, the development of such a theory is complicated by the fact that the link between analytical and empirical work on corporate information environment has so far been sparse. This in particular, because the analytical models developed are often very focused and detailed, while the empirical studies are often very broad in scope, making empirical testing of the theoretical models difficult, if not impossible (similarly Beyer et al. 2010).

#### d) Impact of Current Phenomena on APM Reporting

We encourage scholars to explore the impact of current phenomena such as economic and societal megatrends (e.g., digitalisation, connectivity or neo-ecology) and the ongoing COVID-19 pandemic on APM reporting. The latter applies in particular with regard to the practical implementation of ESMA’s Q&As on the application of the ESMA guidelines in context of the COVID-19 pandemic issued in April 2020 (ESMA 2020) as well as the *Statement on Importance of Disclosure about COVID-19* issued by the *International Organization of Securities Commissions* (IOSCO) in May 2020. In their statements, ESMA and IOSCO encourage issuers to provide investors with relevant and reliable information when making APM disclosures on the impact of the COVID-19 pandemic. In light of these statements, it would be interesting to find out whether listed companies have made changes to the definition and calculation of previously reported APMs in order to exclude (negative) earnings impacts due to the COVID-19 pandemic or whether they have introduced new APMs in response to the COVID-19 pandemic. Furthermore, it would be of particular interest to find out which types of earnings components are more frequently adjusted when determining APM in financial years that have been affected by the COVID-19 pandemic.

### *Implication 3: *Application of Alternative Research Methods

Our results show a high concentration on empirical research methods (66.1%). Besides archival (database or archive) research (55.1%), other empirical methods are used in about one tenth (11.0%) of the 410 articles analysed. Therefore, we consider it desirable to apply and combine alternative research methods that have rarely been used so far in order to achieve higher validity and reduce systematic errors of research findings (similarly Catuogno and Arena 2020). *First*, we propose conducting more survey research. On the managerial side, this may be a fruitful way to disentangle the motives leading managers to voluntarily disclose APMs. On the external stakeholder side, survey research may help to understand how analysts and investors perceive, process and use APM disclosures. In survey research, however, the difficulty arises from an empirical-methodological point of view that motives are neither observable nor surveyable (similarly Hitz 2010a), but at best the potential to mislead investors can be measured or observed (similarly Entwistle et al. 2006a).^28^ The empirical distinction of motives for APM reporting must therefore be based on the identification of patterns in management’s reporting behaviour which are consistent with the motives being discussed. This explains why, in previous research, the empirical distinction of motives for APM reporting is primarily based on the identification of patterns in management’s reporting behaviour. Probably for this reason, attempts to generalise the reporting behaviour of companies or to find a single dominant explanation for voluntary APM disclosures have so far failed, since the two motives of informative reporting and strategic disclosures are not mutually exclusive. Instead, empirical evidence suggests that the two motives are likely to co-exist, with the respective drivers of voluntary APM disclosures varying from company to company and from time to time, depending on the prevailing reporting incentives (similarly Young 2014). Accordingly, one of the greatest challenges for scholars is to develop empirical methods capable of disentangling competing explanations for APM reporting (similarly Young 2014).

This is why, *second*, we also suggest more experimental research, as this may be a fruitful way to disentangle the different APM reporting motives. In previous experimental research, to the best of our knowledge, no experiments have yet been conducted using managers as research subjects. We believe that the experimental study of managers could contribute to a better understanding of managers’ decisions (not) to disclose APMs, although it may be difficult to find a sufficient number of managers as research subjects to include in a sample. *Third *and finally, as already mentioned by Hitz (2010a), we note that the analysis of publication media using qualitative methods has hardly found any application (e.g., Guillamon-Saorin et al. 2017; Taylor and Keselj 2020). The use of qualitative methods such as content analysis may allow, for example, the identification of semantic patterns in the presentation of APM disclosures and their determinants (similarly Hitz 2010a), “so that investors are aware how different reporting practices of these measures can affect their decisions and market outcomes” (Marques 2017).

### *Implication 4:* Studying New Settings

The analysis of the research locations, origin of the authors and institutions shows a clear picture of over- and underrepresented research locations worldwide. While the North American region has already been extensively researched, other regions such as Europe, Central and South America, Australia, Asia and Africa have been little researched. As already noted by Catuogno and Arena (2020), most studies analyse APM reporting in a U.S. setting. Another driver, besides the U.S. setting, is the capital market orientation of companies reporting APMs, which, however, is also likely to be traced back to U.S. companies. Moreover, the sample design in the articles analysed is often based on selection indices such as the *S&P 500* or the *EURO STOXX 50*, which in terms of smaller countries raises the question of the relevance of such companies of national importance. However, “the phenomenon of non-GAAP earnings is not confined exclusively to the U.S.” (Catuogno and Arena 2020). Therefore, we suggest that more in-depth research should be conducted for other regions. In this respect, the most interesting regions for research are likely to be Europe, especially in light of ESMA’s recent regulatory initiatives and the Asian region, particularly due to its increased economic power over the last two decades. When conducting analyses of APMs in an European or Asian setting, it may be promising to replicate the methods of previous studies on the North American region and/or to conduct cross-national studies (similarly Hitz 2010a; Marques 2017).

Similar to the analysis of the research locations, a clear picture of overrepresented and underrepresented accounting regimes emerges. While U.S. GAAP has already been extensively researched, IFRS have been comparatively little researched so far. One possible reason for the focus on U.S. GAAP may be that APMs historically originated in the U.S., where the first regulatory measures were enacted in the early 2000s. Moreover, the focus of the literature on U.S. GAAP may be because capital markets tend to play a greater role in the U.S., which is reflected, for example, in the number of listed companies and stock market value. Besides these, however, a major reason why much more research has been conducted on APMs using U.S. GAAP data may be that data collection is much easier for U.S. GAAP compared to IFRS due to the structured form and data. This is in particular due to the availability of large and comprehensive datasets provided by company and analyst databases (e.g., *Thomson Reuters’ I/B/E/S, S&P Compustat *or *directEDGAR*), allowing for timely and cost-saving collection of data. Furthermore, unlike U.S. GAAP data, IFRS data is heterogeneous and diverse due to different national interpretations (‘dialects’) that may arise from different interpretations resulting from national translations or the influences of national regulations. For example, previous research suggests that national culture could undermine a consistent application of IFRS across countries and thus convergence of standards may not automatically lead to comparability in financial reporting (e.g., Tsakumis 2007; Doupnik and Richter 2004). According to this research, cultural differences often lead to companies in different countries applying and interpreting IFRS differently, particularly with regard to matters requiring professional judgement or when exercising discretion (similarly Hellmann et al. 2019). Taken together, these might be reasons why APMs in the IFRS regime have been less researched so far. This is why we propose a more detailed investigation of APMs in the context of IFRS, in particular because IFRS have gained increasing importance since their mandatory introduction for group accounting of listed companies in Europe in 2005 and in several other countries (e.g., Asian countries such as China, Japan or India). Regarding the analysis of European companies, the path taken by the European Union and ESMA towards digitalised financial reporting in iXBRL format in accordance with the European Single Electronic Format (ESEF) could enable easier and faster data collection, as the use of uniform taxonomies should significantly simplify and automate data collection processes. In terms of analysing new settings, a focus of future analyses could also be on analysing national differences in reporting APMs due to different national IFRS dialects. These findings could be particularly valuable to IASB’s current regulatory initiative on the presentation of a company’s performance in the income statement and the disclosures of MPMs in the accompanying notes according to ED/2019/7 (IASB 2019).

### *Implication 5:* Studying New Sources for APM Disclosures

The analysis of the data collection methods shows that the APMs studied so far are mainly obtained through document analysis of corporate publications, especially voluntary APM disclosures in earnings announcements or press releases (13.2%) and APMs disclosed in mandatory annual reports (11.0%). In contrast, APMs reported in other voluntary publications, such as speeches at the presentation of annual or quarterly results or at annual general meetings, presentations at analyst conferences, road shows, capital market days and other investor forums, conference calls with investors and the business press as well as investor magazines or strategy papers, have hardly been studied so far. Therefore, we suggest that, besides the publication media already intensively analysed, other publication media should also be examined to determine whether it contains APMs and, if so, whether and how the APM disclosures differ from those already intensively analysed. This analysis could reveal, for example, which publication media is primarily used by companies for reporting APMs (similarly Marques 2017) and also whether there are differences in the reporting of APMs in voluntary and mandatory financial reports (similarly Hitz 2010a). Another interesting medium for future analyses may be the financial communication of companies on social media platforms such as *Twitter, YouTube* or *Instagram*. This is particularly because corporate communication via social media has become increasingly important in recent years (e.g., Blankespoor et al. 2014; Jung et al. 2018; Bilinski 2019). Furthermore, compared to the analysis of other voluntary publication media the collection of social media data is less time-consuming due to the use of software solutions (e.g., *MAXQDA* software).

Another area of research that, to the best of our knowledge, has not yet been studied is the analysis of the coverage of APMs in the business press. The publication media analysed in previous studies are almost exclusively financial reports published by companies or street earnings made available in analyst databases, while the reporting of APMs in the business press has not yet been analysed at all. In this context, research questions arise regarding the diffusion and reception of APMs reported by companies in financial press (similarly Hitz 2010a). In particular, it would be interesting to find out whether APMs are viewed rather critically by the business press or whether they are taken up and adopted uncritically in media coverage. Since a search in renowned business newspapers such as the *Financial Times *or *The Wall Street Journal* for terms such as ‘non-GAAP’ or ‘alternative performance measures’ returns many obvious negative examples of companies’ APM reporting behaviour, one might assume that the coverage of APMs in the business press is (rather) negative. However, so far there is no scientific study that examines the media coverage of APM reporting based on scientific standards in order to prove that the business press is critical of companies’ APM reporting practices. In our view, the question of the diffusion and reception of companies’ APM disclosures in financial press is of particular interest because non-professional (i.e., retail) investors, for whom the previous empirical and experimental evidence suggests that they are potentially misled by APM reporting, probably often obtain their information from financial press.

### *Implication 6: *International Collaboration Between Authors

Looking at the total sample of 410 articles analysed in our SLR, the proportion of international collaborations is rather low. On average, authors collaborate across national borders in only one in ten articles (12.0%) and only four out of 410 articles (1.0%) are the result of collaboration between authors from more than two countries. These international collaborations are almost exclusively articles in academic journals (and sometimes working papers). In other words, international research collaborations take place almost exclusively in articles published in academic journals. Thus, when looking at the subsample including only academic articles, the proportion of international collaborations amounts to 15.8%. However, international collaborations between authors from more than two countries only take place in 1.8% of the 228 articles. Since such contributions resulting from international research collaborations are known to have a higher impact (measured by the number of average citations), such collaborations can stimulate the dialogue between researchers from different regions and backgrounds, leading to unique research findings and more creative research approaches (Nomaler et al. 2013).

## Concluding Remarks

The findings of our SLR suggest that APM reporting has become a well-established phenomenon in global financial reporting practice since the late 1990s and has been an internationally established component of financial communication for years, especially in the capital market communication of large, listed companies. Our analysis shows, that in the last decade a substantial amount of APM research has been published. Since 2010 the number of published articles on APMs has increased sharply reaching an all-time high in 2020 and 2021, indicating that the relevance of APM research has increased substantially over the last decade. In light of this increased relevance of APM research, in this paper we critically review the current state of research in more than 400 articles published in the field of APMs. We provide evidence that the phenomenon of voluntary APM reporting has been the subject of numerous research articles in academic journals as well as professional journals, with a focus on the North American region.

The purpose of our paper is to identify relevant research gaps that provide starting points for future research. For this purpose, our methodological approach strictly follows the SLR methodology proposed by Massaro et al. (2016) in order to minimise researcher idiosyncrasies and thus facilitate a decided derivation of research gaps based on a reliable and valid analytical framework which has been deductively derived from previous research on APMs. For this purpose, in our SLR, we present several future research paths which can be taken up and investigated in future research projects. However, while we believe that all these research paths are undoubtedly of great interest, some of them might be difficult or even impossible to implement in a particular research design. In light of these difficulties, in the following as *key takeaways* we would like to emphasise three research topics which we consider viable so that these can be researched directly without any major obstacles.

*First, *we suggest more research on companies’ APM disclosures in regions other than North America. In this respect, the most interesting regions for research are likely to be Europe, especially considering ESMA’s recent regulatory initiatives, and the Asian region, mainly because of its increased economic power over the last two decades. In particular, we propose more research on the impact of the ESMA guidelines on APM reporting by European listed companies. In this context, it seems promising to replicate previous methodologies from U.S. studies to draw comparisons between the regulatory impact in the U.S. and in Europe.

*Second*, we recommend more research on APM reporting in the IFRS accounting regime. While U.S. GAAP have already been extensively researched, APMs in the IFRS accounting regime have been less researched so far. For this reason, we propose more research on APMs in the context of IFRS, in particular in light of the IASB’s current regulatory initiative on the presentation of a company’s performance in the income statement and the disclosures of MPMs in the accompanying notes according to ED/2019/7 (IASB 2019). Such regulatory action by the IASB requires critical analysis of the impact of future regulation on APM reporting and the relationship of voluntarily disclosed APMs to mandatorily disclosed MPMs. For example, one interesting question regarding the proposed amendments to IAS 1 is whether these changes would affect investors demand for APMs and managers’ decisions to disclose APMs (similarly Black et al. 2018).

*Third*, we propose to synthesise previous research findings. Depending on the epistemological position, different approaches can be used to synthesise research findings which all have the objective in common to summarise the results of a large number of empirical studies in a coherent manner (Tranfield et al. 2003). Two interesting methods that we believe are particularly useful for synthesising the previous research findings in the field of APM are meta-analyses and co-citation analyses. For example, conducting a meta-analysis would be a promising way to estimate the overall effects (e.g., in terms of determinants for APM reporting) and also to detect moderator variables which are responsible for the overall effect (e.g., the frequency of APM reporting and/or the quality of exclusions) being stronger or weaker. Also, it would be valuable to conduct a document co-citation analysis, allowing to map the interconnections between scientific publications and scholars using network archival methods (e.g., Chen 2006). In this respect, the results of our SLR can serve as a starting point for such research syntheses.^29^

## Limitations

By its nature, our study has some limitations in terms of the SLR methodology applied, which must be considered when interpreting the results. *First* of all, despite the structured approach of the SLR it cannot be entirely ruled out that potentially relevant articles were overlooked and the quality of the results could possibly be improved by considering even more databases and keywords (*sampling bias*). *Second*, our SLR considers only articles written in German and English. Therefore, it cannot be ruled out that articles published in other languages might provide meaningful insights for APM research (*language bias*). *Third*, while the SLR includes unpublished work such as working/discussion/conference papers in the sample, which leads to a more comprehensive coverage of the literature on APMs, it can also lead to limitations (e.g., because preliminary conclusions in working papers may change). Accordingly, the findings of our SLR represent a current snapshot of the literature on APMs and do not necessarily reflect the conclusions that will emerge from future revisions of these working papers (*revision bias*) (similarly Black et al. 2018). *Fourth*, while citation analysis can provide information on the relative influence of an article, it lacks the ability to identify networks of links between scholars (*citation bias*) (similarly Üsdiken and Pasadeos 1995). *Fifth*, although the transparent SLR process attempts to minimise subjective influence, author bias remains because authors are never completely objective and their interpretations of the results depend on the subjective knowledge of the APM discipline (*author bias*). *Sixth*, further limitations may arise from a different classification of journals as ‘academic journals’ or ‘professional journals’ compared to the *VHB-JOURQUAL3* ranking we apply in our SLR (VHB 2015). Overall, when interpreting the results of our SLR on APMs, it should be kept in mind “that structured literature reviews are not a panacea providing definitive answers, rather they aim to identify where research is currently lacking and offer pathways for future research” (Massaro et al. 2015).

## Supplementary Information

The online version of this article contains supplementary material, which is available to authorised users.

## Footnotes

1 APMs are to be strictly distinguished from so-called ‘pro forma financial statements’ used by companies to present the effects, for example, of corporate transactions or changes in accounting methods in form of an ‘as-if’ view in order to exclude discontinuities in accounting. Such statutory pro forma disclosures will not be discussed further in the following. For a detailed distinction, see Heiden (2006).

2 In Europe, these types of performance measures are commonly referred to as ‘alternative performance measures’, while in the U.S., for example, they are known as ‘non-GAAP measures’, in Australia as ‘non-IFRS financial information’ and in South Africa as ‘headline earnings’ (Marques 2017).

3 In this paper, we use the term ‘article’ synonymously to refer both to journal articles and to other types of publications, such as contributions to collective works, working papers or monographs. When we need to make the distinction between different types of publications, we use the exact terms.

4 In this context, Heiden (2006) speaks of a performative ‘as if’ through which management retrospectively establishes its own determination of ‘reality’.

5 For a comprehensive overview of the variety of performance measures found in financial reporting practice, see Pizzo (2020); Heiden (2006); Küting and Heiden (2003).

6 For a similar distinction, see Hitz (2010b); Hitz and Jenniges (2008); Sellhorn et al. (2014); Reimsbach (2014). For a detailed systematisation of different types of APMs based on 17 characteristics, see Küting and Heiden (2003).

7 In this context, Aubert und Grudnitski (2014) refer to so-called ‘idiosyncratic’ adjustments by management due to its access to company-internal (i.e., private) information.

8 For example, if the majority of analysts covering a particular company adjust stock option expenses, those expenses would also be adjusted in I/B/E/S database (Thomson Reuters 2009).

9 Regarding the regulatory requirements in the U.S. for reporting NGFMs, see in detail Di Fabio and Roncagliolo (2020).

10 Regulation (EC) No. 1606/2002 of the European Parliament and of the Council of 19 July 2002, OJ L 243/1.

11 The presentation of additional subtotals and disaggregated line items does not include the presentation of any items of income or expense as extraordinary items, which is expressly prohibited in both the income statement and the accompanying notes (IAS 1.87).

12 The presentation of the EB measure ‘EBITDA’ is not permissible if the income statement is structured according to the cost-of-sales method, since its integration as a subtotal inevitably leads to an impermissible mixing of the cost-of-sales method and the nature-of-expense method (IASB 2017).

13 Regarding ED/2019/7 in general, see in detail Grimm and Heintges (2020); Heintges and Naumann (2020); Bach and Berger (2020). Regarding the predicted practical consequences, see in detail Grote and von Keitz (2020); Kirsch (2020a, b).

14 The disclosure of MPMs as subtotals on the face of the income statement is explicitly prohibited (IASB 2019).

15 Directive (EC) No. 2004/109 of the European Parliament and of the Council of 15 December 2004, OJ L 390/38.

16 Regulation (EU) No 596/2014 of the European Parliament and of the Council of 16 April 2014, OJ L 173/1.

17 Regulation (EU) No 1095/2010 of the European Parliament and of the Council of 24 November 2010, OJ L 331/84.

18 The essential contents of our research protocol are presented in detail in *Annex 1*.

19 For the purpose of this paper CPY = CI / (2021 − publication year) for articles published before 2020 (similarly Dumay and Cai 2014). For articles published in 2021, CPY equals CI.

20 A detailed description of the analytical framework is provided in *Annex 4*.

21 All other sources were left out of the literature search in the databases. For example, we do not consider ‘trade publications’, ‘magazines’, ‘newspapers’, ‘websites’, ‘market research reports’, ‘industry profiles’ or ‘country reports’. We also exclude working/discussion/conference papers identified in the literature search from the sample if it was obvious that a published journal article based on the respective paper is already included in the sample. Working/discussion/conference papers for which a more recent version was obviously already included in the sample were also excluded.

22 A complete list of all 410 articles included in the sample is provided in *Annex 3*.

23 Supplementary analysis of the authors, institutions and journals is provided in *Annex 6*.

24 Supplementary analysis on CPY and CI is provided in *Annex 5*.

25 These two articles are not included in the ‘top 25’ articles with the highest CPYs in Table 2, since articles published within the period just before the SLR was conducted (2021) were excluded from all individual CPY scores (see in detail Sect. 4.1 and *Annex 5*).

26 For example, Doyle et al. (2003) focus on both the *adversarial investor influence as a motive *via *predictive ability of earnings components excluded from APMs* (here defined as *primary research focus*) as well as the *adversarial investor influence as a fact* via *capital market-based studies* (here defined as *secondary research focus*).

27 E.g., CF 1.2 for accounting in accordance with IFRS or CON 1.37 for accounting in accordance with U.S. GAAP.

28 For example, as Attas (1999) states regarding advertisement, “[t]he misleadingness … is not something that can be determined empirically”.

29 The complete list of all 410 articles included in our sample is provided in *Annex 3.*
