# Supplementary material for: Alternative Performance Measures: A Structured Literature Review of Research in Academic and Professional Journals
Source: Schmalenbach Z Betriebswirtsch Forsch. 2022 Sep 6;74(3):389–451. doi: 10.1007/s41471-022-00138-8 (PMC9446604; doi:10.1007/s41471-022-00138-8)
Supplement: Supplementary file 1 — The online version of this article contains supplementary material, which is available to authorised users. [file 41471_2022_138_MOESM1_ESM.pdf]

Annexes [Supplementary Information (SI)]

Annex 1: Summary of the research protocol

In order to find out what is already known about APMs ('insights'), provide a critical analysis of the focus of previous APM research ('critique') and to identify new, relevant research paths ('transformative redefinition'), we apply SLR methodology, which has proven its usefulness in a broad range of accounting fields such as intellectual capital research (e.g., Cuozzo et al. 2017; Secundo et al. 2018), research on integrated reporting (Dumay et al. 2016), public value and public sector accounting research (Bracci et al. 2019), internal audit research (Roussy and Perron 2018) and research on CEO compensation (Winschel and Stawinoga 2019). To develop our SLR on APMs, we use the (slightly modified) 10-step process as proposed by Massaro et al. (2016).

1. Research protocol

According to Petticrew and Roberts (2006), "[i]t is essential to write a protocol stating the review question, the methods to be used, the study types and designs which the reviewer intends to locate, and by what means, and how these studies will be appraised and synthesized". Therefore, as a first step of the SLR on APMs a literature review protocol was developed. In the literature review protocol, it was noted that, to the best of our knowledge, hitherto no other such comprehensive SLR of research articles in academic and professional journals has been presented at a conference, published in a journal or otherwise. Although this in itself is not (yet) a research gap, we concluded that there is a need for such an SLR. As Petty and Guthrie (2000) note, there are two objectives for the study of an emerging field of research such as APMs. First, it is necessary to "categorise it in a way that provides a useful understanding of how and why the [APM] movement has developed in the way it has" (Petty and Guthrie 2000). Second, "a platform [has to be created] to identify those avenues for future research that we consider likely to deliver results for understanding the nature, impact and value of [APMs]" (Petty and Guthrie 2000). Accordingly, the aim of our SLR is to pursue these objectives and to empirically justify the research gaps we identify (similarly Dumay et al. 2016). In summary, our objective is to gain insights into APM research to find out what is already known about APMs ('insights'), provide a critical analysis of the focus of previous APM research ('critique') and to identify new, relevant research paths ('transformative redefinition').

2. Research questions

The second step is to define the research questions. To develop an SLR as an instrument of critical research Massaro et al. (2016) draw upon three tasks of critical research: 'insights', 'critique' and 'transformative redefinition' according to Alvesson and Deetz (2000). Accordingly, our SLR intends to answer the following three core research questions:

- *RQ1: How has the literature on APMs developed so far?*
- *RQ2: What is the focus and critique of APM literature?*
- *RQ3: What is the future for APM research?*

3. Literature search

Conducting an SLR requires careful selection of the underlying body of literature (Dixon-Woods 2011). For this reason, a comprehensive literature search was carried out in a third step. In previous SLRs, several different search strategies were used such as keyword search in a particular research field (e.g., Englund and Gerdin 2014; Hoque 2014), citation classics (e.g., Serenko and Dumay 2015; Biemans et al. 2010), single journal analysis (Guthrie and Parker 2011) or keyword search in an emerging research field (e.g., Massaro et al. 2016; Bracci et al. 2019). According to Massaro et al. (2016), a keyword search is appropriate when researchers want to focus on papers published in a particular research field. Therefore, we conducted a comprehensive keyword search. However, keyword search requires that the keywords and the databases to be searched are defined in advance. Although in academic research, the peer-review process may be considered synonymous with the quality of published research (Easterby-Smith et al. 2012), according to Massaro et al. (2016), "other sources

can be equally valid to review a particular field and … [t]hus, researchers should consider not confining SLRs solely to journal articles”. In German-speaking countries in particular, there are a large number of other relevant sources, so that research is not only reflected in peer-reviewed academic journals (e.g., Wagenhofer 2006; Küting et al. 2013; Kußmaul et al. 2017). In order to identify as broad a body of APM literature, the literature search was therefore not limited to articles in prestigious peer-reviewed journals, like in previous literature reviews (e.g., Arena et al. 2021; Catuogno and Arena 2020; Marques 2017). Instead, our SLR also takes into account other publication media such as articles in lower-ranked journals (e.g., professional journals) according to the *VHB-JOURQUAL3* ranking (VHB 2015) as well as other publication sources, in particular monographs, contributions to collective works (e.g., *Festschriften*) and working/discussion/conference papers. For this purpose, a comprehensive literature search was conducted in the five literature databases *Scopus*, *EconBiz*, *Google Scholar*, *EBSCO Business Source Premier* and *ISI Web of Science*. The keywords used to search the databases were identified in advance in a rudimentary literature search in order to carefully “select terms that will generate the data being sought” (Cronin et al. 2008). When conducting the literature search, these pre-determined keywords were supplemented with additional keywords identified during the literature search. Based on these newly identified keywords, the databases were subsequently searched again. Because searching entire databases turned up too much irrelevant articles, the keyword search was performed within titles, abstracts and keywords only using phrase search (similarly Massaro et al. 2016). For example, for keywords consisting of several compound words, such as ‘alternative performance measures’, only the fixed expressions were searched for. In order to include all articles published by December 2021 lately, no time limits were set. The full list of keywords selected, the search strings used to search the databases and the number of search results are presented in *Annex 2*.

In order to be included in the sample, the articles identified in the literature search must fulfil some general requirements (*inclusion criteria*). First, the article is written in English or German. Second, the research topic of the article must substantively focus on APMs. Studies from research areas other than APMs were excluded from the sample even if they contain one of the searched keywords in the title, abstract or keywords. For example, the keyword ‘EBIT’ is also used in the research field of nuclear physics as an abbreviation for so-called ‘electron beam ion traps’ (e.g., Currell and Fussmann 2005). For the application of the second criterion, the title, abstract and, where necessary, full text of the articles were reviewed in detail to determine whether they address the research topic of APMs in terms of content. Articles which were not included in the sample due to the application of the *inclusion criteria* were documented in a *Microsoft Excel* file along with the reason for exclusion.

The literature search resulted in a total of 2,337 articles. Of these, 1,927 articles were removed from the sample due to the application of the *inclusion criteria*, leaving a sample with a total of 410 articles which were analysed in detail. The complete list of all 410 articles included in the sample is provided in *Annex 3*. For all articles a full version was obtained and stored in an *EndNote* database with complete referencing details (similarly Guthrie et al. 2012; Guthrie and Murthy 2009; Broadbent and Guthrie 2008). Furthermore, in order to complete the sample, an additional checking procedure was carried out (similarly Winschel and Stawinoga 2019). As part of this additional checking procedure the five respective databases were continuously searched for newly published articles throughout the analysis, writing and peer-review process. As a result of this additional checking procedure, 52 articles were added to the preliminary data set and included in the final sample of 410 articles.

4. Citation analysis

The fourth step of the SLR is citation analysis to measure the article impact (Massaro et al. 2016). The analysis of citations allows for a better understanding of how APM literature has evolved by examining the impact of the articles (Massaro et al. 2016). In addition, interesting findings emerge from the analysis of the impact of various authors, institutions and journals by searching for the ‘superstar’ (or ‘Matthew’) effect, which “appears when a small fraction of researchers or institutions produce the most works and attract a disproportionate number of

citations" (Serenko and Dumay 2015; see also Merton 1968; Merton 1988; Rosen 1981). To measure the impact of the identified articles, authors and journals, we use 'citations' (CI) and 'citations per year' (CPY), applying the approach according to Dumay (2014). CIs "are the total citation for the article … on a specific day" (Massaro et al. 2016) and CPY "is citations divided by the number of the years between the current year of analysis and publication year" (Massaro et al. 2016). For the purpose of this paper CPY = CI / (2021 – publication year) for articles published before 2021 (similarly Dumay and Cai 2014). For articles published in 2021, CPY equals CI. Like Dumay (2014), we use data from *Google Scholar* (as opposed to *ISI Web of Science* or *Scopus*) to collect citations. This is for two reasons. First, because "Google Scholar … indexes all categories of publications, and counts citations from non-peer-reviewed works, such as professional journals, government documents, and newspapers" (Serenko and Bontis 2013; see also Dumay 2014). Second, because "Google Scholar is currently considered a leading tool in citation analysis" (Serenko and Bontis 2013; see also Harzing and van der Wal 2008). For these reasons, we downloaded from *Google Scholar* the articles' citation data on 1 January 2021 and subsequently updated the citation data on 29 January 2022. Like in previous SLRs, we note that articles published within the period just before the SLR was conducted (2021) were excluded from all individual CPY scores because there was not enough time to cite the articles.

5. Analytical framework

"Essentially a structured literature review is a form of content analysis whereby the unit of analysis is the article, as opposed to words, sentences or paragraphs, as is commonly found in content analysis research" (Massaro et al. 2015; see also Krippendorff 2013). In order to conduct an SLR, categories need to be defined which are embedded in an analytical framework. Massaro et al. (2016) "advocate developing specific analytical frameworks, derived from previous reviews of related fields". Accordingly, the analytical framework used in our SLR is essentially based on similar analytical frameworks which have already proven successful in other (structured) literature reviews in the field of accounting research (e.g., Massaro et al. 2015; Serenko and Dumay 2015; Dumay and Garanina 2013; Guthrie et al. 2012; Broadbent and Guthrie 2008). Furthermore, according to Krippendorff (2013), categories should not be considered as given, but rather emerge in the reading process and thus imply the experience of analysis as a competent reader. Accordingly, the SLR builds on these categories, which were (slightly) modified during the coding process. In order to ensure a precise delineation between the variables of the categories and a clear assignment of the articles to the categories, the analytical framework was subjected to a pre-test (similarly Broadbent and Guthrie 2008; Guthrie et al. 2012; Guthrie and Murthy 2009). In this pre-test, the 20 most cited articles were coded based on both the abstracts and the full text of the articles, resulting in a slightly modified coding scheme.

6. Coding

According to Massaro et al. (2016), there are basically two different methods for coding articles in an SLR: manual and computer-aided coding. For our SLR on APMs we use manual coding because "[t]he advantage of manual coding is when words with similar meaning … are encountered, they can be understood in their true sense and coded accordingly" (Guthrie et al. 2012). Nevertheless, manual coding does not preclude the use of computer and software (Massaro et al. 2016). According to Bazeley and Jackson (2013), "a computer is not intended to supplant time-honoured ways of learning from data, but to increase the effectiveness and efficiency of such learning". Therefore, we use *MAXQDA* software by manually assigning the 410 articles in the sample to the categories of the analytical framework (see in detail Mayring 2015; Kuckartz 2016; Bryman and Bell 2015).

7. Reliability

Since SLRs are a form of content analysis, it is necessary to develop several forms of control and triangulation to reduce bias and ensure that both the analytical framework and the codes are reliable (Massaro et al. 2016; Yin 2014). The use of reliability measures can be valuable in demonstrating that the results have been produced with conceivable precaution against known pollution, bias and distortion, whether intentional or accidental, and mean

the same thing to anyone who uses them (Krippendorff 2013). Therefore, a reliability test was conducted using *Krippendorff's α* as reliability measure, because of all different methods that can be used to determine reliability in content analysis *Krippendorff's α* is robust (Krippendorff 2013; Hayes and Krippendorff 2007), because "it can be used regardless of the number of observers, levels of measurement, sample sizes, and presence or absence of missing data" (Hayes and Krippendorff 2007). When using *Krippendorff's α* as reliability measure, according to Krippendorff (2013), researchers can rely on variables with reliabilities of $\alpha \geq 0.8$ and consider variables with reliabilities between $0.67 \leq \alpha < 0.8$ only for drawing preliminary conclusions. To determine *Krippendorff's α*, the 382 articles initially included in the sample (before the additional checking procedure) were first coded independently by one of the authors. In a second step, the 382 articles were divided equally between two students enrolled in the master's programme in 'Service Management' at a German university and coded by them independently. Subsequently, *Krippendorff's α* was calculated for the independent data sets. For all seven categories, the values are above, or regarding the categories 'B. Research focus' and 'F. Types of APMs' at least close to, the target reliability measure of *Krippendorff's* $\alpha \geq 0.8$. The average *Krippendorff's α* is 0.910. Therefore, we assume that the analytical framework and the results of the analysis of the APM articles can be considered reliable.

8. Validity

Validity tests are used in empirical studies to ensure the accuracy of the findings obtained (Franklin et al. 2010). Regarding validity, a distinction is made between internal, external and construct validity (White and McBurney 2012). Saunders et al. (2016) define *internal validity* as "the extent to which the findings can be attributed to the interventions rather than any flaws … in research design". Internal validity thus seeks to establish causal relationships (Döring and Bortz 2016; White and McBurney 2012). In order to establish internal validity for our SLR, we built on analytical frameworks which have already proven successful in other (structured) literature reviews in the field of accounting research (e.g., Massaro et al. 2015; Serenko and Dumay 2015; Dumay and Garanina 2013; Guthrie et al. 2012; Broadbent and Guthrie 2008). In addition, the systematic coding and continuous logging of the evaluation process in *MAXQDA* ensures transparency and traceability and minimises researcher idiosyncrasies (similarly Winschel and Stawinoga 2019). Furthermore, internal validity is promoted by pre-testing the analytical framework (similarly Winschel and Stawinoga 2019).

External validity addresses the question of whether "a study's research findings can be generalised to other relevant settings or groups" (Saunders et al. 2016) and depends significantly on the quality of the study design and the sampling procedure (Döring and Bortz 2016). Accordingly, to establish external validity, the SLR is based on the standardised SLR methodology as proposed by Massaro et al. (2016), which follows a replicable and transparent procedure for the systematic analysis of a defined body of literature. In addition, as the SLR took several months to complete, we implemented an additional checking procedure throughout the analysis, writing and peer-review process to further promote external validity (similarly Winschel and Stawinoga 2019). In this context, recently published articles were reviewed to determine whether they substantively focus on APMs and meet our *inclusion criteria*.

Construct validity is "the extent to which a set of questions … actually measures the presence of the construct … intended to measure" (Saunders et al. 2016) and thus refers to the validity of the interpretation of empirical data as indicators of theoretical constructs (Döring and Bortz 2016). With regard to the SLR, methodologically and in terms of content, construct validity is promoted by the structured SLR approach for the derivation of study criteria as well as for the collection and evaluation of articles (similarly Winschel and Stawinoga 2019). In addition, construct validity is further promoted by using a variety of keywords and databases as part of the SLR, as well as using *inclusion criteria* for structuring the data collection process and protocolling intermediate findings as part of the evaluation process (similarly Winschel and Stawinoga 2019).

References

Alvesson, Mats, and Stanley Deetz. 2000. *Doing Critical Management Research*. London et al.: Sage Publications.

Arena, Claudia, Simona Catuogno, and Nicola Moscariello. 2021. The unusual debate on non-GAAP reporting in the current standard practice. The lens of corporate governance. *Journal of Management & Governance* 25: 655-684.

Bazeley, Patricia, and Kristin Jackson. 2013. *Qualitative Data Analysis with NVivo*. London et al.: Sage Publications.

Biemans, Wim, Abbie Griffin, and Rudy Moenaert. 2010. In Search of the Classics: A Study of the Impact of JPIM Papers from 1984 to 2003. *Journal of Product Innovation Management* 27: 461-484.

Bracci, Enrico, Luca Papi, Michele Bigoni, Enrico Deidda Gagliardo, and Hans-Jürgen Bruns. 2019. Public value and public sector accounting research: a structured literature review. *Journal of Public Budgeting, Accounting & Financial Management* 31: 103-136.

Broadbent, Jane, and James Guthrie. 2008. Public sector to public services: 20 years of “contextual” accounting research. *Accounting, Auditing & Accountability Journal* 21: 129-169.

Bryman, Alan, and Emma Bell. 2015. *Business Research Methods*. Oxford: Oxford University Press.

Catuogno, Simona, and Claudia Arena. 2020. Determinants and Consequences of Non-GAAP Disclosure: A Review of the Literature. In *Reporting Non-GAAP Financial Measures*, ed. Nicola Moscariello and Michele Pizzo, 38-65. Newcastle: Cambridge Scholars Publishing.

Cronin, Patricia, Frances Ryan, and Michael Coughlan. 2008. Undertaking a literature review: a step-by-step approach. *British Journal of Nursing* 17: 38-43.

Cuozzo, Benedetta, John Dumay, Matteo Palmaccio, and Rosa Lombardi. 2017. Intellectual capital disclosure: a structured literature review. *Journal of Intellectual Capital* 18: 9-28.

Currell, Fred, and Gerd Fussmann. 2005. Physics of electron beam ion traps and sources. *IEEE Transactions on Plasma Science* 33: 1763-1777.

Dixon-Woods, Mary. 2011. *Systematic Reviews and Qualitative Methods*. Los Angeles et al.: Sage Publications.

Döring, Nicola, and Jürgen Bortz. 2016. *Forschungsmethoden und Evaluation in den Sozial- und Humanwissenschaften*. Berlin et al.: Springer.

Dumay, John, and Linlin Cai. 2014. A review and critique of content analysis as a methodology for inquiring into IC disclosure. Journal of Intellectual Capital 15: 264-290.

Dumay, John, and Tatiana Garanina. 2013. Intellectual capital research: a critical examination of the third stage. *Journal of Intellectual Capital* 14: 10-25.

Dumay, John, Cristiana Bernardi, James Guthrie, and Paola Demartini. 2016. Integrated reporting: A structured literature review. *Accounting Forum* 40: 166-185.

Dumay, John. 2014. 15 years of the Journal of Intellectual Capital and counting. *Journal of Intellectual Capital* 15: 2-37.

Easterby-Smith, Mark, Richard Thorpe, and Paul Jackson. 2012. *Management Research*. London et al.: Sage Publications.

Englund, Hans, and Jonas Gerdin. 2014. Structuration theory in accounting research: Applications and applicability. *Critical Perspectives on Accounting* 25: 162-180.

Franklin, Cynthia S., Patricia A. Cody, and Michelle Ballan. 2010. Reliability and Validity in Qualitative Research. In *The Handbook of Social Work Research Methods*, ed. Bruce A. Thyer, 355-374. Thousand Oaks: Sage Publications.

Guthrie, James, and Lee D. Parker. 2011. Reflections and projections: 25 years of interdisciplinary perspectives on accounting, auditing and accountability research. *Accounting, Auditing & Accountability Journal* 25: 6-26.

Guthrie, James, and Vijaya Murthy. 2009. Past, present and possible future developments in human capital accounting. *Journal of Human Resource Costing & Accounting* 13: 125-142.

Guthrie, James, Federica Ricceri, and John Dumay. 2012. Reflections and projections: A decade of Intellectual Capital Accounting Research. *The British Accounting Review* 44: 68-82.

Harzing, Anne-Wil K., and Ron van der Wal. 2008. Google Scholar as a new source for citation analysis. *Ethics in Science and Environmental Politics* 8: 61-73.

Hayes, Andrew F., and Klaus Krippendorff. 2007. Answering the Call for a Standard Reliability Measure for Coding Data. *Communication Methods and Measures* 1: 77-89.

Hoque, Zahirul. 2014. 20 years of studies on the balanced scorecard: Trends, accomplishments, gaps and opportunities for future research. *The British Accounting Review* 46: 33-59.

Krippendorff, Klaus. 2013. *Content Analysis – An Introduction to Its Methodology*. Thousand Oaks et al.: Sage Publications.

Kuckartz, Udo. 2016. *Qualitative Inhaltsanalyse. Methoden, Praxis, Computerunterstützung*. Weinheim et al.: Beltz Juventa.

Kußmaul, Heinz, Hartmut Bieg, Claus-Peter Weber, Gerd Waschbusch, Alexander Baumeister, Wolfgang Wegener, Michael Olbrich, Alois Paul Knobloch, René Schäfer, and Vassil Tcherveniachki. 2017. Normative theorie- und praxisbezogene Betriebswirtschaftslehre – Methodenpluralismus am Beispiel der Betriebswirtschaftlichen Steuerlehre und der Rechnungslegung. *Der Betrieb* 70: 1337-1343.

Küting, Karlheinz, Heinz Kußmaul, Hartmut Bieg, Claus-Peter Weber, Gerd Waschbusch, Alexander Baumeister, Wolfgang Wegener, Michael Olbrich, and Alois Paul Knobloch. 2013. Saarbrücker Plädoyer für eine normative theorie- und praxisbezogene Betriebswirtschaftslehre. *Der Betrieb* 66: 2097-2099.

Marques, Ana. 2017. Non-GAAP earnings: international overview and suggestions for future research. *Meditari Accountancy Research* 25: 318-335.

Massaro, Maurizio, John Dumay, and Andrea Garlatti. 2015. Public sector knowledge management: a structured literature review. *Journal of Knowledge Management* 19: 530-558.

Massaro, Maurizio, John Dumay, and James Guthrie. 2016. On the shoulders of giants: undertaking a structured literature review in accounting. *Accounting, Auditing & Accountability Journal* 29: 767-801.

Mayring, Philipp. 2015. *Qualitative Inhaltsanalyse – Grundlagen und Techniken*. Weinheim et al.: Beltz Verlag.

Merton, Robert K. 1968. The Matthew Effect in Science. *Science* 159: 56-63.

Merton, Robert K. 1988. The Matthew Effect in Science, II: Cumulative Advantage and the Symbolism of Intellectual Property. *Isis* 79: 606-623.

Petticrew, Mark, and Helen Roberts. 2006. *Systematic Reviews in the Social Sciences*. Malden et al.: Blackwell Publishing.

Petty, Richard, and James Guthrie. 2000. Intellectual capital literature review. *Journal of Intellectual Capital* 1: 155-176.

Rosen, Sherwin. 1981. The Economics of Superstars. *The American Economic Review* 71: 845-858.

Roussy, Mélanie, and Alexandre Perron. 2018. New Perspectives in Internal Audit Research: A Structured Literature Review. *Accounting Perspectives* 17: 345-385.

Saunders, Mark, Philip Lewis, and Adrian Thornhill. 2016. *Research Methods for Business Students*. Essex: Pearson.

Secundo, Giustina, Maurizio Massaro, John Dumay, and Carlo Bagnoli. 2018. Intellectual capital management in the fourth stage of IC research. *Journal of Intellectual Capital* 19: 157-177.

Serenko, Alexander, and John Dumay. 2015. Citation classics published in knowledge management journals. Part I: articles and their characteristics. *Journal of Knowledge Management* 19: 401-431.

Serenko, Alexander, and Nick Bontis. 2013. Investigating the current state and impact of the intellectual capital academic discipline. *Journal of Intellectual Capital* 14: 476-500.

VHB. 2015. *List of journals in VHB-JOURQUAL3*. https://vhbonline.org/en/vhb4you/vhb-jourqual/vhb-jourqual-3/complete-list. Accessed 17 January 2022.

Wagenhofer, Alfred. 2006. Management Accounting Research in German-Speaking Countries. *Journal of Management Accounting Research* 18: 1-19.

White, Theresa L., and Donald H. McBurney. 2012. *Research Methods*. Wadsworth: Wadsworth Publishing.

Winschel, Julija, and Martin Stawinoga. 2019. Determinants and effects of sustainable CEO compensation: a structured literature review of empirical evidence. *Management Review Quarterly* 69: 265-328.

Yin, Robert. 2014. *Case Study Research*. Los Angeles et al.: Sage Publications.

Annex 2: Search strings and results

No.	Keywords	Number of articles before inclusion criteria: Scopus[a]	EconBiz[b]	Google Scholar[c]	EBSCO Business Source Premier[d]	ISI Web of Science[e]	Number of articles after inclusion criteria: Scopus[a]	EconBiz[b]	Google Scholar[c]	EBSCO Business Source Premier[d]	ISI Web of Science[e]
1	Adjusted EPS	4	2	3	0	3	2	2	2	0	2
2	Adjusted earnings	57	20	33	60	19	12	9	10	11	12
3	Alternative earnings	33	11	23	43	17	7	6	8	7	4
4	Alternative performance measures	48	6	35	38	26	7	3	14	4	5
5	Earnings before	255	38	58	235	137	10	13	16	13	7
6	Earnings exclusions	10	9	10	9	9	10	9	8	9	9
7	Earnings forecast exclusions	1	1	1	1	1	1	1	1	1	1
8	EBIT	118	47	21	128	89	4	16	4	1	2
9	EBITA	22	4	3	5	2	1	1	2	0	1
10	EBITDA	182	95	30	209	122	8	26	6	12	12
11	EBT	27	24	18	50	27	0	0	0	0	0
12	GAAP-adjusted	2	2	4	3	2	2	2	3	3	2
13	Non-GAAP	152	133	127	136	126	120	122	93	104	99
14	Non-Generally Accepted Accounting Principles	14	11	8	19	15	13	11	7	15	14
15	One-time gains	8	2	2	4	3	1	1	1	1	1
16	Proforma	31	17	44	38	9	4	7	3	4	4
17	Pro forma	219	182	118	253	187	100	122	46	95	87
18	Reg G	4	6	7	3	2	4	4	4	1	2
19	Regulation G	24	23	30	20	19	22	20	18	16	17
20	Street earnings	52	34	38	42	47	41	34	27	37	39
21	Street exclusions	2	2	4	2	2	2	2	2	2	2
**Total number of articles per database**		**969**	**477**	**492**	**1039**	**615**	**184**	**242**	**172**	**186**	**153**
**Total number of articles**				**2,337**					**410**		

Tab. 1: Keywords and literature search results

[a] Search for the phrases only in the subject areas (SUBJAREA) ‘Business, Management and Accounting’ (BUSI) and ‘Economics, Econometrics and Finance’ (ECON), since a search in all subject areas would have yielded too many thematically inappropriate search results that do not substantively focus on APMs. Accordingly, the *Scopus* search string used is documented as follows: *TITLE-ABS-KEY ("Adjusted EPS" OR "Adjusted earnings" OR "Alternative earnings" OR "Alternative performance measures" OR "Earnings before" OR "Earnings exclusions" OR "Earnings forecast exclusions" OR "EBIT" OR "EBITA" OR "EBITDA" OR "EBT" OR "GAAP-adjusted" OR "Non-GAAP" OR "Non-Generally Accepted Accounting Principles" OR "One-time gains" OR "Proforma" OR "Pro forma" OR "Reg. G" OR "Regulation G" OR "Street earnings" OR "Street exclusions") AND (LIMIT-TO (SUBJAREA , "BUSI") OR LIMIT-TO (SUBJAREA , "ECON")).*

[b] Search for the phrases only in the fields ‘Title’ and ‘Subject’, since a search in the abstract and keywords is not possible at *EconBiz* and a search in all fields would have yielded too many thematically inappropriate search results that do not substantively focus on APMs. Accordingly, the *EconBiz* search string used is documented as follows: *(title: (("Adjusted EPS") OR ("Adjusted earnings") OR ("Alternative earnings") OR ("Alternative performance measures") OR ("Earnings before ") OR ("Earnings exclusions") OR ("Earnings forecast exclusions") OR ("EBIT") OR ("EBITA") OR ("EBITDA") OR ("EBT") OR ("GAAP-adjusted") OR ("Non-GAAP") OR ("Non-generally Accepted Accounting Principles") OR ("One-time gains") OR ("Proforma") OR ("Pro forma") OR ("Reg. G") OR ("Regulation G") OR ("Street earnings") OR ("Street exclusions"))) OR (subject: (("Adjusted EPS") OR ("Adjusted earnings") OR ("Alternative earnings") OR ("Alternative performance measures") OR ("Earnings before") OR ("Earnings exclusions") OR ("Earnings forecast exclusions") OR ("EBIT") OR ("EBITA") OR (*

"EBITDA") OR ("EBT") OR ("GAAP-adjusted") OR ("Non-GAAP") OR ("Non-generally Accepted Accounting Principles") OR ("One-time gains") OR ("Proforma") OR ("Pro forma") OR ("Reg. G") OR ("Regulation G") OR ("Street earnings") OR ("Street exclusions"))).

[c] Search for the phrases only 'in the title of the article', since a search in the abstract or keywords is not possible at *Google Scholar* and a search 'anywhere in the article' would have yielded too many thematically inappropriate search results that do not substantively focus on APMs. Moreover, since a search without restricting the subject resulted in too many irrelevant search results that do not substantively focus on APMs, the search query was supplemented using relevant keywords from the subject area of accounting. In addition to the keywords listed in *Tab. 1*, at least one of the following keywords must be contained in the article: 'financial', 'accounting', 'performance', 'income' or 'management'. Accordingly, the *Google Scholar* search string used is documented as follows: *allintitle: ("Adjusted EPS" OR "Adjusted earnings" OR "Alternative earnings" OR "Alternative performance measures" OR "Earnings before" OR "Earnings exclusions" OR "Earnings forecast exclusions" OR "EBIT" OR "EBITA" OR "EBITDA" OR "EBT" OR "GAAP-adjusted" OR "Non-GAAP" OR "Non-Generally Accepted Accounting Principles" OR "One-time gains" OR "Proforma" OR "Pro forma" OR "Reg. G" OR "Regulation G" OR "Street earnings" OR "Street exclusions") AND ("financial" OR "accounting" OR "performance" OR "income" OR "management").*

[d] Search for the phrases only in the fields 'Title' (TI), 'Abstract or Author-Supplied Abstract' (AB) and 'Author-Supplied Keywords' (KW). Furthermore, the search was conducted exclusively in the category 'Academic Journals', since a search in all categories (e.g., 'trade publications', 'magazines', 'newspapers', 'market research reports', 'industry profiles', 'country reports') yielded too many thematically inappropriate search results that do not substantively focus on APMs. Accordingly, the *EBSCO Business Source Premier* search string used is documented as follows: *TI ("Adjusted EPS" OR "Adjusted earnings" OR "Alternative earnings" OR "Alternative performance measures" OR "Earnings before" OR "Earnings exclusions" OR "Earnings forecast exclusions" OR "EBIT" OR "EBITA" OR "EBITDA" OR "EBT" OR "GAAP-adjusted" OR "Non-GAAP" OR "Non-Generally Accepted Accounting Principles" OR "One-time gains" OR "Proforma" OR "Pro forma" OR "Reg. G" OR "Regulation G" OR "Street earnings" OR "Street exclusions")) OR AB ("Adjusted EPS" OR "Adjusted earnings" OR "Alternative earnings" OR "Alternative performance measures" OR "Earnings before" OR "Earnings exclusions" OR "Earnings forecast exclusions" OR "EBIT" OR "EBITA" OR "EBITDA" OR "EBT" OR "GAAP-adjusted" OR "Non-GAAP" OR "Non-Generally Accepted Accounting Principles" OR "One-time gains" OR "Proforma" OR "Pro forma" OR "Reg. G" OR "Regulation G" OR "Street earnings" OR "Street exclusions") OR KW ("Adjusted EPS" OR "Adjusted earnings" OR "Alternative earnings" OR "Alternative performance measures" OR "Earnings before" OR "Earnings exclusions" OR "Earnings forecast exclusions" OR "EBIT" OR "EBITA" OR "EBITDA" OR "EBT" OR "GAAP-adjusted" OR "Non-GAAP" OR "Non-Generally Accepted Accounting Principles" OR "One-time gains" OR "Proforma" OR "Pro forma" OR "Reg. G" OR "Regulation G" OR "Street earnings" OR "Street exclusions").*

[e] Search for the phrases only in the fields 'Title' and 'Topic', since a search in the abstract and in the keywords is not possible in the *ISI Web of Science* database and a search in all fields would have yielded too many thematically inappropriate search results that do not substantively focus on APMs. Furthermore, the search was conducted exclusively in the categories ('Web of Science Categories') 'Business Finance', 'Economics', 'Management' and 'Business', since a search in all Web of Science Categories yielded too many thematically inappropriate search results that do not substantively focus on APMs. Accordingly, the *ISI Web of Science* search string used is documented as follows: *TITLE: ("Adjusted EPS" OR "Adjusted earnings" OR "Alternative earnings" OR "Alternative performance measures" OR "Earnings before" OR "Earnings exclusions" OR "Earnings forecast exclusions" OR "EBIT" OR "EBITA" OR "EBITDA" OR "EBT" OR "GAAP-adjusted" OR "Non-GAAP" OR "Non-Generally Accepted Accounting Principles" OR "One-time gains" OR "Proforma" OR "Pro forma" OR "Reg. G" OR "Regulation G" OR "Street earnings" OR "Street exclusions") OR TOPIC: ("Adjusted EPS" OR "Adjusted earnings" OR "Alternative earnings" OR "Alternative performance measures" OR "Earnings before" OR "Earnings exclusions" OR "Earnings forecast exclusions" OR "EBIT" OR "EBITA" OR "EBITDA" OR "EBT" OR "GAAP-adjusted" OR "Non-GAAP" OR "Non-Generally Accepted Accounting Principles" OR "One-time gains" OR "Proforma" OR "Pro forma" OR "Reg. G" OR "Regulation G" OR "Street earnings" OR "Street exclusions"). Refined by: WEB OF SCIENCE CATEGORIES: (BUSINESS FINANCE OR ECONOMICS OR MANAGEMENT OR BUSINESS). Timespan: All years. Indexes: SCI-EXPANDED, SSCI, A&HCI, CPCI-S, CPCI-SSH, BKCI-S, BKCI-SSH, ESCI, CCR-EXPANDED, IC.*

Annex 3: Composition of the sample

Abarbanell, Jeffery S., and Reuven Lehavy. 2007. Letting the "Tail Wag the Dog": The Debate over GAAP versus Street Earnings Revisited. *Contemporary Accounting Research* 24: 675-723.

Abdel-Meguid, Ahmed, Jared N. Jennings, Kari Joseph Olsen, and Mark T. Soliman. 2021. The Impact of the CEO's Personal Narcissism on Non-GAAP Earnings. *Accounting Review* 96: 1-25.

Adams, Mollie T., and Michele D. Meckfessel. 2021. Are all non-GAAP disclosures created equal? *Business Horizons* 64: 7-18.

Afterman, Allan B. 2015. Non-GAAP Performance Measures – Virtue or Vice? *CPA Journal* 85(10): 48-49.

Aimino, P., G. A. Acunzo, M. Vavassori, and E. Abate. 2020. Internal Controls Procedures and External Auditor Involvement in Presence of Non-GAAP Measures. In *Reporting Non-GAAP Financial Measures*, ed. Nicola Moscariello and Michele Pizzo, 156-171. Newcastle: Cambridge Scholars Publishing.

Aimino, P., G. A. Acunzo, M. Vavassori, and E. Abate. 2020. The 'Big Four's Literature on the Non-GAAP Issue. In *Reporting Non-GAAP Financial Measures*, ed. Nicola Moscariello and Michele Pizzo, 140-155. Newcastle: Cambridge Scholars Publishing.

Akindayomi, Akinloye. 2012. The Relationship Between Executive Pay and Alternative Earnings Measures. *Academy of Accounting and Financial Studies Journal* 16: 41-58.

Al-Laban, Dhyaa Abdulrazaq Abduljabar, Abbas Naser Saadoon Almshabbk, and Wissam Abdulkadhum Abdulridha. 2018. The Role of Dividend Payout in the Determination of the Relationship Between Earnings Before Interest and Tax Cash Flow From Operations, Capital Expenditures and Firm Sustainable Cash Flows: A Case Of Non-Financial Firms. *The Journal of Social Sciences Research* 4: 382-390 (Special Issue 5).

Albring, Susan M., Maria T. Cabán-Garcia, and Jacqueline L. Reck. 2010. The value relevance of a non-GAAP performance metric to the capital markets. *Review of Accounting and Finance* 9: 264-284.

Alcalde, Adriano, Luiz Paulo Lopes Fávero, and Renata Turola Takamatsu. 2013. EBITDA margin in brazilian companies – Variance decomposition and hierarchical effects. *Contaduría y Administración* 58: 197-220.

Allee, Kristian D., Nilabhra Bhattacharya, Ervin L. Black, and Theodore E. Christensen. 2007. Pro forma disclosure and investor sophistication: External validation of experimental evidence using archival data. *Accounting, Organizations and Society* 32: 201-222.

Anders, Susan B. 2017. A Non-GAAP Reporting Sampler. *CPA Journal* 87(8): 72-73.

Anderson, Spencer B., Jessen L. Hobson, and Ryan D. Sommerfeldt. 2021. Auditing Non-GAAP Measures: Signaling More Than Intended. *Contemporary Accounting Research*. https://doi.org/10.1111/1911-3846.12724.

Andersson, Patric, and Niclas Hellman. 2007. Does Pro Forma Reporting Bias Analyst Forecasts? *European Accounting Review* 16: 277-298.

Andrade, Jefferson Pereira, and Wenner Glaucio Lopes Lucena. 2017. Value Relevance: A Study of Initial Public Offerings (IPOs). *Systems & Management* 12: 205-214.

Anilowski Cain, Carol, Kalin S. Kolev, and Sarah McVay. 2020. Detecting Opportunistic Special Items. *Management Science* 66: 2099-2119.

Arena, Claudia, Simona Catuogno, and Nicola Moscariello. 2021. The unusual debate on non-GAAP reporting in the current standard practice. The lens of corporate governance. *Journal of Management & Governance* 25: 655-684.

Arnold, Jerry L., and Joseph William Duggan. 2002. Making Pro Forma Information More Useful. *Financial Executive* 18(3): 38-41.

Aselta, James, and Russell Paul Engel. 2018. Tesla, Non-GAAP Financial Measures, and the Securities & Exchange Commission's Challenge: A Case Study Illustration. *The Accounting Educators' Journal* 28: 235-255.

Asper, Seanna, Chris McCoy, and Gary K. Taylor. 2019. The Expanding Use of Non-GAAP Financial Measures. *CPA Journal* 89(7): 28-31.

Aubert, François, and Gary Grudnitski. 2014. The role of reconciliation quality in limiting mispricing of non-GAAP earnings announcements by EURO STOXX firms. *Advances in Accounting* 30: 154-167.

Aubert, François, and Gary Grudnitski. 2020. Mispricing of Non-GAAP Earnings Disclosures by European Firms: A Fama and French Three-Factor Model Approach. In *Reporting Non-GAAP Financial Measures*, ed. Nicola Moscariello and Michele Pizzo, 224-241. Newcastle: Cambridge Scholars Publishing.

Auer, Katharina, and Daniela Maresch. 2006. Die Kennzahl EBITDA (Earnings before Interest, Tax, Depreciation and Amortisation) im IFRS-Konzernabschluss. *Zeitschrift für Recht und Rechnungswesen* 16: 144-148.

Badertscher, Brad A. 2011. Overvaluation and the Choice of Alternative Earnings Management Mechanisms. *Accounting Review* 86: 1491-1518.

Baik, Bok, Bruce K. Billings, and Richard M. Morton. 2008. Reliability and Transparency of Non-GAAP Disclosures by Real Estate Investment Trusts (REITs). *Accounting Review* 83: 271-301.

Baik, Bok, David B. Farber, and Kathy Petroni. 2009. Analysts' Incentives and Street Earnings. *Journal of Accounting Research* 47: 45-69.

Bansal, Naresh, Ananth Seetharaman, and Xu Wang. 2013. Managerial risk-taking incentives and non-GAAP earnings disclosures. *Journal of Contemporary Accounting & Economics* 9: 100-121.

Barlas, Stephen. 2003. SEC Proposed New Regulation G on Non-GAAP Financials. *Strategic Finance* 84(7): 3.

Barone, E., and C. Teodori. 2020. The Standard Setters' Approach to the Non-GAAP Measures. In *Reporting Non-GAAP Financial Measures*, ed. Nicola Moscariello and Michele Pizzo, 94-116. Newcastle: Cambridge Scholars Publishing.

Barth, Mary E., Ian D. Gow, and Daniel J. Taylor. 2012. Why do pro forma and Street earnings not reflect changes in GAAP? – Evidence from SFAS 123R. *Review of Accounting Studies* 17: 526-562.

Batta, George, and Volkan Muslu. 2017. Credit Rating Agency and Equity Analysts' Adjustments to GAAP Earnings. *Contemporary Accounting Research* 34: 783-817.

Baumker, Michael, Philip Biggs, Sarah McVay, and Jeremy Pierce. 2014. The Disclosure of Non-GAAP Earnings Following Regulation G: An Analysis of Transitory Gains. *Accounting Horizons* 28: 77-92.

Begley, Joy, Sandra Chamberlain, and Qiang Cheng. 2011. *The Valuation of Pro-Forma Free Cash Flows in an IPO Setting: The Case of Canadian Income Trusts*. Working Paper. University of British Columbia, Vancouver and University of Wisconsin, Madison. May 2011. https://dx.doi.org/10.2139/ssrn.1911774. Accessed 12 December 2020.

Bentley, Jeremiah W., Theodore E. Christensen, Kurt H. Gee, and Benjamin C. Whipple. 2018. Disentangling Managers' and Analysts' Non-GAAP Reporting. *Journal of Accounting Research* 56: 1039-1081.

Berger, Philip G. 2005. Discussion of "Are Investors Misled by 'Pro Forma' Earnings?". *Contemporary Accounting Research* 22: 965-976.

Bernstein, Drew. 2019. Has Non-GAAP Reporting Become an Accounting Chasm? *CFO Magazine* 35(12): 20-21.

Bhattacharya, Nilabhra, Ervin L. Black, Theodore E. Christensen, and Chad R. Larson. 2003. Assessing the relative informativeness and permanence of pro forma earnings and GAAP operating earnings. *Journal of Accounting and Economics* 36: 285-319.

Bhattacharya, Nilabhra, Ervin L. Black, Theodore E. Christensen, and Richard D. Mergenthaler. 2004. Empirical Evidence on Recent Trends in Pro Forma Reporting. *Accounting Horizons* 18: 27-43.

Bhattacharya, Nilabhra, Ervin L. Black, Theodore E. Christensen, and Richard D. Mergenthaler. 2007. Who Trades on Pro Forma Earnings Information? *Accounting Review* 82: 581-619.

Bhattacharya, Nilabhra, Theodore E. Christensen, Qunfeng Liao, and Bo Quyang. 2021. Can short sellers constrain aggressive non-GAAP reporting? *Review of Accounting Studies*. https://doi.org/10.1007/s11142-021-09621-9.

Bieker, Marcus, and Johannes Julius Moser. 2011. Earnings Before What? – Zur babylonischen Sprachverwirrung in deutschen Geschäftsberichten. *Praxis der internationalen Rechnungslegung* 7: 163-170.

Bierstaker, James L., Thomas F. Monahan, and Michael F. Peters. 2013. Going Concern Designations and GAAP versus Non-GAAP Earnings Metrics. *Issues in Accounting Education* 28: 77-92.

Binder, Christoph, and Nils Högsdal. 2017. EBIT & Co. *Controlling & Management Review* 61(2): 58-61.

Bini, Laura, Francesco Giunta, and Rebecca Miccini. 2020. The Reliability of Non-GAAP Disclosure in Europe: An Examination of Presentational Aspects. In *Reporting Non-GAAP Financial Measures*, ed. Nicola Moscariello and Michele Pizzo, 242-286. Newcastle: Cambridge Scholars Publishing.

Bishal, B. C., and Bo Liu. 2021. Non-GAAP measure disclosure and insider trading incentives in high-tech IPO firms. *Accounting Research Journal*. https://doi.org/10.1108/ARJ-01-2021-0016.

Blab, Daniel, and Eugen Hubertus Leopold Turi. 2018. Alternative Performance Measures – Analyse zur Anwendungspraxis im MDAX (Teil 1). *Zeitschrift für internationale und kapitalmarktorientierte Rechnungslegung* 18: 15-21.

Blab, Daniel, and Eugen Hubertus Leopold Turi. 2018. Alternative Performance Measures – Analyse zur Anwendungspraxis im MDAX (Teil 2). *Zeitschrift für internationale und kapitalmarktorientierte Rechnungslegung* 18: 72-77.

Black, Dirk E., and Theodore E. Christensen. 2009. US Managers' Use of 'Pro Forma' Adjustments to Meet Strategic Earnings Targets. *Journal of Business Finance & Accounting* 36: 297-326.

Black, Dirk E., and Theodore E. Christensen. 2018. Policy Implications of Research on Non-GAAP Reporting. *Research in Accounting Regulation* 30: 1-7.

Black, Dirk E., Ervin L. Black, Theodore E. Christensen, and Kurt H. Gee. 2021b. CEO Pay Components and Aggressive Non-GAAP Earnings Disclosure. *Journal of Accounting, Auditing & Finance*. https://doi.org/10.1177%2F0148558X21989907.

Black, Dirk E., Ervin L. Black, Theodore E. Christensen, and Kurt H. Gee. 2021. Comparing Non-GAAP EPS in Earnings Announcements and Proxy Statements. *Management Science*. https://doi.org/10.1287/mnsc.2020.3928.

Black, Dirk E., Ervin L. Black, Theodore E. Christensen, and William G. Heninger. 2012. Has the Regulation of Pro Forma Reporting in the US Changed Investors' Perceptions of Pro Forma Earnings Disclosures? *Journal of Business Finance & Accounting* 39: 876-904.

Black, Dirk E., Theodore E. Christensen, Jack T. Ciesielski, and Benjamin C. Whipple. 2018. Non-GAAP reporting: Evidence from academia and current practice. *Journal of Business Finance & Accounting* 45: 259-294.

Black, Dirk E., Theodore E. Christensen, Jack T. Ciesielski, and Benjamin C. Whipple. 2021a. Non-GAAP Earnings: A Consistency and Comparability Crisis? *Contemporary Accounting Research* 38: 1712-1747.

Black, Ervin L. 2016. The Ethical Reporting of Non-GAAP Performance Measures. *Revista Contabilidade & Finanças* 27: 7-11.

Black, Ervin L., Theodore E. Christensen, Paraskevi Vicky Kiosse, and Thomas D. Steffen. 2017a. Has the Regulation of Non-GAAP Disclosures Influenced Managers' Use of Aggressive Earnings Exclusions? *Journal of Accounting, Auditing & Finance* 32: 209-240.

Black, Ervin L., Theodore E. Christensen, Paraskevi Vicky Kiosse, and Thomas D. Steffen. 2019. *The Influence of Manager-Analyst Interactions on Street Earnings: Evidence from Conference Calls and Excluded Analysts*. Working Paper. University of Oklahoma, Norman et al. February 2019. https://dx.doi.org/10.2139/ssrn.2992618. Accessed 17 January 2022.

Black, Ervin L., Theodore E. Christensen, T. Taylor Joo, and Roy Schmardebeck. 2017b. The Relation Between Earnings Management and Non-GAAP Reporting. *Contemporary Accounting Research* 34: 750-782.

Bloom, Robert, and David Schirm. 2003. SEC Regulations G, S-B, and S-K: Reporting Non-GAAP Financial Measures. *CPA Journal* 73(12): 10.

Bochkay, Khrystyna, Stan Markov, Musa Subasi, and Eric Weisbrod. 2021. *The Acquisition and Integration of Street Earnings: Evidence from a Natural Experiment*. Working Paper. University of Miami, Coral Gables et al. June 2021. https://dx.doi.org/10.2139/ssrn.3219449. Accessed 17 January 2022.

Bond, David, Robert Czernkowski, Yong-Suk Lee, and Anna Loyeung. 2017. Market reaction to non-GAAP earnings around SEC regulation. *Journal of Contemporary Accounting & Economics* 13: 193-208.

Bouwens, Jan, Ties de Kok, and Arnt Verriest. 2019. The Prevalence and Validity of EBITDA as a Performance Measure. *Comptabilité Contrôle Audit* 25: 55-105.

Bowen, Robert M., Angela K. Davis, and Dawn A. Matsumoto. 2005. Emphasis on Pro Forma versus GAAP Earnings in Quarterly Press Releases: Determinants, SEC Intervention, and Market Reactions. *Accounting Review* 80: 1011-1038.

Boyer, Benoit, Ralph Lim, and Bridget Lyons. 2016. A Case Study in the Use and Potential Misuse of Non-GAAP Financial Measures. *Journal of Applied Business and Economics* 18: 117-126.

Bradshaw, Mark T. 2003. A discussion of 'Assessing the relative informativeness and permanence of pro forma earnings and GAAP operating earnings'. *Journal of Accounting and Economics* 36: 321-335.

Bradshaw, Mark T. 2011. A discussion of "Do managers use earnings guidance to influence street earnings exclusions?". *Review of Accounting Studies* 16: 528-538.

Bradshaw, Mark T., and Mark Soliman. 2007. Discussion of "Letting the 'Tail Wag the Dog': The Debate over GAAP versus Street Earnings Revisited". *Contemporary Accounting Research* 24: 725-739.

Bradshaw, Mark T., and Richard G. Sloan. 2002. GAAP versus The Street: An Empirical Assessment of Two Alternative Definitions of Earnings. *Journal of Accounting Research* 40: 41-66.

Bradshaw, Mark T., Theodore E. Christensen, Kurt H. Gee, and Benjamin C. Whipple. 2018. Analysts' GAAP earnings forecasts and their implications for accounting research. *Journal of Accounting and Economics* 66: 46-66.

Branch, William J., Paul W. Farris, and Mark E. Haskins. 2011. Pay for Performance: Keep it Simple and Value-Focused. *Compensation & Benefits Review* 43: 82-91.

Bratten, Brian, Stephannie Larocque, and Teri Yohn. 2021. *Filling in the GAAPs in Individual Analysts' Street Earnings Forecasts*. Working Paper. University of Kentucky, Lexington et al. May 2021. https://dx.doi.org/10.2139/ssrn.3074701. Accessed 17 January 2022.

Bratten, Brian, Stephannie Larocque, and Teri Yohn. 2021. *On the Informativeness of Unexpected Exclusions from Street Earnings*. Working Paper. University of Kentucky, Lexington et al. September 2021. https://dx.doi.org/10.2139/ssrn.3470390. Accessed 17 January 2022.

Bricker, Wesley, and Marc Siegel. 2016. Listening to Users on Transition Issues, Non-GAAP Measures, and Disclosures – Remarks from the SEC and FASB. *CPA Journal* 86(7): 28-33.

Brockbank, Bryan G. 2017. *Do Non-GAAP Exclusions Impact the Extent to Which Current Returns Reflect Future Earnings Information?* Dissertation University of Oklahoma, Norman.

Brockman, Christopher M., and Judson W. Russell. 2012. EBITDA: use it ... or lose it? *International Journal of Business, Accounting & Finance* 6: 84.

Brody, Rochard D., and Robert McDonald. 2004. The Next Scandal: The Undisciplined Use of Pro Forma Financial Statements. *American Business Review* 22: 34-38.

Brouwer, Arjan, and Benton E. Gup. 2010. EBITDA – Down but Not Out. In *The Valuation Handbook*, ed. Rawley Thomas and Benton E. Gup, 525-543. Hoboken: John Wiley & Sons.

Brouwer, Arjan. 2013. 'Profit, performance, perception': a research into the use of alternative performance measures in the European Union. Dissertation University of Amsterdam, Amsterdam.

Brown, Lawrence D., and Kumar Sivakumar. 2003. Comparing the Value Relevance of Two Operating Income Measures. *Review of Accounting Studies* 8: 561-572.

Brown, Nerissa C., and Theodore E. Christensen. 2014. The quality of street cash flow from operations. *Review of Accounting Studies* 19: 913-954.

Brown, Nerissa C., Adrienna A. Huffman, and Shira Cohen. 2020. *Accounting Reporting Complexity and Non-GAAP Earnings Disclosure*. Working Paper. University of Illinois at Urbana-Champaign, Illinois et al. October 2020. https://dx.doi.org/10.2139/ssrn.3224798. Accessed 17 January 2022.

Brown, Nerissa C., Theodore E. Christensen, and W. Brooke Elliott. 2012a. The Timing of Quarterly 'Pro Forma' Earnings Announcements. *Journal of Business Finance & Accounting* 39: 315-359.

Brown, Nerissa C., Theodore E. Christensen, W. Brooke Elliott, and Richard D. Mergenthaler. 2012b. Investor Sentiment and Pro Forma Earnings Disclosures. *Journal of Accounting Research* 50: 1-40.

Brown, Nerissa. 2020. Going Public: The benefits and pitfalls of non-GAAP metrics. *Journal of Education and Research in Accounting* 14: 145-157.

Bryan, Stephen, and Steven Lilien. 2004. Managed Disclosure and Pro Forma Earnings. *CPA Journal* 74(3): 40-45.

Bryant, Lisa, Steven L. Henning, and Wayne H. Shaw. 2010. Alternative Earnings Measures, Key Performance Indicators and Firm Value in the IT Professional Services Sector. *Journal of Forensic & Investigative Accounting* 2: 1-33.

Byrnes, Nanette, and Tom Lowry. 2002. A different yardstick for cable. *BusinessWeek* 74(3797): 38.

Cameron, Robyn, Majella Percy, and Peta Stevenson-Clarke. 2012. Do Large Australian Companies Emphasise Non-GAAP Financial Measures over Statutory Net Profit (GAAP) in Annual Reports? *JASSA* 3(1): 19-25.

Campbell, Linda, and Dennis M. López. 2010. Small cap financial reporting: Determinants of emphasis and placement of non-GAAP disclosures. *Research in Accounting Regulation* 22: 114-120.

Campbell, Linda, and Marshall K. Pitman. 2009. Post-regulation G findings. *Research in Accounting Regulation* 21: 100-106.

Carvajal, Matriela, David H. Lont, and Tom Scott. 2021. Non-GAAP Earnings Disclosure Trends in New Zealand. *Australian Accounting Review*. https://doi.org/10.1111/auar.12358.

Cassis, John. 2002. Cash Flow or EBITDA? Can't We Have Both? *The RMA Journal* 85(4): 26-34.

Catuogno, Simona, and Claudia Arena. 2020. Determinants and Consequences of Non-GAAP Disclosure: A Review of the Literature. In *Reporting Non-GAAP Financial Measures*, ed. Nicola Moscariello and Michele Pizzo, 38-65. Newcastle: Cambridge Scholars Publishing.

Cencioni, L., P. Fattorusso, and R. Nardi. 2020. Non-GAAP Measures: The Case of the Eni Group. In *Reporting Non-GAAP Financial Measures*, ed. Nicola Moscariello and Michele Pizzo, 362-383. Newcastle: Cambridge Scholars Publishing.

CFA Society. 2015. *Non-IFRS Earnings and Alternative Performance Measures: Ensuring A Level Playing Field*. https://www.cfauk.org/-/media/files/pdf/pdf/5-professionalism/3-research-and-position-papers/non-ifrs-earnings-and-alternative-performance-measures.pdf. Accessed 17 January 2022.

Charitou, Andreas, Nikolaos Floropoulos, Irene Karamanou, and George Loizides. 2018. Non-GAAP Earnings Disclosures on the Face of the Income Statement by UK Firms: The Effect on Market Liquidity. *The International Journal of Accounting* 53: 183-202.

Chen, Chih-Ying. 2010. Do analysts and investors fully understand the persistence of the items excluded from Street earnings? *Review of Accounting Studies* 15: 32-69.

Chen, Han-Chung, Yen-Jung Lee, Sheng-Yi Lo, and Yong Yu. 2021. Qualitative characteristics of non-GAAP disclosures and non-GAAP earnings quality. *Journal of Accounting and Economics*. https://doi.org/10.1016/j.jacceco.2021.101402.

Chen, Jason V., Kurt H. Gee, and Jed J. Neilson. 2021. Disclosure Prominence and the Quality of Non-GAAP Earnings. *Journal of Accounting Research* 59: 163-213.

Chen, Long, Gopal Krishnan, and Mikhail Pevzner. 2012. Pro forma disclosures, audit fees, and auditor resignations. *Journal of Accounting and Public Policy* 31: 237-257.

Chen, Wei. 2009. Usefulness of Pro Forma Accounting Information Mandated in IPO Prospectuses: Its Association with IPO Pricing and Subsequent Firm Performance. Dissertation Polytechnic University Hong Kong, Hong Kong.

Chen, Xia, Xuejun Jiang, Louise Yi Lu, and Yangxin Yu. 2021. Local political corruption and Firm's non-GAAP reporting. *Journal of Corporate Finance*. https://doi.org/10.1016/j.jcorpfin.2021.102071.

Chen, Xia. 2019. Managerial sentiment and non-GAAP earnings disclosure: evidence from terrorist attacks. *Asia-Pacific Journal of Accounting & Economics*. https://doi.org/10.1080/16081625.2019.1673189.

Chen, Yu-An, Ann F. Medinets, and Dan Palmon. 2021. Disclosure regulations work: The case of regulation G. *Review of Quantitative Finance and Accounting*. https://doi.org/10.1007/s11156-021-01017-9.

Cheng, C. S. Agnes. 2016. Discussion of 'IFRS non-GAAP earnings disclosures and fair value measurement'. *Accounting & Finance* 56: 99-112.

Cheng, Chia-Hsin, Robin K. Chou, and Shin-Rong Shiah-Hou. 2012. Does Regulation G Change the Way that Analysts Use Pro Forma Earnings Information? Conference Paper. Asian Finance Association 2012 International Conference, Taipei.

Cheng, Yun. 2017. Managerial Reputation and the Quality of Non-GAAP Earnings Disclosures. *Journal of Accounting and Finance* 17: 117-134.

Chidley, Joe. 2003. Pro-formatic, baby! *Canadian Business* 76(3): 4.

Choi, Young-Soo, and Steven Young. 2015. Transitory earnings components and the two faces of non-generally accepted accounting principles earnings. *Accounting & Finance* 55: 75-103.

Choi, Young-Soo, Stephen Lin, Martin Walker, and Steven Young. 2007. Disagreement over the persistence of earnings components: evidence on the properties of management-specific adjustments to GAAP earnings. *Review of Accounting Studies* 12: 595-622.

Choi, Young-Soo. 2015. First voluntary disclosure: is it less opportunistic? *Asia-Pacific Journal of Accounting & Economics* 22: 347-367.

Christensen, Theodore E. 2007. Discussion of "another look at GAAP versus the Street: an empirical assessment of measurement error bias". *Review of Accounting Studies* 12: 305-321.

Christensen, Theodore E. 2007. Discussion of "Letting the 'Tail Wag the Dog': The Debate over GAAP versus Street Earnings Revisited". *Contemporary Accounting Research* 24: 741-762.

Christensen, Theodore E. 2012. Discussion of "Why do pro forma and street earnings not reflect changes in GAAP? Evidence from SFAS 123R". *Review of Accounting Studies* 17: 563-571.

Christensen, Theodore E., Enrique Gomez, Matthew Ma, and Jing Pan. 2021. Analysts' role in shaping non-GAAP reporting: evidence from a natural experiment. *Review of Accounting Studies* 26: 172-217.

Christensen, Theodore E., Hang Pei, Spencer R. Pierce, and Liang Tan. 2019. Non-GAAP reporting following debt covenant violations. *Review of Accounting Studies* 24: 629-664.

Christensen, Theodore E., Kenneth J. Merkley, Jennifer Wu Tucker, and Shankar Venkataraman. 2011. Do managers use earnings guidance to influence street earnings exclusions? *Review of Accounting Studies* 16: 501-527.

Christensen, Theodore E., Michael S. Drake, and Jacob R. Thornock. 2014. Optimistic Reporting and Pessimistic Investing: Do Pro Forma Earnings Disclosures Attract Short Sellers? *Contemporary Accounting Research* 31: 67-102.

Ciccone, Stephen John. 2002. GAAP versus Street Earnings: Making Earnings Look Higher and Smoother. Working Paper. University of New Hampshire, New Hampshire. August 2002. https://dx.doi.org/10.2139/ssrn.319320. Accessed 17 January 2022.

Ciesielski, Jack T., and Elaine Henry. 2017. Accounting's Tower of Babel: Key Considerations in Assessing Non-GAAP Earnings. *Financial Analysts Journal* 73: 34-50.

Clarke, David, Paul Sassalos, and Kelly Schmitt. 2003. The SEC's New Disclosure Requirements for Non-GAAP Financial Information. *Insights: The Corporate and Securities Law Advisor* 17(4): 2-8.

Clinch, Greg, Ann Tarca, and Marvin Wee. 2018. *The value relevance of IFRS earnings totals and subtotals and non-GAAP performance measures*. Working Paper. University of Melbourne, Melbourne. March 2018. https://dx.doi.org/10.2139/ssrn.3178567. Accessed 17 January 2022.

Cogliati, M. A., and L. Noviello. 2020. Non-GAAP Disclosure by Intesa Sanpaolo Banking Group. In *Reporting Non-GAAP Financial Measures*, ed. Nicola Moscariello and Michele Pizzo, 384-405. Newcastle: Cambridge Scholars Publishing.

Cohen, Daniel A., Rebecca N. Hann, and Maria Ogneva. 2007. Another look at GAAP versus the Street: an empirical assessment of measurement error bias. *Review of Accounting Studies* 12: 271-303.

Collin, S. Y. 2020. Regulation of Non-GAAP Measures: To What Extent Should They Be Audited? In *Reporting Non-GAAP Financial Measures*, ed. Nicola Moscariello and Michele Pizzo, 172-191. Newcastle: Cambridge Scholars Publishing.

Collins, Daniel W., Oliver Zhen Li, and Hong Xie. 2009. What drives the increased informativeness of earnings announcements over time? *Review of Accounting Studies* 14: 1-30.

Cormier, Denis, Pascale Lapointe-Antunes, and Michel Magnan. 2011. Revisiting the Relevance and Reliability of Non-GAAP Reporting: The Case of the Income Trusts. *Contemporary Accounting Research* 28: 1585-1609.

Cormier, Denis, Samira Demaria, and Michel Magnan. 2017. Beyond earnings: do EBITDA reporting and governance matter for market participants? *Managerial Finance* 43: 193-211.

Cornell, Bradford, and Wayne R. Landsman. 2003. Accounting Valuation: Is Earnings Quality an Issue? *Financial Analysts Journal* 59: 20-28.

Coté, Douglas E., and Rong Qi. 2005. Honest EPS: a measure of GAAP earnings relative to pro forma earnings. *International Journal of Managerial Finance* 1: 25-35.

Curtis, Asher B., Sarah E. McVay, and Benjamin C. Whipple. 2014. The Disclosure of Non-GAAP Earnings Information in the Presence of Transitory Gains. *Accounting Review* 89: 933-958.

Curtis, Asher, Valerie Li, and Paige H. Patrick. 2021. The use of adjusted earnings in performance evaluation. *Review of Accounting Studies* 26: 1290-1322.

Dang, Suting. 2017. *Research on Application of Non-GAAP Financial Measures to Overseas Listed Companies*. Conference Paper. 3rd International Conference on Management Science and Innovative Education (MSIE 2017). http://doi.org/10.12783/dtssehs/msie2017/15438. Accessed 17 January 2022.

Davern, Michael, Nikole Gyles, Dean Hanlon, and Matthew Pinnuck. 2018. *Decision-Usefulness in Financial Reports*. Research Report No. 2: Relevance of Alternative Performance Measures and Non-Financial Information for Investor Decision Making in Australia. University Melbourne, Melbourne. https://researchmgt.monash.edu/ws/portalfiles/portal/282406038/281980837_oa.pdf. Accessed 17 January 2022.

Davern, Michael, Nikole Gyles, Dean Hanlon, and Matthew Pinnuck. 2019. Is Financial Reporting Still Useful? – Australian Evidence. *Abacus* 55: 237-272.

Dessai, Hrishikesh. 2020. *Essays on the influence of accounting regulation on non-GAAP reporting*. Dissertation University of Melbourne, Melbourne.

Di Fabio, Constanza, and Elisa Roncagliolo. 2020. Security Regulators' Requirements on Non-GAAP Disclosure. In *Reporting Non-GAAP Financial Measures*, ed. Nicola Moscariello and Michele Pizzo, 68-93. Newcastle: Cambridge Scholars Publishing.

Dilla, William N., Diane J. Janvrin, and Cynthia Jeffrey. 2013. The Impact of Graphical Displays of Pro Forma Earnings Information on Professional and Nonprofessional Investors' Earnings Judgments. *Behavioral Research in Accounting* 25: 37-60.

Dilla, William N., Diane J. Janvrin, and Cynthia Jeffrey. 2014. Pro forma accounting disclosures: The effect of reconciliations and financial reporting knowledge on nonprofessional investors' judgments. *Advances in Accounting* 30: 43-54.

Dinh, Tami, and Felix Thielemann. 2016. ESMA Guidelines on Alternative Performance Measures – Möglichkeiten und Herausforderungen bei der Darstellung von Pro-Forma-Kennzahlen. *Zeitschrift für Internationale Rechnungslegung* 11: 433-438.

Dinh, Tami, Wolfgang Schultze, and Chang Zhao. 2018. Alternative Performance Measures – Neueste Entwicklungen und Erkenntnisse aus der internationalen Forschung. *Zeitschrift für internationale und kapitalmarktorientierte Rechnungslegung* 18: 460-466.

Doyle, Jeffrey T., Jared N. Jennings, and Mark T. Soliman. 2013. Do managers define non-GAAP earnings to meet or beat analyst forecasts? *Journal of Accounting and Economics* 56: 40-56.

Doyle, Jeffrey T., Russell J. Lundholm, and Mark T. Soliman. 2003. The Predictive Value of Expenses Excluded from Pro Forma Earnings. *Review of Accounting Studies* 8: 145-174.

Dutta, Saurav K., Dennis H. Caplan, and David J. Marcinko. 2014. Growing Pains at Groupon. *Issues in Accounting Education* 29: 229-245.

Easton, Peter. 2003. Discussion of "The Predictive Value of Expenses Excluded from Pro Forma Earnings". *Review of Accounting Studies* 8: 175-183.

Elliott, W. Brooke. 2006. Are Investors Influenced by Pro Forma Emphasis and Reconciliations in Earnings Announcements? *Accounting Review* 81: 113-133.

Elshafie, Essam, Ai-Ru Yen, and Minna Yu. 2010. The association between pro forma earnings and earnings management. *Review of Accounting and Finance* 9: 139-155.

Elshafie, Essam, and Pervaiz Alam. 2006. *Are Investors Still Misled by Pro Forma Earnings Even after Financial Reporting Regulations?* Working Paper. University of Texas, Brownsville and Kent State University, Kent. September 2006. https://dx.doi.org/10.2139/ssrn.930868. Accessed 17 January 2022.

Eng, Li Li, and Thanyaluk Vichitsarawong. 2021. Comparing the usefulness of two profit subtotals: Operating income and earnings before interest and taxes. *Finance Research Letters*. https://doi.org/10.1016/j.frl.2021.102529.

Engle, Paul. 2010. Why care about EBITDA? *Industrial Engineer* 42(3): 22.

Entwistle, Gary M., Glenn D. Feltham, and Chima Mbagwu. 2005. The Voluntary Disclosure of Pro Forma Earnings: A U.S.-Canada Comparison. *Journal of International Accounting Research* 4: 1-23.

Entwistle, Gary M., Glenn D. Feltham, and Chima Mbagwu. 2006b. Financial Reporting Regulation and the Reporting of Pro Forma Earnings. *Accounting Horizons* 20: 39-55.

Entwistle, Gary M., Glenn D. Feltham, and Chima Mbagwu. 2010. The Value Relevance of Alternative Earnings Measures: A Comparison of Pro Forma, GAAP, and I/B/E/S Earnings. *Journal of Accounting, Auditing & Finance* 25: 261-288.

Entwistle, Gary M., Glenn D. Feltham, and Chima Mbagwu. 2012. Credibility Attributes and Investor Perceptions of Non-GAAP Earnings Exclusions. *Accounting Perspectives* 11: 229-257.

Entwistle, Gary, Glenn Feltham, and Chima Mbagwu. 2004. Proper pro forma reports. *CMA Management* 78(4):
26-29.

Entwistle, Gary, Glenn Feltham, and Chima Mbagwu. 2004. Voluntary Disclosure Practices: The Use of Pro Forma Reporting. *Journal of Applied Corporate Finance* 16: 73-80.

Entwistle, Gary, Glenn Feltham, and Chima Mbagwu. 2006a. Misleading Disclosure of Pro Forma Earnings: An Empirical Examination. *Journal of Business Ethics* 69: 355-372.

Epping, Lori L., and W. Mark Wilder. 2011. U.S.-Listed Foreign Firms' Non-GAAP Financial Performance Disclosure Behavior. *Journal of International Accounting Research* 10: 77-96.

Feng, Zhilan, Zhilu Lin, and Wentao Wu. 2020. CEO Influence on Funds from Operations (FFO) Adjustment for Real Estate Investment Trusts (REITs). *The Journal of Real Estate Finance and Economics*. https://doi.org/10.1007/s11146-020-09795-0.

Fera, P., R. Lombardi, and G. Ricciardi. 2020. Non-GAAP Disclosure by European Digital Companies: A Multiple-Case Analysis. In *Reporting Non-GAAP Financial Measures*, ed. Nicola Moscariello and Michele Pizzo, 335-359. Newcastle: Cambridge Scholars Publishing.

Fiechter, Peter. 2013. The Effects of Compensation and Board Quality on Non-GAAP Disclosures in Europe. *The International Journal of Accounting* 48: 318-323.

Fields, Thomas D., Srinivasan Rangan, and S. Ramu Thiagarajan. 1998. An Empirical Evaluation of the Usefulness of Non-GAAP Accounting Measures in the Real Estate Investment Trust Industry. *Review of Accounting Studies* 3: 103-130.

Fitzsimons, Adrian P., and Yeong C. Choi. 2002. SEC Addresses Pro Forma Financials, Accounting Policies, and Corporate Disclosures. *Bank Accounting & Finance* 15: 39-44.

Folster, André, Raphael Vinicius Weigert Camargo, and Ernesto Fernando Rodrigues Vicente. 2015. Management Earnings Forecast Disclosure: A Study on the Relationship between EBITDA Forecast and Financial Performance. *Revista de Gestão* 5: 108-124.

Fortin, Steve, Peng Liu, and Desmond Tsang. 2008. *SEC Intervention and Industry Guidance: The Effect on Non-GAAP Financial Disclosures*. Working Paper. McGill University, Montreal et al. December 2008. https://dx.doi.org/10.2139/ssrn.1326992. Accessed 17 January 2022.

Frankel, Richard, Sarah McVay, and Mark Soliman. 2011. Non-GAAP earnings and board independence. *Review of Accounting Studies* 16: 719-744.

Freches, Fabian, Melina Heilmann, and Christiane Pott. 2020. Management Performance Measures in der Neugestaltung des IAS 1 – Tatsächliche Verbesserung für die Darstellung von Non-GAAP Measures? *Zeitschrift für Internationale Rechnungslegung* 15: 513-519.

Frederickson, James R., and Jeffrey S. Miller. 2004. The Effects of Pro Forma Earnings Disclosures on Analysts' and Nonprofessional Investors' Equity Valuation Judgements. *Accounting Review* 79: 667-686.

Fridson, Martin S. 1998. EBITDA is not King. *Journal of Financial Statement Analysis* 3(3): 59-62.

Fülbier, Rolf Uwe, Klara E. Lösse, and Jens Kruse. 2021. IFRS, Non-GAAP-Measures und Non-Financials aus Sicht von Finanzanalysten. *Corporate Finance* 12: 1-7.

Gaber, Mohamed, Samy Garas, and Edward Lusk. 2020. Audit risk calibration: Extending the Non-GAAP SEC-Filter. *International Journal of Research in Business and Social Science* 9: 182-195.

Gao, Zhan, and Jiancheng Liu. 2018. *Comparability of Street Earnings and Its Economic Consequences*. Working Paper. Lancaster University, Lancashire and Monash University, Melbourne. July 2018. https://dx.doi.org/10.2139/ssrn.2878768. Accessed 17 January 2022.

Gehrig, Marco, Martin Hebertinger, and Lenka Sedlarik. 2020. Key Performance Indicators bei börsenkotierten Unternehmen – eine aktuelle Praxiserhebung. *Zeitschrift für Internationale Rechnungslegung* 15: 457-460.

Gordon, Michael S. 2019. *Issues with Public Disclosure of Non-GAAP Financial Metrics*. Working Paper. University of Maryland, Maryland. July 2019. https://dx.doi.org/10.2139/ssrn.3405661. Accessed 17 January 2022.

Gossage, Bobbie. 2004. Cranking Up the Earnings. *Inc. Magazine* 26(10): 54.

Grant, Julia, and Larry Parker. 2002. EBITDA! *Research in Accounting Regulation* 15: 205-211.

Greenberg, Herb. 1998. Ebitda: Never Trust Anything That You Can't Pronounce. *Fortune* 137(12): 192-194.

Greenberg, Herb. 2000. Alphabet Dupe: Why Ebitda Falls Short. *Fortune* 142(2): 240-241.

Grey, Colette, Konstantinos Stathopoulos, and Martin Walker. 2013. The impact of executive pay on the disclosure of alternative earnings per share figures. *International Review of Financial Analysis* 29: 227-236.

Griffin, Paul A., and David H. Lont. 2021. Evidence of an increasing trend in earnings surprises over the past two decades: The role of positive manager-initiated non-GAAP adjustments. *Journal of Business Finance & Accounting* 48: 1525-1559.

Gronewold, Ulfert, and Thorsten Sellhorn. 2009. Pro Forma Earnings. *Die Betriebswirtschaft* 69: 107-111.

Großmann, Birthe. 2007. *Die Pro-forma-Berichterstattung in Deutschland – Eine empirische Untersuchung zum Informationsgehalt und zur Bewertungsrelevanz von Pro-forma-Ergebnissen*. Frankfurt: Peter Lang Verlag.

Gu, Zhaoyang, and Ting Chen. 2004. Analysts' treatment of nonrecurring items in street earnings. *Journal of Accounting and Economics* 38: 129-170.

Guillamon-Saorin, Encarna, Helena Isidro, and Ana Marques. 2017. Impression Management and Non-GAAP Disclosure in Earnings Announcements. *Journal of Business Finance & Accounting* 44: 448-479.

Guillamon-Saorin, Encarna, Helena Isidro, and Ana Marques. 2020. Reporting of Alternative Performance Measures by European Firms. In *Reporting Non-GAAP Financial Measures*, ed. Nicola Moscariello and Michele Pizzo, 196-223. Newcastle: Cambridge Scholars Publishing.

Haaker, Andreas, and Jens Freiberg. 2011. Pro-Forma-Ergebnisse zur Anlegerinformation? *Praxis der internationalen Rechnungslegung* 7: 259-260.

Haaker, Andreas. 2019. EBITDA vor Sondereinflüssen im Akquisitionskontext. *Zeitschrift für Internationale Rechnungslegung* 14: 97-100.

Haaker, Andreas. 2019. ROCE statt EBIT nach Kapitalkosten als zentrale Berichtskennzahl – Eine Fallstudie zum Berichtskennzahlenmanagement von BASF. *Zeitschrift für Internationale Rechnungslegung* 14: 238-241.

Haaker, Andreas. 2020. EBITDA als Maß der Kapitaldienstfähigkeit? *Zeitschrift für Internationale Rechnungslegung* 15: 480-483.

Haaker, Andreas. 2020. Nachgehakt: Zur externen Ermittlung der suspendierten Berichtskennzahl EBIT nach Kapitalkosten – Eine weitere Fallstudie zum Berichtskennzahlenwechsel von BASF. *Zeitschrift für Internationale Rechnungslegung* 15: 267-270.

Haaker, Andreas. 2020. Zum Konzept des Eigentümergewinns (Buffett's owner earnings). *Zeitschrift für Internationale Rechnungslegung* 15: 15-17.

Haaker, Andreas. 2021. EBITDA-Ergebnisbeschönigungseffekt. *Zeitschrift für Internationale Rechnungslegung* 16(1): 13-17.

Haaker, Andreas. 2021. Ergebnistrendanalyse bei ambivalenter Entwicklung der Ergebnisgrößen – Eine Fallstudie am Beispiel des Bayer-Geschäftsberichts 2020. *Zeitschrift für Internationale Rechnungslegung* 16: 153-158.

Habib, Ahsan. 2010. Value relevance of alternative accounting performance measures: Australian evidence. *Accounting Research Journal* 23: 190-212.

Halsey, Bob, and Ginny Soybel. 2002. All About Pro Forma Accounting. *CPA Journal* 72(4): 13.

Harrison, Jennifer L., and Anja Morton. 2010. Adjusted earnings: an initial investigation of EPS disclosures in annual reports. *Euro-Mediterranean Economics and Finance Review* 5: 62-74.

He, Daoping. 2018. Empirical Evidence of the Rounding Phenomenon in Reported Pro Forma Earnings. *International Journal of Business* 23: 301-311.

Hebestreit, Gernot, and Evelyn Teitler-Feinberg. 2017. Alternative Performance Measures: Ist-Zustand und Stoßrichtung des IASB. *Zeitschrift für Internationale Rechnungslegung* 12: 169-176.

Heflin, Frank, and Charles Hsu. 2008. The impact of the SEC's regulation of non-GAAP disclosures. *Journal of Accounting and Economics* 46: 349-365.

Heflin, Frank, Charles Hsu, and Qinglu Jin. 2015. Accounting conservatism and Street earnings. *Review of Accounting Studies* 20: 674-709.

Heflin, Frank, Kalin S. Kolev, and Benjamin C. Whipple. 2021. *The Risk-Relevance of Non-GAAP Earnings*. Working Paper. University of Georgia, Athens and City University of New York, New York. November 2021. https://dx.doi.org/10.2139/ssrn.3222893. Accessed 17 January 2022.

Heidari, Hassan, Ali Ashtab, and Golamreza Kordestani. 2012. Predicting earnings management based on adjusted earnings per share (EPS). *African Journal of Business Management* 6: 8281-8286.

Heiden, Matthias. 2006. *Pro-forma-Berichterstattung – Reporting zwischen Information und Täuschung*. Berlin: Erich Schmidt Verlag.

Heitger, Dan L., and Brian Ballou. 2003. Pro forma earnings: Adding value or distorting perception? *CPA Journal* 73(3): 44-47.

Helikum, Lukas J. 2018. *The real effect of non-gaap disclosures and financial reporting consistency: an examination of managers' investment choices*. Dissertation Nanyang Business School, Singapore.

Henry, David, and Christopher H. Schmitt. 2001. The Numbers Game. *BusinessWeek* 73(3732): 100-110.

Henry, Elaine, Nan Hu, and Xi Jiang. 2020. Relative Emphasis on Non-GAAP Earnings in Conference Calls: Determinants and Market Reaction. *European Accounting Review* 29: 169-197.

Henry, Theresa F., David A. Rosenthal, and Rob R. Weitz. 2017. Recent Trends in Reporting Non-GAAP Income – An Example from Social Media Companies. *CPA Journal* 87(6): 60-64.

Henry, Theresa F., Rob R. Weitz, and David A. Rosenthal. 2020a. The Gap between GAAP and Non-GAAP. *CPA Journal* 90(2): 60-65.

Henry, Theresa F., Rob R. Weitz, and David A. Rosenthal. 2020b. Non-GAAP earnings disclosure post 2010 SEC regulation change. *Journal of Corporate Accounting & Finance* 31: 114-134.

Henry, Theresa, David Rosenthal, and Rob Weitz. 2019. Changes in Companies' Non-GAAP Reporting Behavior in Response to SEC Rule Revisions. Conference Paper. Northeast Decision Sciences Institute 2019 Annual Conference, Philadelphia.

Hillebrandt, Franca, and Thorsten Sellhorn. 2002. „Earnings before bad stuff" – Pro forma earnings disclosures in German annual reports. Discussion Paper 11/2002. Ruhr-University Bochum, Bochum. November 2002.

Hirsch, Manuela, and Christian Riegler. 2009. Pro forma-Ergebnisberichterstattung zwischen Investorinformation und Managerentlohnung. In *Jahrbuch für Controlling und Rechnungswesen 2009*, ed. Gerhard Seicht, 453-483. Wien: LexisNexis.

Hirshleifer, David, and Siew Hong Teoh. 2003. Limited attention, information disclosure, and financial reporting. *Journal of Accounting and Economics* 36: 337-386.

Hitz, Jörg-Markus, and Verena Jenniges. 2008. Publizität von Pro-forma-Ergebnisgrößen am deutschen Kapitalmarkt – Empirischer Befund für die IFRS-Rechnungslegung großer deutscher Kapitalgesellschaften. *Zeitschrift für internationale und kapitalmarktorientierte Rechnungslegung* 8: 236-245.

Hitz, Jörg-Markus. 2010a. Information versus adverse Anlegerbeeinflussung: Befund und Implikationen der empirischen Rechnungswesenforschung zur Publizität von Pro-forma-Ergebnisgrößen. *Journal für Betriebswirtschaft* 60: 127-161.

Hitz, Jörg-Markus. 2010b. Press Release Disclosure of 'Pro Forma' Earnings Metrics by Large German Corporations – Empirical Evidence and Regulatory Recommendations. *Accounting in Europe* 7: 63-86.

Hitzig, Neal B. 2016. Debating Non-GAAP Metrics. *CPA Journal* 86(9): 15.

Hogan, Brian R., Ganesh Krishnamoorthy, and James J. Maroney. 2017. Pro Forma Earnings Presentation Effects and Investment Decisions. *Behavioral Research in Accounting* 29: 11-24.

Höllerschmid, Christian, Daniela Maresch, and Alexander Schiebel. 2005. Earnings before Interest and Tax (EBIT) – eine empirische Analyse. *Zeitschrift für Recht und Rechnungswesen* 53: 179-183.

Holtzman, Mark, Robert Fonfeder, and J. K. Yun. 2003. Goodbye "Pro Forma" Earnings – The SEC tightens non-GAAP financial reporting. *Strategic Finance* 85(11): 1-3.

Hribar, Paul, Richard Mergenthaler, Aaron Roeschley, Spencer Young, and Chris X. Zhao. 2021. Do Managers Issue More Voluntary Disclosure When GAAP Limits Their Reporting Discretion in Financial Statements? *Journal of Accounting Research* 60: 299-351.

Hsieh, Hsin-Yi. 2010. *Balancing Investor's Information Needs with Accounting Conservatism: The Role of Voluntary Disclosure of Non-GAAP Earnings*. Dissertation University of Oklahoma, Norman.

Hsu, Charles, and William Kross. 2011. The Market Pricing of Special Items that are Included in versus Excluded from Street Earnings. *Contemporary Accounting Research* 28: 990-1017.

Hsu, Charles, Rencheng Wang, and Benjamin C. Whipple. 2021. Non-GAAP earnings and stock price crash risk. *Journal of Accounting and Economics*. https://doi.org/10.1016/j.jacceco.2021.101473.

Hsu, Charles. 2004. *Strategic Choices of Street Earnings and Earnings Perceptions Management*. Working Paper. Purdue University, West Lafayette. January 2004.

Huang, Qianyun, and Terrance R. Skantz. 2016. The informativeness of pro forma and street earnings: an examination of information asymmetry around earnings announcements. *Review of Accounting Studies* 21: 198-250.

Hummel, Hans-Peter. 2007. Der einfache Weg. *Personal* 59(3): 26-28.

Hummel, Katrin, and Stefan Beeler. 2013. Pro-forma-Berichterstattung am Schweizer Kapitalmarkt. In *Finanz- und Rechnungswesen: Jahrbuch 2013*, ed. Conrad Meyer and Dieter Pfaff, 157-186. Zurich: WEKA Verlag.

Ibrani, Ewing Yuvisa, Faisal Faisal, and Yenny Dwi Handayani. 2019. Determinant of non-GAAP earnings management practices and its impact on firm value. *Cogent Business & Management*. https://doi.org/10.1080/23311975.2019.1666642.

Immenkötter, Philipp. 2020. Pro-forma: Bereinigt, aber nicht sauber. *Zeitschrift für internationale und kapitalmarktorientierte Rechnungslegung* 20: 165-170.

Isidro, Helena, and Ana Marques. 2013. Response to Discussion of: The Effects of Compensation and Board Quality on Non-GAAP Disclosures in Europe. *The International Journal of Accounting* 48: 324-326.

Isidro, Helena, and Ana Marques. 2013. The Effects of Compensation and Board Quality on Non-GAAP Disclosures in Europe. *The International Journal of Accounting* 48: 289-317.

Isidro, Helena, and Ana Marques. 2015. The Role of Institutional and Economic Factors in the Strategic Use of Non-GAAP Disclosures to Beat Earnings Benchmarks. *European Accounting Review* 24: 95-128.

Isidro, Helena, and Ana Marques. 2021. Industry competition and non-GAAP disclosures. *Accounting and Business Research* 25: 156-184.

Islam, Nahid, John Evans, Greg White, and Md Mosharraf Hossain. 2019. Components of CEO Remuneration and Non-GAAP Disclosure. *Australian Accounting Review* 29: 615-630.

James, Kevin L., and Franklin A. Michello. 2003. The dangers of pro forma reporting. *CPA Journal* 73(2): 65-67.

Jana, Stephanie, and Kevin McMeeking. 2021. Alternative Performance Measures: Determinants of Disclosure Quality – Evidence from Germany. *Accounting in Europe* 18: 102-142.

Jarolim, Natascha, and Carina Öppinger. 2014. Kennzahlenpublizität europäischer börsennotierter Unternehmen – Eine empirische Analyse. *Zeitschrift für Internationale Rechnungslegung* 9: 205-211.

Jarva, Henry, Juha-Pekka Kallunki, and Gilad Livne. 2019. Earnings performance measures and CEO turnover: Street versus GAAP earnings. *Journal of Corporate Finance* 56: 249-266.

Jennings, Marianne M. 2003. Ethics and non-GAAP financial reporting. *Corporate Finance Review* 8(3): 43-46.

Jennings, Ross, and Ana Marques. 2007. *The impact of corporate governance on the disclosure of manager-adjusted non-GAAP earnings*. Working Paper. University of Texas, Austin and Universidade Nova de Lisboa, Lisbon. September 2007. https://dx.doi.org/10.2139/ssrn.1014123. Accessed 17 January 2022.

Jennings, Ross, and Ana Marques. 2011. The Joint Effects of Corporate Governance and Regulation on the Disclosure of Manager-Adjusted Non-GAAP Earnings in the US. *Journal of Business Finance & Accounting* 38: 364-394.

Jennings, Ross, Marc LeClere, and Robert B. Thompson. 2001. Goodwill Amortization and the Usefulness of Earnings. *Financial Analysts Journal* 57: 20-28.

Jo, Koren M., and Shuo Yang. 2020. SEC Comment Letters on Firms' Use of Non-GAAP Measures: The Determinants and Firms' Responses. *Accounting Horizons* 34: 167-184.

Johnson, Amber, Majella Percy, Peta Stevenson-Clarke, and Robyn Cameron. 2014. The Impact of the Disclosure of Non-GAAP Earnings in Australian Annual Reports on Non-Sophisticated Users. *Australian Accounting Review* 24: 207-217.

Johnson, W. Bruce, and William C. Schwartz. 2005. Are Investors Misled by "Pro Forma" Earnings? *Contemporary Accounting Research* 22: 915-963.

Kabureck, Gary R. 2017. *Accounting for non-GAAP earnings measures*. https://www.ifrs.org/news-and-events/2017/03/accounting-for-non-gaap-earnings-measures. Accessed 17 January 2022.

Kaplan, Zachary, Xiumin Martin, and Yifang Xie. 2017. *How does data vendor discretion affect street earnings?* Working Paper. Washington University, St. Louis. November 2017.

Karamanou, Irene. 2007. Discussion of Implications of Components of Income Excluded from Pro Forma Earnings for Future Profitability and Equity Valuation. *Journal of Business Finance & Accounting* 34: 676-679.

Kent, Pamela, and Lois Munro. 1999. Differential Reporting and the Effect on Loan Evaluations: An Experimental Study. *Accounting Forum* 23: 359-377.

Kim, Hyo Jin, and Soon Suk Yoon. 2019. Value-relevance of the regulatory non-GAAP adjustments in the Korean banking industry. *Asia-Pacific Journal of Accounting & Economics* 26: 160-171.

Kiosse, Paraskevi Vicky. 2009. Discussion of US Managers' Use of 'Pro Forma' Adjustments to Meet Strategic Earnings Targets. *Journal of Business Finance & Accounting* 36: 327-335.

Kleinmanns, Hermann. 2016. ESMA veröffentlicht Leitlinien zu alternativen Leistungskennzahlen – ein Schritt in die richtige Richtung? *Zeitschrift für Internationale Rechnungslegung* 11: 131-136.

Knapova, Bohuslava, Tomas Krabec, and Jaroslava Roubickova. 2011. EBIT Criterion: Financial Analysis' Issues. *International Journal of Mathematical Models and Methods in Applied Sciences* 5: 499-507.

Kolev, Kalin, Carol A. Marquardt, and Sarah E. McVay. 2008. SEC Scrutiny and the Evolution of Non-GAAP Reporting. *Accounting Review* 83: 157-184.

Koning, Miriam, Gerard Mertens, and Peter Roosenboom. 2010. The Impact of Media Attention on the Use of Alternative Earnings Measures. *Abacus* 46: 258-288.

Kriete, Thomas, Thomas Padberg, and Thomas Werner. 2003. Zur Verbreitung und Objektivierung von „Earnings-before„-Kennzahlen in Europa. *BBK Betrieb und Rechnungswesen* 50: 507-514.

Kühnberger, Manfred, and Philipp Thurmann. 2013. Pro-forma Earnings bei Immobilien-AG. *Zeitschrift für internationale und kapitalmarktorientierte Rechnungslegung* 13: 281-292.

Kuo, Chii-Shyan, Jow-Ran Chang, and Shih-Ti Yu. 2013. Effect of mandatory pro forma earnings disclosure on the relation between CEO share bonuses and firm performance. *Review of Quantitative Finance and Accounting* 40: 189-215.

Küting, Karlheinz, and Matthias Heiden. 2003. Zur Systematisierung von Pro-forma-Kennzahlen – Gleichzeitig: Fortsetzung einer empirischen Bestandsaufnahme. *Deutsches Steuerrecht* 41: 1544-1552.

Kyung, Hangsoo, Hakyin Lee, and Carol Marquardt. 2019. The effect of voluntary clawback adoption on non-GAAP reporting. *Journal of Accounting and Economics* 67: 175-201.

Kyung, Hangsoo, Jeff Ng, and Yong George Yang. 2021. Does the use of non-GAAP earnings in compensation contracts lead to excessive CEO compensation? Efficient contracting versus managerial power. *Journal of Business Finance & Accounting* 48: 841-868.

Lambert, Richard A. 2004. Discussion of analysts' treatment of non-recurring items in street earnings and loss function assumptions in rational expectations tests on financial analysts' earnings forecasts. *Journal of Accounting and Economics* 38: 205-222.

Landsman, Wayne R., Bruce L. Miller, and Shu Yeh. 2007. Implications of Components of Income Excluded from Pro Forma Earnings for Future Profitability and Equity Valuation. *Journal of Business Finance & Accounting* 34: 650-675.

Lau, Chungfoon. 2001. Pro Forma Stance Backed. *Financial Executive* 17(6): 9.

Laurion, Henry, and Richard Sloan. 2021. When does forecasting GAAP earnings entail unreasonable effort? *Journal of Accounting and Economics*. https://doi.org/10.1016/j.jacceco.2021.101437.

Laurion, Henry. 2018. *Do Non-GAAP Earnings Influence Real Activities and Accounting Choices?* Dissertation University of California, Berkeley.

Laurion, Henry. 2020. Implications of Non-GAAP Earnings for Real Activities and Accounting Choices. *Journal of Accounting & Economics*. https://doi.org/10.1016/j.jacceco.2020.101333.

Le, Cao Hoang Anh, Yaowen Shan, and Stephen Taylor. 2020. Executive Compensation and Financial Performance Measures: Evidence from Significant Financial Institutions. *Australian Accounting Review* 30: 159-177.

Lee, Alan, and A. Scott Davidson. 2005. Misunderstood and misused. *CA Magazine* 138(1): 39-40.

Lee, Cheng-Hsun. 2021. Non-generally accepted accounting principles disclosures and audit committee chairs' external directorships. *Journal of Business Finance & Accounting*. https://doi.org/10.1111/jbfa.12566.

Lee, Robert, and Anthony P. Curatola. 2017. Does the Content of Pro Forma Earnings Influence Nonprofessional Investors? *Management Accounting Quarterly* 18(4): 1-7.

Lee, Yen-Jung, and Cheng-Ping Chu. 2016. Causes and Consequences of Firms' Decision to Discontinue Non-GAAP Earnings Disclosure in Earnings Releases. *Journal of Accounting Review* 63: 1-46.

Leibfried, Peter, and Andres Venzin. 2014. Earnings Before Bad Stuff: Pro-forma-Kennzahlen am Schweizer Aktienmarkt. *Zeitschrift für Internationale Rechnungslegung* 9: 61-65.

Leibfried, Peter, Karla Linden, Silvan Jurt, and Olivia Bischoff. 2018. *Bridge the Gaps – How to improve reporting of Alternative Performance Measures*. https://www.alexandria.unisg.ch/254352/1/bridge_the_gaps_broschure_final.pdf. Accessed 17 January 2022.

Leung, Edith, and David Veenman. 2018. Non-GAAP Earnings Disclosure in Loss Firms. *Journal of Accounting Research* 56: 1083-1137.

Lev, Baruch. 2011. How to Win Investors Over. *Harvard Business Review* 89(11): 52-62.

Levinsohn, Alan. 2002. Popularity of "Pro Forma" Earnings Prompts Reform. *Strategic Finance* 83(8): 63.

Lewis, Michael. 2002. Pro forma LINGO – Does the use of controversial non-GAAP reporting by some companies confuse or enlighten? *CA Magazine* 135(2): 16-24.

Lienau, Achim, and Lars Schiemann. 2021. Regulierung der Pro-forma-Berichterstattung und Entwicklung der Qualität der Überleitungsrechnungen. *Zeitschrift für internationale und kapitalmarktorientierte Rechnungslegung* 21: 23-30.

Lin, Shu, Hui Harry Xia, and Tatyana Ryabova. 2020. The effect of analysts' GAAP earnings forecasts on managers' classification shifting. *Journal of Contemporary Accounting & Economics*. https://doi.org/10.1016/j.jcae.2020.100222.

Linsmeier, Thomas J. 2016. Revised Model for Presentation in Statement(s) of Financial Performance: Potential Implications for Measurement in the Conceptual Framework. *Accounting Horizons* 30: 485-498.

Liu, Bo, and Dana Zhang. 2020. The use of non-GAAP measures in initial public offerings. *The Journal of Corporate Accounting & Finance* 31: 60-72.

Livingston, Phil. 2001. Let's Be Balanced with Pro Forma Earnings. *Financial Executive* 17(4): 6.

Lont, David H., Dinithi Ranasinghe, and Helen Roberts. 2020. Non-GAAP Disclosures and CEO Pay Levels. *The International Journal of Accounting*. https://doi.org/10.1142/S109440602050016X.

Lopez, Thomas J., Chris McCoy, Gary Taylor, and Michael Young. 2019. *Are Investors Misled by non-GAAP Expense Exclusions Used to Beat Analysts' Earnings Forecasts?* Working Paper. University of Alabama, Tuscaloosa et al. September 2019. https://dx.doi.org/10.2139/ssrn.3443860. Accessed 17 January 2020.

Lougee, Barbara A., and Carol A. Marquardt. 2004. Earnings Informativeness and Strategic Disclosure: An Empirical Examination of "Pro Forma" Earnings. *Accounting Review* 79: 769-795.

Luciano, Robert. 2003. EBITDA as an indicator of earnings quality. *JASSA* 10(1): 29-34.

MacDonald, Elizabeth. 2002. Pro Forma Puff Jobs. *Forbes*. 170(12): 172-173.

MacDonald, Elizabeth. 2003. The Ebitda Folly. *Forbes* 171(3): 165-167.

Magli, Francesca, Alberto Nobolo, and Matteo Ogliari. 2017. Alternative Performance Measures and ESMA Guidelines: Improving Stakeholders' Communication. *International Journal of Business and Management* 12: 15-28.

Malone, Lance, Ann Tarca, and Marvin Wee. 2016. IFRS non-GAAP earnings disclosures and fair value measurement. *Accounting & Finance* 56: 59-97.

Marcogliese, Pam, and Dase Kim. 2016. Non-GAAP: The Pendulum Swings Back. *The Corporate Governance Advisor* 24(4): 1-6.

Maresch, Daniela, and Alexander Schiebel. 2005. Earnings before Interest and Tax (EBIT) im IFRS-Konzernabschluss. *Zeitschrift für Recht und Rechnungswesen* 15: 140-143.

Maresch, Daniela, and Alexander Schiebel. 2005. Earnings before Interest and Tax (EBIT) im handelsrechtlichen Einzelabschluss. *Zeitschrift für Recht und Rechnungswesen* 15: 117-120.

Marques, Ana. 2006. SEC interventions and the frequency and usefulness of non-GAAP financial measures. *Review of Accounting Studies* 11: 549-574.

Marques, Ana. 2007. *What do the S&P 500 disclose in their earnings announcements? – Evidence on financial statements and non-GAAP financial measures*. Working Paper. Universidade Nova de Lisboa, Lisbon. November 2007. https://dx.doi.org/10.2139/ssrn.1105446. Accessed 17 January 2022.

Marques, Ana. 2010. Disclosure strategies among S&P 500 firms: Evidence on the disclosure of non-GAAP financial measures and financial statements in earnings press releases. *The British Accounting Review* 42: 119-131.

Marques, Ana. 2017. Non-GAAP earnings: international overview and suggestions for future research. *Meditari Accountancy Research* 25: 318-335.

Masihabadi, Abolghasem, Ali Taghavi Moghaddam, Amir Shams Kulukhi, and Rouhollah Rahmani. 2015. The relationship between earnings before interest and taxes and operating cash flow and stock return under the condition of information asymmetry in Abadan and Arak Petrochemical Companies through markovswitching approach. *Marketing and Branding Research* 2: 74-88.

McConnell, Patricia. 2014. One fewernon-GAAP adjustment to worry about: improvements to the accounting for changes in own credit. *Investor Perspectives*. https://cdn.ifrs.org/-/media/feature/resources-for/investors/investor-perspectives/investor-perspective-mar-2014.pdf. Accessed 17 January 2022.

Mehring, Oliver, Jens Müller, Sönke Sievers, and Christian Sofilkanitsch. 2021. *Does learning about low GAAP reporting quality change investors' perceptions of aggressive non-GAAP reporting choices?* TRR 266 Accounting for Transparency Working Paper. University of Paderborn, Paderborn. March 2021. https://dx.doi.org/10.2139/ssrn.3415109. Accessed 17 January 2022.

Mey, Mattheus Theodorus, and Christiaan Lamprecht. 2021. The association between EBITDA reconciliation quality and opportunistic disclosure. *South African Journal of Accounting Research* 35: 87-110.

Miller, Gunnar. 2014. Discussion of 'The drivers, consequences and policy implications of non-GAAP earnings reporting' by Steven Young (2014). *Accounting and Business Research* 44: 466-468.

Miller, Jeffrey S. 2009. Opportunistic Disclosures of Earnings Forecasts and Non-GAAP Earnings Measures. *Journal of Business Ethics* 89: 3-10.

Moehrle, Stephen R., Jennifer A. Reynolds-Moehrle, and James S. Wallace. 2003. Dining at the earnings buffet. *Business Horizons* 46(4): 61-67.

Moehrle, Stephen R., Jennifer A. Reynolds-Moehrle, and James S. Wallace. 2001. How Informative Are Earnings Numbers That Exclude Goodwill Amortization? *Accounting Horizons* 15: 243-255.

Morgan, Mark, V. Brooks Poole, Huan Qiu, and Christa A. Owen. 2018. Introduce Non-GAAP Metrics to Business Students. *Business Education Innovation Journal* 10: 116-127.

Morton, John. 2002. Accounting 201 – What is pro forma reporting, and why is it used? *American Journalism Review* 24(8): 88.

Moscariello, Nicola. 2020. The Economics of Non-GAAP Measures and Regulation. In *Reporting Non-GAAP Financial Measures*, ed. Nicola Moscariello and Michele Pizzo, 18-37. Newcastle: Cambridge Scholars Publishing.

Mullaney, Timothy J. 2002. Amazon is all grown up, except for its accounting. *BusinessWeek* 74(3794): 74.

N.N. 2001. Differing Results. *Financial Executive* 17(7): 12.

N.N. 2002. Pro Forma Warning. *Financial Executive* 18(1): 60-62.

N.N. 2002. Pro-forma accounting – Out, by $100 billion – Nasdaq firms' pro-forma alchemy. *The Economist* 362(8261): 77.

Nagel, Gabriela, and Marco Passardi. 2009. Performance Reporting bei Schweizer Banken – Pro Forma-Berichterstattung und Ergebnisausweis nach Regelwerk. *Zeitschrift für Internationale Rechnungslegung* 4: 439-447.

Nam, Seunghan, and J. K. Yun. 2021. *Do firms Use Non-GAAP Earnings to Mask their Poor Performance and Get Away with it?* Working Paper. New York Institute of Technology, New York. July 2021. https://dx.doi.org/10.2139/ssrn.3776532. Accessed 17 January 2022.

Narayanaswamy, Ramnath. 2021. *The Use and Abuse of Non-GAAP Financial Measures: An Exploratory Study of Indian Companies*. IIM Bangalore Research Paper No. 637. Indian Institute of Management Bangalore, Bangalore. March 2021. https://dx.doi.org/10.2139/ssrn.3807818. Accessed 17 January 2022.

Nichols, Nancy B., Sidney J. Gray, and Donna L. Street. 2005. Pro Forma Adjustments to GAAP Earnings: Bias, Materiality, and SEC Action. *Research in Accounting Regulation* 18: 29-52.

Nie, Dongfang, and Chunhao Xu. 2021. Non-GAAP earnings quality in firms with data breach incident. *Asian Review of Accounting* 29: 383-398.

Nissim, Doron. 2019. *EBITDA, EBITA or EBIT?* Working Paper. Columbia Business School, New York City. October 2019. https://dx.doi.org/10.2139/ssrn.2999675. Accessed 17 January 2022.

Nyberg, Alix. 2004. A Matter of Emphasis – Regulation G was supposed to end the abuses of pro forma reporting. Has it succeeded? *CFO Magazine* 20(9): 69-70.

Oesch, David, and Tanja Walser. 2019. Non-GAAP-Reporting kotierter Schweizer Firmen. In *Finanz- und Rechnungswesen: Jahrbuch 2019*, ed. Reto Eberle, David Oesch and Dieter Pfaff, 145-171. Zurich: WEKA Business Media.

Palmrose, Zoe-Vonna, and Susan Scholz. 2004. The Circumstances and Legal Consequences of Non-GAAP Reporting: Evidence from Restatements. *Contemporary Accounting Research* 21: 139-180.

Papa, Vincent T., Sandra J. Peters, and Kurt Schacht. 2016. Investor Uses, Expectations and Concerns on Non-GAAP Financial Measures. https://www.cfainstitute.org/-/media/documents/support/advocacy/investor-uses-expectations-concerns-on-non-gaap.ashx. Accessed 17 January 2022.

Papa, Vincent. 2017. Non-GAAP Reporting Trends and Investor Expectations. *Zeitschrift für Internationale Rechnungslegung* 12: 221-227.

Parrino, Richard J. 2016. New compliance guidance by SEC staff signals increased scrutiny of non-GAAP financial measures. *Journal of Investment Compliance* 17: 23-33.

Parrino, Richard J. 2019. Bringing order to non-GAAP financial measures: SEC sues to enforce "equal-or-greater-prominence" requirement. *Journal of Investment Compliance* 20: 51-57.

Parsian, Hossein, Amir Shams Koloukhi, and Mojtaba Akbarpour. 2013. The Relationship between Earnings Before Interest and Tax and Operating Cash Flow and Stock Return in Information Asymmetry Conditions at Pharmaceutical Companies of Abidi and Darou Pakhsh Applying Markov-Switching Approach. *Interdisciplinary Journal of Contemporary Research in Business* 5: 173-185.

Phillips, Thomas J., Michael S. Luehlfing, and Cynthia Waller Vallario. 2002. Hazy Reporting. *Journal of Accountancy* 194: 47-53.

Pizzo, Michele. 2020. Definitions and Trends in Non-GAAP Measures and Disclosure. In *Reporting Non-GAAP Financial Measures*, ed. Nicola Moscariello and Michele Pizzo, 2-17. Newcastle: Cambridge Scholars Publishing.

Pounder, Bruce. 2011. Non-GAAP Financial Measures: What Not to Report. *Strategic Finance* 93(10): 20-22.

Rainsbury, Elizabeth A. 2017. The Impact of the FMA Guidelines on Non-GAAP Earnings Disclosures. *Australian Accounting Review* 27: 480-493.

Rainsbury, Liz, Carol Hart, and Nonthipoth Buranavityawut. 2015. GAAP-adjusted earnings disclosures by New Zealand companies. *Pacific Accounting Review* 27: 329-352.

Rashty, Josef, and John O'Shaughnessy. 2014. Reporting and Disclosures Using Non-GAAP Financial Measures. *CPA Journal* 84(3): 36-39.

Reimsbach, Daniel, and Raimund Schirmeister. 2012. „Earnings-before"-Kennzahlen in IFRS-Abschlüssen – Regulierungsdebatte vor dem Hintergrund des Joint Financial Statement Presentation Project. *Die Wirtschaftsprüfung* 65: 129-136.

Reimsbach, Daniel. 2014. Pro forma earnings disclosure: the effects of non-GAAP earnings and earnings-before on investors' information processing. *Journal of Business Economics* 84: 479-515.

Ribeiro, Andrea, Yaowen Shan, and Stephen Taylor. 2019. Non-GAAP Earnings and the Earnings Quality Trade-off. *Abacus* 55: 6-41.

Rieg, Robert. 2010. Proforma-Kennzahlen – kein Ende der EBITANEI in Sicht? *Zeitschrift für Bilanzierung, Rechnungswesen und Controlling* 34: 252-254.

Rozenbaum, Oded. 2019. EBITDA and Managers' Investment and Leverage Choices. *Contemporary Accounting Research* 36: 513-546.

Ruhwedel, Franca, and Sarah Thale. 2013. Pro-Forma-Ergebnisse – Augenwischerei oder Transparenzgewinn? *Controlling* 25: 386-393.

Ruhwedel, Franca, Pascal Hemmersbach, and Philipp Mosch. 2017. Pro Forma-Ergebnisse im Value Reporting von DAX und MDAX – Änderungsbedarfe durch die neuen ESMA-Leitlinien zu Alternativen Leistungskennzahlen? *Controlling* 29: 19-26.

Sadique, M. Shibley, and M. Arifur Rahman. 2013. Investor reaction to strategic emphasis on earnings numbers: An empirical study. *Contemporary Economics* 7: 51-64.

Sarpong, Emmanuel. 2021. *The Relationship Between Earnings Before Interest and Taxes (EBIT) and Financial Distress in the Renewable Energy Industry*. Dissertation Northcentral University, La Jolla.

Schäfer, Gabriele. 2010. Online-Fachinfos zur Erfolgskennzahl EBIT. *Zeitschrift für Bilanzierung, Rechnungswesen und Controlling* 34: 111.

Schiff, Allen I., and Jonathan B. Schiff. 2003. Getting to the core. *CPA Journal* 73(6): 18.

Schiff, Allen I., and Jonathan B. Schiff. 2003. In Search of a Pro Forma Earnings Standard. *Management Accounting Quarterly* 5(1): 40-44.

Schirmeister, Raimund, and Daniel Reimsbach. 2011. EBIT-Kennzahlen in der Unternehmenspublizität. In *Jahrbuch für Controlling und Rechnungswesen 2011*, ed. Gerhard Seicht, 245-264. Wien: LexisNexis.

Schuler, Thomas. 2011. Kritische Auseinandersetzung mit den Steuerungsgrößen EBIT und Cash Flow und deren Bedeutung in wirtschaftlich unsicheren Zeiten. In *Publikationsreihe Ganzheitliches Management in der Praxis*, ed. Christian Abegglen, 1-77. St. Gallen: Business Books & Tools St. Gallen.

Schwager, Jack D. 2013. Sense and Nonsense About Pro Forma Statistics. In *Market Sense and Nonsense: How the Markets Really Work (and How They Don't)*, ed. Jack D. Schwager, 133-136. Hoboken: Wiley.

Seetharaman, Ananth, Xu Wang, and Sanjian Zhang. 2014. An Empirical Analysis of the Effects of Accounting Expertise in Audit Committees on Non-GAAP Earnings Exclusions. *Accounting Horizons* 28: 17-37.

Sek, Julie, and Stephen Taylor. 2011. Profit or Prophet? – A Case Study of the Reporting of Non-GAAP Earnings by Australian Banks. *Australian Accounting Review* 21: 327-339.

Sellhorn, Thorsten, Katharina Hombach, and Christian Stier. 2014. *Strategische Finanzberichterstattung durch Pro forma-Kennzahlen und Finanzgrafiken – Herausforderung für die Abschlussanalyse*. Düsseldorf: Hans-Böckler-Stiftung.

Selling, Thomas I., and Gregory A. Sommers. 2016. Why Accountants Should Care about Non-GAAP Financial Metrics. *CPA Journal* 86(6): 12-13.

Sherman, H. David, and S. David Young. 2018. The Pitfalls of Non-GAAP Metrics. *MIT Sloan Management Review* 59(2): 57-63.

Shiah-Hou, Shin-Rong, and Yi-Yun Teng. 2016. The informativeness of non-GAAP earnings after Regulation G? *Finance Research Letters* 18: 184-192.

Shiah-Hou, Shin-Rong. 2021. The relation between non-GAAP earnings and accounting restatements: Evidence after regulation G. *Advances in Accounting*. https://doi.org/10.1016/j.adiac.2021.100567.

Shibasaki, Yuta, and Chikara Toyokura. 2020. The Disclosure of Non-GAAP Performance Measures and the Adoption of IFRS: Evidence from Japanese Firms' Experience. *Monetary and Economic Studies* 38: 19-54.

Singleton-Green, Brian. 2001. Editorial: The deadly ebitda virus – The importance of the bottom line. *Accountancy Daily* 127(1291): 24.

Sinnewe, Elisabeth, Jennifer L. Harrison, and Albert Wijeweera. 2017. Future Cash Flow Predictability of Non-IFRS Earnings: Australian Evidence. *Australian Accounting Review* 27: 118-128.

Sloan, Richard. 1998. Discussion of "Evaluating Non-GAAP Performance Measures in the REIT Industry". *Review of Accounting Studies* 3: 131-135.

Smetanka, Rick. 2012. GAAP or Non-GAAP? *Financial Executive* 28(9): 13-14.

Sofilkanitsch, Christian. 2021. *Does Non-GAAP Reporting Change after Financial Restatements?* Working Paper. Paderborn University, Paderborn. November 2011. https://dx.doi.org/10.2139/ssrn.3987173. Accessed 17 January 2022.

Solsma, Lori, and W. Mark Wilder. 2015. Pro forma disclosure practices of firms applying IFRS. *International Journal of Accounting and Information Management* 23: 383-403.

Stahlin, Paul V., and Neville Grusd. 2011. Blue Ribbon Panel on Private Company Financial Reporting. *CPA Journal* 81(7): 6-10.

Stenheim, Tonny, Anna Natalia Beckman, Cathrine Olsen Valltoft, and Dag Øivind Madsen. 2018. The value relevance of alternative performance measures: Evidence from the Oslo Stock Exchange. *Journal of Governance and Regulation* 7: 27-41.

Strischek, Dev. 2001. E-B-I-T-D-A – It Doesn't Spell "Cash Flow". *The RMA Journal* 84(3): 30-41.

Stuart, Iris C., and Vijay Karan. 2003. eToys Inc.: A Case Examining Pro Forma Financial Reports, Analysts' Forecasts, and Going Concern Disclosures. *Issues in Accounting Education* 18: 191-209.

Tanski, Joachim S. 2021. EBITDA – Irreführung oder Irrtum? *Der Betrieb* 74: 2777-2781.

Taylor, Stacey, and Vlado Keselj. 2020. *Using Extractive Lexicon-based Sentiment Analysis to Enhance Understanding of the Impact of Non-GAAP Measures in Financial Reporting*. Conference Paper. Second Workshop on Financial Technology and Natural Language Processing in conjunction with IJCAI-PRICAI 2020, Kyoto. 5 January 2021.

Teh, Seng Thiam. 2014. *The Impact of the Global Financial Crisis on the Comparative Value Relevance of GAAP versus Non-GAAP Earnings*. Dissertation Australian National University, Canberra.

Teucher, Christoph, and Nicole V. S. Ratzinger-Sakel. 2021. Alternative Leistungskennzahlen und Bereinigungen in der Prognoseberichterstattung – Eine Bestandsaufnahme vor dem Hintergrund der ESMA-Leitlinien. *Die Wirtschaftsprüfung* 71: 297-304.

Thielemann, Felix, and Tami Dinh. 2019. Non-GAAP earnings disclosures around regulation G – The case of "implicit non-GAAP reporting". *Advances in Accounting*. https://doi.org/10.1016/j.adiac.2019.100432.

Thielemann, Felix, Tami Dinh, and Helen Kang. 2019. Non-GAAP Reporting and Debt Market Outcomes: Evidence from Regulation G. *Schmalenbach Business Review* 71: 169-203.

Torghabeh, Mohammad Reza Karimi, Hossein Parsian, and Amir Shams Kolookhi. 2014. A study on relationship between earnings before tax, interest and operational cash flows with stockholders' equity. *Management Science Letters* 4: 1699-1706.

Trotter, Joel, Steven Stokdyk, and Nathan Ajiashvili. 2013. Giving Good Guidance. *Financial Executive* 29(3): 57-60.

Turner, Eric. 2004. New reporting framework. *CA Magazine* 137(1): 47-49.

Tutino, Marco. 2011. Which metrics are relevant in European listed companies? – Evidence from Nineties. *Corporate Ownership and Control* 8: 566-588.

Upmeier, Juliane-Rebecca, and Inge Wulf. 2021. Auswirkungen von IFRS 16 auf alternative Leistungskennzahlen – Regulatorik und Analyse am Beispiel der DAX-, MDAX- und SDAX-Konzerne für das Geschäftsjahr 2019. *Die Wirtschaftsprüfung* 74: 636-643.

Vasconcelos de Andrade, Gabriela, and Fernando Dal-Ri Murcia. 2019. A critical analysis on the additional adjustments considered in the disclosure of the non-GAAP "adjusted EBITDA" measure in the reports of Brazilian listed companies. *Journal of Education and Research in Accounting* 13: 477-494.

Vater, Hendrik J., and Christoph R. Kley. 2003. Pro-forma-Gewinne: Informations-Tohuwabohu? – Zur Konzeption von Proforma-Gewinnen: Ein Blick in Praxis und Theorie. *BankArchiv* 51: 491-499.

Venter, Elmar R., David Emanuel, and Steven F. Cahan. 2014. The Value Relevance of Mandatory Non-GAAP Earnings. *Abacus* 50: 1-24.

Venter, Elmar R., Steven F. Cahan, and David Emanuel. 2013. Mandatory Earnings Disaggregation and the Persistence and Pricing of Earnings Components. *The International Journal of Accounting* 48: 26-53.

Vincent, Linda. 1999. The information content of funds from operations (FFO) for real estate investment trusts (REITs). *Journal of Accounting and Economics* 26: 69-104.

Vinciguerra, R., F. Cappellieri, and A. Gravante. 2020. The Effects of the ESMA Guidelines on the Behaviour of Companies on Non-GAAP Disclosure in Europe. In *Reporting Non-GAAP Financial Measures*, ed. Nicola Moscariello and Michele Pizzo, 287-334. Newcastle: Cambridge Scholars Publishing.

Visani, Franco, F. Marta L. Di Lascio, and Silvia Gardini. 2020. The impact of institutional and cultural factors on the use of non-GAAP financial measures. International evidence from the oil and gas industry. *Journal of International Accounting, Auditing and Taxation*. https://doi.org/10.1016/j.intaccaudtax.2020.100334.

Viswam, Sonia. 2018. The Use of EBITDA in Representing the Performance of a Firm: An Analysis in the Context of IFRS. *International Journal of Research in Applied Management, Science & Technology*. https://www.zenonpub.com/images/pdf-files/Vol3/Issue3/5SoniaViswam.pdf. Accessed 17 January 2022.

Volk, Gerrit. 2007. Pro-forma-Kennzahlen in der Ergebnisberichterstattung 2005 der DAX30-Unternehmen. *Zeitschrift für Internationale Rechnungslegung* 2: 251-257.

Volk, Gerrit. 2007. Pro-forma-Kennzahlen in der Ergebnisberichterstattung 2006. *Die Aktiengesellschaft* 52: R378-R379.

Volk, Gerrit. 2007. Pro-forma-Kennzahlen zwischen Information und gezielter Fehlinformation. In *Rechnungslegung, Eigenkapital und Besteuerung*, ed. Norbert Winkeljohann, Peter Bareis, Michael Hinz and Gerrit Volk, 127-144. Munich: Vahlen Verlag.

Wahlen, James M. 2004. Discussion of "The Circumstances and Legal Consequences of Non-GAAP Reporting: Evidence from Restatements". *Contemporary Accounting Research* 21: 181-190.

Walker, Donald A. 2016. AICPA SEC and PCAOB National Conference Highlights. *Journal of Corporate Accounting & Finance* 27: 91-93.

Walker, Donald A. 2016. The Use of Non-GAAP Disclosures. *The Journal of Corporate Accounting & Finance* 27: 91-93.

Wallace, Wanda A. 2002. *Pro Forma Before and After the SEC's Warning: A Quantification of Reporting Variances from GAAP*. Morristown: Financial Executives Research Foundation.

Wallace, Wanda A. 2003. Analyzing Non-GAAP Line Items in Income Statements. *CPA Journal* 73(6): 38-47.

Webber, Sarah J., Nancy B. Nichols, Donna L. Street, and Sandra J. Cereola. 2013. Non-GAAP adjustments to net income appearing in the earnings releases of the S&P 100: An analysis of frequency of occurrence, materiality and rationale. *Research in Accounting Regulation* 25: 236-251.

Weinstein, Edward A., and Stanley Goldstein. 2016. Sustainability – Not GAAP Measurement. *CPA Journal* 86(6): 6-8.

Weirich, Thomas R., and Robert W. Rouse. 2002. The Moral (and Real-World) Hazard of Pro Forma Financials. *The Journal of Corporate Accounting & Finance* 13(5): 45-49.

Whipple, Benjamin C. 2015. *The great unknown: why exclude "other" items from non-GAAP earnings calculations in the post-Reg. G world?* Working Paper. University of Georgia, Athens. September 2015. https://dx.doi.org/10.2139/ssrn.2480663. Accessed 17 January 2022.

Whitehouse, Tammy. 2018. Companies advised to stay alert to non-GAAP issues. *Compliance Week* 15(169): 34-37.

Wieland, Matthew M., Mark C. Dawkins, and Michael T. Dugan. 2013. The Differential Value Relevance of S&P's Core Earnings Versus GAAP Earnings: The Role of Stock Option Expense. *Journal of Business Finance & Accounting* 40: 55-81.

Wong, Jilnaught, and Norman Wong. 2010. Voluntary disclosure of operating income. *Accounting & Finance* 50: 221-239.

Wu, Shih-Wei, Fengyi Lin, and Wenchang Fang. 2012. Earnings Management and Investor's Stock Return. *Emerging Markets Finance and Trade* 48: 129-140.

Yang, Yiru. 2018. Do aggressive pro forma earnings-reporting firms have difficulty disclosing intellectual capital? Australian evidence. *Journal of Intellectual Capital* 19: 875-896.

Yi, Han. 2012. Has Regulation G Improved the Information Quality of Non-GAAP Earnings Disclosures? *Seoul Journal of Business* 18: 95-145.

Young, Steven. 2014. The drivers, consequences and policy implications of non-GAAP earnings reporting. *Accounting and Business Research* 44: 444-465.

Yu, Junli. 2007. *The Research on Measuring Determining and Evaluating Framework of Management Pro Forma Financial Information in China*. Working Paper. Jiao Tong University, Shanghai. October 2007. https://dx.doi.org/10.2139/ssrn.1021427. Accessed 17 January 2022.

Zhang, Huai, and Liu Zheng. 2011. The valuation impact of reconciling pro forma earnings to GAAP earnings. *Journal of Accounting and Economics* 51: 186-202.

Zhang, Jian. 2019. Learning from the Current Research on Non-GAAP Financial Measures. *CPA Journal* 89(7): 32-37.

Zimmerer, Xaver. 2002. Bilanzen – Sinn oder Unsinn von EBIT/EBITA und EBITDA. *Zeitschrift für das gesamte Kreditwesen* 55: 571.

Annex 4: Analytical framework

A. Bibliographic data (authors, institutions, publication media, years, citations and citations per year): The first category categorises the articles according to bibliographic data and citations (Massaro et al. 2015). The main objective of this category is to analyse the evolution of the literature and its impact on APM research (Massaro et al. 2015). In this way, category 'A. Bibliographic data' allows, for example, an analysis of the existence of the 'superstar' (or 'Matthew') effect, which "appears when a small fraction of researchers or institutions produce the most works and attract a disproportionate number of citations" (Serenko and Dumay 2015; see also Merton 1968; Merton 1988; Rosen 1981). For this purpose, we use the 'equal credit method' (EQM) to determine the productivity of authors and institutions (Serenko and Jiao 2012; Serenko and Dumay 2015). According to this method each author/institution "receives the score of 1/N, where N is the number of authors. For example, for a single-authored paper, each institution … [or author] receives the score of 1.0, two-authored paper – 0.5, three-authored paper – 0.33 and so on" (Serenko and Dumay 2015). The EQM is used for our SLR on APMs because it is relatively simple to use, but at the same time it provides results that compare very well with those generated by more complex methods for quantifying author productivity (Serenko and Jiao 2012). The institutions to which the various authors belong are not limited to academic institutions, as some authors are also practitioners in the field of accounting (e.g., law firms, consulting or accounting firms) (Wagenhofer 2006). In the case that an author belongs to several institutions rather than just one, the assignment is made on the basis of the first mentioned institution. The bibliometric parameters 'citations' (CI) and 'citations per year' (CPY) are based on the approach developed by Dumay (2014a), using *Google Scholar* data (see in detail *Annex 1*). For better comparability, where applicable, the allocation of articles to years is based on the year of publication of the print versions (instead of the online-first publication date).

B. Research focus: The second category is the research focus (e.g., Serenko and Dumay 2015; Dumay 2014a, 2014b; Englund and Gerdin 2014). Analysing the research focus of an article allows to point out specific research areas or topics that might be of interest to other researchers, to identify new research opportunities and to contribute to a better understanding of the scientific dialogue (Massaro et al. 2015). The subcategories of category 'B. Research focus' are based on the classification of research articles on APM reporting according to Hitz (2010) (see *Fig. 1*) and were revised inductively during the coding process. In order to ensure a distinct allocation to the (sub)categories, the coding is based on the primary research focus stated by the authors of the articles (i.e., the first-mentioned research question or hypothesis).

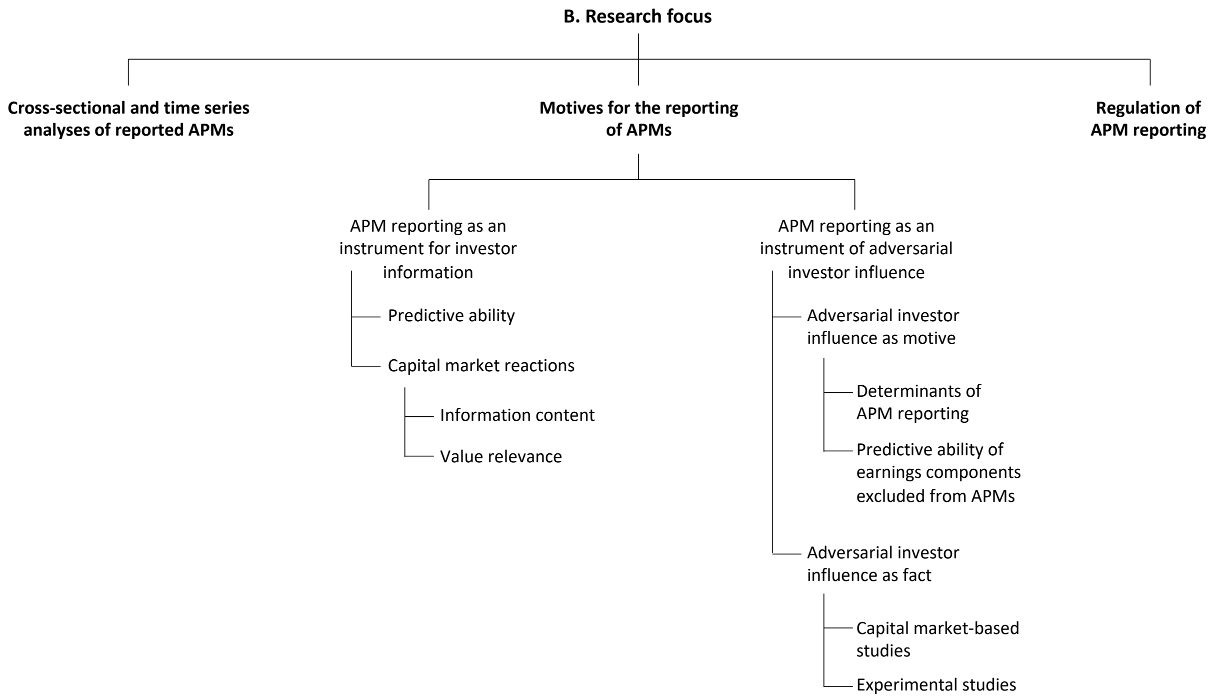

Fig. 1: Categorisation of articles on APM reporting according to Hitz (2010)

C. Research method: The third category classifies the articles according to the research method applied (e.g., Englund and Gerdin 2014; Hoque 2014; Serenko and Dumay 2015). When differentiating research methods, a fundamental distinction can be made between theoretical and empirical accounting research (e.g., Kußmaul et al. 2017; Ernstberger and Werner 2015; Küting et al. 2013; Fülbier and Weller 2008). While for historical reasons the normative-theoretical research approach dominated worldwide, an 'empirical revolution' in international accounting research has been taking place since the beginning of the 1970s, starting in the U.S. (Ernstberger and Werner 2015; Oler et al. 2010; Mattessich 2008). Consequently, a variety of different (normative and empirical) research approaches can be found in international accounting research today. In order to reflect this diversity of research approaches, the subcategories of category 'C. Research method' are based on the methodology according to the *European Accounting Association* (EAA 2019). However, as Broadbent and Guthrie (2008) state, the categorisation depending on the research methods sometimes proves to be problematic, especially when different methods are combined with each other (e.g., mixed-methods designs) in order to guarantee methodological accuracy (reliability). To solve this classification problem, similar to Massaro et al. (2015), the articles were assigned to the subcategories based on the research method stated by the authors in the articles in order to ensure a precise assignment of the articles. However, for 120 articles that do not explicitly mention a specific research method, the assignment to the subcategories was made based on the authors' judgements. These 120 articles were predominantly assigned to the two categories *C7. Non-empirical – theory* (64 articles) and *C8. Non-empirical – opinion* (50 articles). These are mainly articles in professional journals, commentaries and viewpoints, discussion and review articles and contributions to collective works. The different subcategories originally based on EAA's methodology are described in *Tab. 1* below.

C1. Empirical – archival (database or archive)	This category includes articles using sophisticated archival methods to infer relationships in the population (also referred to as 'inductive statistics' or 'inferential statistics') (Smith 2017; Bourier 2013). Archival statistics includes methods that test hypotheses about relations between variables in the population (methods of statistical hypothesis testing) and/or estimate variables in the population (methods of statistical parameter estimation) (Döring and Bortz 2016; Schäfer 2010). Unlike descriptive statistics, these estimates also include the specification of error ranges (confidence intervals) as well as significance tests (Häder 2015). Accordingly, this category particularly includes articles "which involve the testing of a statistical hypothesis to answer the research question(s)" (EAA 2019; similarly Smith 2017). The data sources used in these articles can usually be classified as primary (e.g., manually, hand-collected data through systematic analysis of information disclosed in corporate publications, such as earnings announcements, press releases, quarterly and annual financial statements) or secondary (e.g., data obtained from commercial databases) (Smith 2017).
C2. Empirical – descriptive	This category includes articles that use descriptive methods to organise, summarise and present sample data in order to allow a quick and easy understanding of the distribution of certain characteristics in the sample (Blasius and Baur 2019). While archival methods aim to gain insights into the relationships in the underlying population (point and range estimations) or to perform hypothesis tests, the aim of descriptive methods is simply to describe the sample data (Töpfer 2012). Accordingly, descriptive statistics includes all methods that can be used to summarise and describe sample data. Depending on the type of data obtained, there are different methods for this presentation and description, such as tables, charts or statistical parameters (e.g., proportions, frequencies and measures of location and dispersion) (Schäfer 2010; Döring and Bortz 2016). The findings obtained by means of descriptive statistics relate (only) to the companies included in the sample and therefore generally serve rather as a preliminary stage for hypothesis and theory building (Blasius and Baur 2019; Häder 2015). The data sources used in these articles can usually be classified as primary (e.g., manually, hand-collected data through systematic analysis of information disclosed in corporate publications, such as earnings announcements, press releases, quarterly and annual financial statements).
C3. Empirical – experimental	This category includes articles "which utilise an experimental design to address the research question(s)" (EAA 2019). The defining characteristics of an experimental study design are that the conditions are actively varied (experimental variation), subjects of research are randomly assigned to conditions (at least one experimental and one control group – 'randomisation'), and changes in variables are measured (i.e., pre-post-measurement) (Döring and Bortz 2016).
C4. Empirical – case/field study	This category includes articles "which utilise the case study method or conduct field study research through various methodologies" (EAA 2019). Case and field study research is about examining the role and tasks of accounting in its natural context (Smith 2017). "The term 'case study' usually implies research confined to a single unit of analysis, which might be a single department, company, industry or even country" (Smith 2017). Compared to case studies, in field studies the research subject is less narrow and deals with the social activities of an research subject in its natural environment (Smith 2017).
C5. Empirical – survey	This category includes articles "which utilise a survey methodology to address the research" (EAA 2019). In survey research, researchers ask a (usually randomly selected) sample of people about their background, past experiences, behaviours, etc. (Frankfort-Nachmias et al. 2015). Major methods of survey research include questionnaires, personal interviews and telephone interviews (Frankfort-Nachmias et al. 2015).

C6. Non-empirical – analytical	This category includes articles "which derive their conclusions by an explicit analysis of mathematical models or other concrete specifications of economic relationships" (EAA 2019) such as agency models, game theory or financial modelling.
C7. Non-empirical – theory (objective)	This category includes non-empirical and non-analytical articles "which discuss the purposes of and/or definitions of and/or relationships among concepts and/or apply methodology related to historical perspectives" (EAA 2019). Within this context, a theory is not only understood in the Popperian sense in terms of exploring hypotheses, but rather it represents a set of tentative explanations (Smith 2017) that provide "acceptable answers to interesting questions" (Laudan 1977).
C8. Non-empirical – opinion (subjective)	This category includes articles in which the authors present their subjective opinion, for example on theoretical research aspects (e.g., discussion and review articles) or practical aspects of APM reporting, such as current regulatory initiatives by ESMA or the IASB (e.g., commentaries and viewpoints).
C9. Method not assignable	This category includes:<br>a) articles that use multiple research methods;<br>and<br>b) articles whose research method cannot be easily assigned to the previous mentioned categories for other reasons ('residual category').

Tab. 1: Methodology following the *European Accounting Association* (EAA 2019)

D. Location: The fourth category is the research location (e.g., Massaro et al. 2015). The purpose of this category "is to understand how literature supports the development of a scientific dialogue within specific national contexts that supports the development of knowledge-intensive economies" (Massaro et al. 2015). Therefore, as suggested by Guthrie et al. (2012), the articles were divided into the five regions 'North America' (USA and Canada), 'Australasia' (Australia, New Zealand and Asia), 'United Kingdom' (England, Ireland, Scotland and Wales), 'Europe' (including continental European countries as well as non-continental European countries such as Norway, Finland and Sweden) and 'other', that is the residual category for those countries that cannot be assigned to any of the aforementioned categories (such as Brazil or South Africa). According to Dumay (2014a), in case an article does not mention a clear research location or the article is written about APMs in general, "the article is classified according to the nationality of the first listed author" (similarly Broadbent and Guthrie 2008; Guthrie and Murthy 2009).

E. Accounting regimes: The fifth category is the accounting standards analysed in the articles. The purpose of this categorisation is to understand which accounting regimes have already been researched in depth and for which there may be a need for further research. For this purpose, the articles are assigned to the subcategories depending on the respective accounting regime (e.g., U.S. GAAP or IFRS).

F. Types of APMs: The sixth category is the type of APMs analysed in the articles. The objective of this category is to examine which types of APMs are being addressed in the articles in order to draw conclusions about the most frequently discussed types of APMs. Therefore, according to Hitz (2010), we distinguish between APMs reported by companies (either on a voluntary basis or in mandatory financial reports) on the one hand (*company-reported APMs*) and street earnings provided by analysts on the other hand (*analyst-reported street earnings*). In addition, subcategories were also established for articles that examine both *company-reported APMs and analyst-reported street earnings* as well as for articles that analyse APMs in general and do *not mention a specific type of APM* (e.g., because the articles do not examine empirical data).

G. Data collection: The seventh category is the method of data collection used in the articles analysed. According to Islam et al. (2019), ”[p]rior literature provides evidence of various means for collecting data relating to NGFM”. Also, Marques (2006) emphasises the importance of different methodological approaches in the study of APMs and distinguishes between two fundamental methods of data collection. First, one group of articles examines APMs by selecting commercial databases as proxies for APMs. For example, Bradshaw and Sloan (2002) consider *Thomson Reuters' I/B/E/S* database as a proxy for APMs (similarly Brown and Sivakumar 2003; Cohen et al. 2007; Collins et al. 2009). Second, another group of articles uses both keyword searches for identification and hand-collection of disclosed APMs. For example, Black and Christensen (2009) collect APMs from press releases by searching for keywords to identify APMs (similarly Black et al. 2012; Malone et al. 2016), while Lougee and Marquardt (2004) collect APMs by searching for keywords in the *Lexis-Nexis Academic* database (similarly Islam et al. 2019). Accordingly, the objective of category ‘G. Data collection’ is to examine the data source of APMs analysed in the articles. For this purpose, we distinguish between *manually collected data* (i.e., hand-collected) through systematic analysis of corporate publications, such as earnings announcements, press releases, quarterly and annual (consolidated) financial statements, on the one hand, and *machine-collected data* extracted from databases, on the other hand. In addition, subcategories were also established for articles that use both *manually collected and machine-collected data* as well as for articles that do *not examine APMs empirical data*.

References

Black, Dirk E., and Theodore E. Christensen. 2009. US Managers' Use of 'Pro Forma' Adjustments to Meet Strategic Earnings Targets. *Journal of Business Finance & Accounting* 36: 297-326.

Black, Dirk E., Ervin L. Black, Theodore E. Christensen, and William G. Heninger. 2012. Has the Regulation of Pro Forma Reporting in the US Changed Investors' Perceptions of Pro Forma Earnings Disclosures? *Journal of Business Finance & Accounting* 39: 876-904.

Blasius, Jörg, and Nina Baur. 2019. Multivariate Datenstrukturen. In *Handbuch Methoden der empirischen Sozialforschung*, ed. Nina Baur and Jörg Blasius, 1379-1400. Wiesbaden: Springer VS.

Bourier, Günther. 2013. *Wahrscheinlichkeitsrechnung und schließende Statistik*. Wiesbaden: Springer Gabler.

Bradshaw, Mark T., and Richard G. Sloan. 2002. GAAP versus The Street: An Empirical Assessment of Two Alternative Definitions of Earnings. *Journal of Accounting Research* 40: 41-66.

Broadbent, Jane, and James Guthrie. 2008. Public sector to public services: 20 years of "contextual" accounting research. *Accounting, Auditing & Accountability Journal* 21: 129-169.

Brown, Lawrence D., and Kumar Sivakumar. 2003. Comparing the Value Relevance of Two Operating Income Measures. *Review of Accounting Studies* 8: 561-572.

Cohen, Daniel A., Rebecca N. Hann, and Maria Ogneva. 2007. Another look at GAAP versus the Street: an empirical assessment of measurement error bias. *Review of Accounting Studies* 12: 271-303.

Collins, Daniel W., Oliver Zhen Li, and Hong Xie. 2009. What drives the increased informativeness of earnings announcements over time? *Review of Accounting Studies* 14: 1-30.

Döring, Nicola, and Jürgen Bortz. 2016. *Forschungsmethoden und Evaluation in den Sozial- und Humanwissenschaften*. Berlin et al.: Springer.

Dumay, John. 2014a. 15 years of the Journal of Intellectual Capital and counting. *Journal of Intellectual Capital* 15: 2-37.

Dumay, John. 2014b. Reflections on interdisciplinary accounting research: the state of the art of intellectual capital. *Accounting, Auditing & Accountability Journal* 27: 1257-1264.

EAA. 2019. *Submission & Review Process*. https://eaa2021.virtual.eaacongress.org/r/submission_review_process. Accessed 17 January 2022.

Englund, Hans, and Jonas Gerdin. 2014. Structuration theory in accounting research: Applications and applicability. *Critical Perspectives on Accounting* 25: 162-180.

Ernstberger, Jürgen, and Jörg R. Werner. 2015. Die empirische Revolution in der akademischen Forschung – Folgen für das Verhältnis zwischen Wissenschaft und Praxis in der Rechnungslegung und Wirtschaftsprüfung. *Die Wirtschaftsprüfung* 68: 383-393.

Ernstberger, Jürgen, and Jörg R. Werner. 2015. Die empirische Revolution in der akademischen Forschung – Folgen für das Verhältnis zwischen Wissenschaft und Praxis in der Rechnungslegung und Wirtschaftsprüfung. *Die Wirtschaftsprüfung* 68: 383-393.

Frankfort-Nachmias, Chava, David Nachmias, and Jack DeWaard. 2015. *Research Methods in the Social Sciences*. New York: Worth Publishers.

Fülbier, Rolf Uwe, and Manuel Weller. 2008. Normative Rechnungslegungsforschung im Abseits? Einige wissenschaftstheoretische Anmerkungen. *Journal for General Philosophy of Science* 39: 351-382.

Guthrie, James, and Vijaya Murthy. 2009. Past, present and possible future developments in human capital accounting. *Journal of Human Resource Costing & Accounting* 13: 125-142.

Guthrie, James, Federica Ricceri, and John Dumay. 2012. Reflections and projections: A decade of Intellectual Capital Accounting Research. *The British Accounting Review* 44: 68-82.

Häder, Michael. 2015. *Empirische Sozialforschung*. Wiesbaden: Springer VS.

Hitz, Jörg-Markus. 2010. Information versus adverse Anlegerbeeinflussung: Befund und Implikationen der empirischen Rechnungswesenforschung zur Publizität von Pro-forma-Ergebnisgrößen. *Journal für Betriebswirtschaft* 60: 127-161.

Hoque, Zahirul. 2014. 20 years of studies on the balanced scorecard: Trends, accomplishments, gaps and opportunities for future research. *The British Accounting Review* 46: 33-59.

Islam, Nahid, John Evans, Greg White, and Md Mosharraf Hossain. 2019. Components of CEO Remuneration and Non-GAAP Disclosure. *Australian Accounting Review* 29: 615-630.

Kußmaul, Heinz, Hartmut Bieg, Claus-Peter Weber, Gerd Waschbusch, Alexander Baumeister, Wolfgang Wegener, Michael Olbrich, Alois Paul Knobloch, René Schäfer, and Vassil Tcherveniachki. 2017. Normative theorie- und praxisbezogene Betriebswirtschaftslehre – Methodenpluralismus am Beispiel der Betriebswirtschaftlichen Steuerlehre und der Rechnungslegung. *Der Betrieb* 70: 1337-1343.

Küting, Karlheinz, Heinz Kußmaul, Hartmut Bieg, Claus-Peter Weber, Gerd Waschbusch, Alexander Baumeister, Wolfgang Wegener, Michael Olbrich, and Alois Paul Knobloch. 2013. Saarbrücker Plädoyer für eine normative theorie- und praxisbezogene Betriebswirtschaftslehre. *Der Betrieb* 66: 2097-2099.

Laudan, Larry. 1977. *Progress and its Problems – Towards a Theory of Scientific Growth*. Berkeley et al.: University of California Press.

Lougee, Barbara A., and Carol A. Marquardt. 2004. Earnings Informativeness and Strategic Disclosure: An Empirical Examination of "Pro Forma" Earnings. *Accounting Review* 79: 769-795.

Malone, Lance, Ann Tarca, and Marvin Wee. 2016. IFRS non-GAAP earnings disclosures and fair value measurement. *Accounting & Finance* 56: 59-97.

Marques, Ana. 2006. SEC interventions and the frequency and usefulness of non-GAAP financial measures. *Review of Accounting Studies* 11: 549-574.

Massaro, Maurizio, John Dumay, and Andrea Garlatti. 2015. Public sector knowledge management: a structured literature review. *Journal of Knowledge Management* 19: 530-558.

Mattessich, Richard. 2008. *Two hundred years of accounting research*. London et al.: Routledge.

Merton, Robert K. 1968. The Matthew Effect in Science. *Science* 159: 56-63.

Merton, Robert K. 1988. The Matthew Effect in Science, II: Cumulative Advantage and the Symbolism of Intellectual Property. *Isis* 79: 606-623.

Oler, Derek K., Mitchell J. Oler, and Christopher J. Skousen. 2010. Characterizing Accounting Research. *Accounting Horizons* 24: 635-670.

Rosen, Sherwin. 1981. The Economics of Superstars. *The American Economic Review* 71: 845-858.

Schäfer, Thomas. 2010. *Statistik I*. Wiesbaden: VS Verlag.

Serenko, Alexander, and Changquan Jiao. 2012. Investigating Information Systems Research in Canada. *Canadian Journal of Administrative Sciences* 29: 3-24.

Serenko, Alexander, and John Dumay. 2015. Citation classics published in knowledge management journals. Part I: articles and their characteristics. *Journal of Knowledge Management* 19: 401-431.

Smith, Malcolm. 2017. *Research Methods in Accounting*. Los Angeles et al.: Sage Publications.

Töpfer, Armin. 2012. *Erfolgreich Forschen*. Wiesbaden: Springer Gabler.

Wagenhofer, Alfred. 2006. Management Accounting Research in German-Speaking Countries. *Journal of Management Accounting Research* 18: 1-19.

Annex 5: Supplementary citation analysis

The analysis of the number of articles indicates a growing trend. "However, a growing number of articles published in the field also means a decrease in the value of the average CPY" (Massaro et al. 2015). Since to date there are only few studies on the time lag of citation and diffusion of scientific ideas (e.g., Adams and Clemmons 2013), it is not possible to reliably measure the delay between the publishing of an article and the appearance of citations in other publications (Massaro et al. 2015). For this reason, similar SLRs regularly exclude articles published in the last two years "from all individual CPY scores because there was not sufficient time for the articles to be cited" (Dumay 2014). However, in our sample, a significantly higher proportion of articles with zero citations compared to the other years can only be observed in 2021. Accordingly, the 43 articles published in 2021 were excluded from all individual CPY scores because there is evidence that there was not sufficient time for the articles to be cited. The high proportion of articles in the sample with zero citations of 31.4% across all years is mainly due to the fact that the SLR considers not only frequently cited academic articles, but also less frequently cited professional articles: 73.3% of all 75 articles with zero citations are professional articles. The average CPY amounts to 5.05 and the median is 1.00 (see *Tab. 1*). In terms of distribution, the CPY data show a high level of skewness (4.90) and kurtosis (35.46). When citation frequency is divided into deciles, the maximum concentration (18.3%) is zero CPY which is mainly due to professional articles. However, when adjusting the sample for professional articles, the maximum concentration (12.1%) is still zero CPY. When considering academic articles only, the average is 7.91 CPY and the median is 2.83 CPY. Even after adjusting for professional articles, the sample still shows a high level of skewness (3.95) and kurtosis (23.18). Thus, the distribution of CPYs in both cases (i.e., unadjusted as well as adjusted for professional articles) shows a right-skewed, leptokurtic distribution with high kurtosis. In other words, most of the articles have relatively low CPYs and only a small number of articles shows high CPYs.

**CPY unadjusted** (articles in academic and professional journals)	
Average	5.0526
Median	1.0000
Variance	118.0892
Standard deviation	10.8669
Minimum	0.0000
Maximum	114.0556
Skewness	4.9015
Standard error of skewness	0.1210
Kurtosis	35.4645
Standard error of kurtosis	0.2419

**CPY adjusted** (articles in academic journals only)	
Average	7.9127
Median	2.8333
Variance	172.8034
Standard deviation	13.1455
Minimum	0.0000
Maximum	114.0556
Skewness	3.9508
Standard error of skewness	0.1210
Kurtosis	23.1836
Standard error of kurtosis	0.2419

**CI unadjusted** (articles in academic and professional journals)	
Average	51.5671
Median	7.0000
Variance	24,701.0108
Standard deviation	157.1656
Minimum	0.0000
Maximum	2,053.0000
Skewness	7.9172
Standard error of skewness	0.1210
Kurtosis	85.7583
Standard error of kurtosis	0.2419

**CI adjusted** (articles in academic journals only)	
Average	82.5528
Median	21.0000
Variance	38,315.3192
Standard deviation	195.7430
Minimum	0.0000
Maximum	2,053.0000
Skewness	6.3214
Standard error of skewness	0.1210
Kurtosis	54.3857
Standard error of kurtosis	0.2419

Tab. 1: Descriptive statistics for CI and CPY

References

Adams, James D., and J. Roger Clemmons. 2013. How Rapidly Does Science Leak Out? A Study of the Diffusion of Fundamental Ideas. *Journal of Human Capital* 7: 191-229.

Dumay, John. 2014. 15 years of the Journal of Intellectual Capital and counting. *Journal of Intellectual Capital* 15: 2-37.

Massaro, Maurizio, John Dumay, and Andrea Garlatti. 2015. Public sector knowledge management: a structured literature review. *Journal of Knowledge Management* 19: 530-558.

Annex 6: Supplementary analysis of authors, institutions and journals

1. Authors

The 410 articles included in the sample were written by a total of 628 authors. On average, one author contributed to 1.41 articles (median: 1.00). Of all 628 authors, 19.4% authored or co-authored at least two articles. However, only 44 authors (7.0%) contributed to three or more articles. Of these 44 authors, more than a third (36.4%) were co-authors on at least one of the 'top 25' articles with the highest CPYs. Of all authors, *Theodore E. Christensen* has the highest number of authorships (or co-authorships) with 27 articles, followed by *Ana Marques* (12 articles) and *Ervin L. Black* (11 articles). The equal credit method (EQM)[1] factor amounts to an average of 0.65 and a median of 0.50. An above-average EQM factor is found in 28.3% of all 628 authors. Of these 178 authors with an above-average EQM factor, nine authors have an EQM factor of at least 2.61, which is more than four times above average (see *Fig. 1*). Among these authors, *Theodore E. Christensen* has both the highest EQM factor (9.92) and the highest number of articles (27). Besides *Theodore E. Christensen*, *Ana Marques* also has a much higher EQM factor (7.67) as well as a much higher number of articles (12) compared to the remaining 628 authors. This evidence may indicate the existence of the so-called 'superstar' (or 'Mathew') effect for these two authors (Merton 1968; Merton 1988; Rosen 1981). Interestingly, *Theodore E. Christensen* and *Ana Marques* both show a high variance between the EQM factor and the number of articles, suggesting that the majority of articles published by these authors were co-authorships. Overall, when looking at the authors, it can be concluded that the vast majority of the 628 authors published a relatively low number of articles and only a few authors produced a high number of articles on APMs.

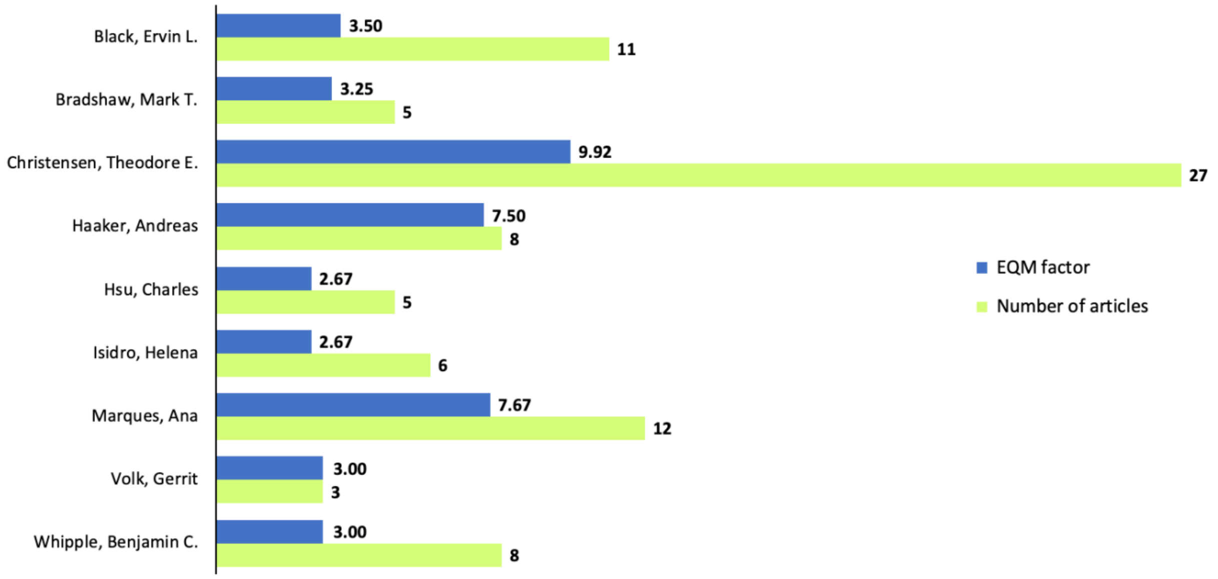

Fig. 1: 'Top 9' authors with the highest EQM factors at least four times above average (0.65)

The analysis regarding the regional origin of the 410 articles shows that the authors mainly belong to institutions in *North America* (58.4%), *Europe* (19.2%), *Australasia* (14.7%) and the *United Kingdom* (2.4%). Furthermore, it can be observed that international collaboration between authors has so far played a rather subordinate role in APM research. On average, authors collaborate across national borders on only one in ten articles (12.0%). Only four articles (1.0%) are the result of collaboration between authors from more than two countries (e.g., Hsu et al.

[1] According to the EQM each author/institution "receives the score of 1/N, where N is the number of authors. For example, for a single-authored paper, each institution … [or author] receives the score of 1.0, two-authored paper – 0.5, three-authored paper – 0.33 and so on" (Serenko and Dumay 2015).

2021; Guillamon-Saorin et al. 2020; Heflin et al. 2015). When looking at the subsample including only academic articles, the proportion of international collaborations amounts to 15.8%. However, international collaborations between authors from more than two countries occurs only take place in 1.8 %. Interestingly, of all the authors associated with institutions in the Australian (and particularly the Asian) region, many chose the U.S. as their research location and also generally chose U.S. GAAP as the accounting regime to be analysed rather than their respective home country and/or accounting regime. This might be due to the high availability of U.S. data as well as the lack of specific regulations for APM reporting in their respective home countries.

2. Institutions

The 410 articles included in the sample were written by authors belonging to a total of 353 institutions. On average, one institution contributed to 1.89 articles (median: 1.00). An above-average number of at least two articles is found in almost two fifths (37.4%) of all 353 institutions. However, only 57 institutions (16.1%) contributed to three or more articles. Of these 57 institutions, almost one third (29.8%) have a participation in one of the 25 articles with highest CPYs. The EQM factor for the sample amounts to an average of 1.09 and a median of 1.00 per institution. An above-average EQM factor is found in roughly a quarter (26.1%) of all 353 institutions. Of these 92 institutions with an above-average EQM factor, 14 institutions (4.0%) have an EQM factor of at least 3.26, which is more than three times above average (see *Fig. 2*). Therefore, the picture is similar to that of the authors: The vast majority of institutions have published a relatively low number of articles and only a small number of institutions have produced a high number of articles on APMs. Of these few institutions, *Brigham Young University* (18 articles), *University of Georgia* (14 articles) and *NOVA University Lisbon* (10 articles) have both the highest number of articles and the highest EQM factor.

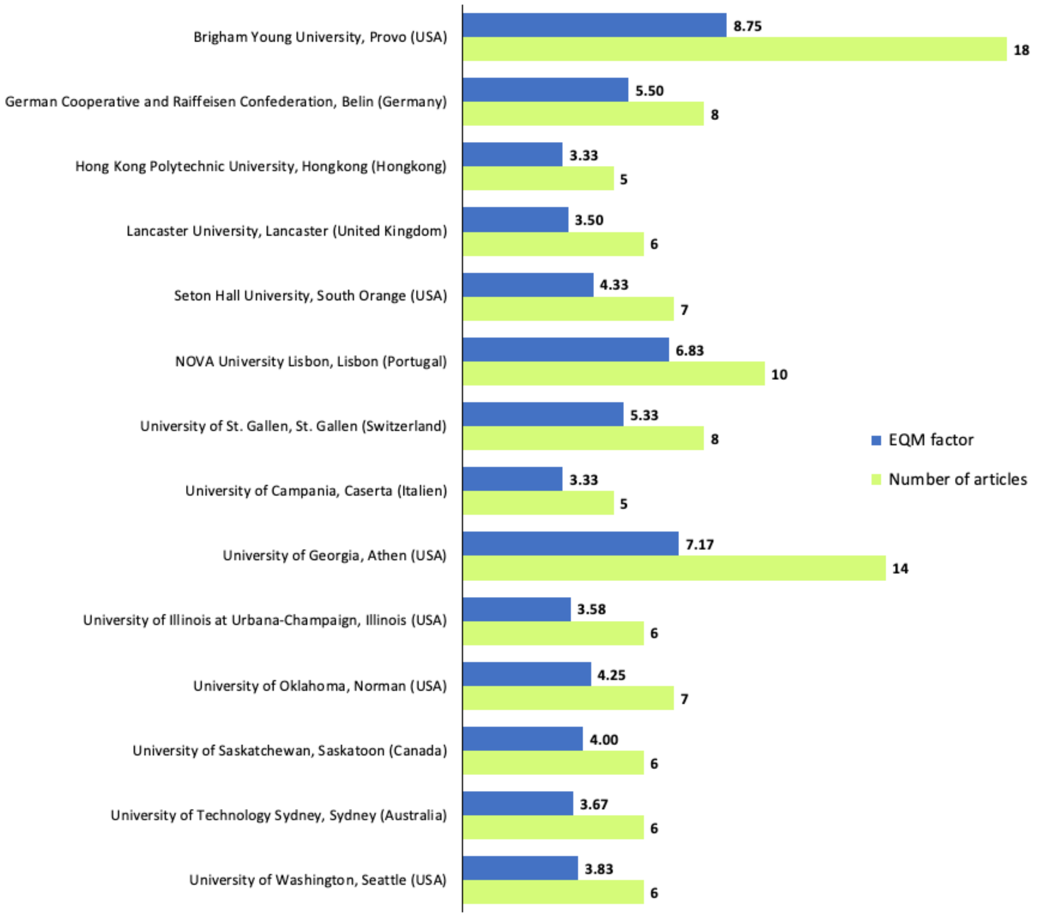

Fig. 2: 'Top 14' institutions with the highest EQM factors at least three times above the average (1.09)

In terms of institutional affiliation, the vast majority of authors (79.5%) are researchers at private or public *universities* (see *Fig. 3*). On average, only one in ten (13.0%) of the authors are members of other *private (i.e., non-governmental) institutions*, such as (industrial) companies (5.7%), auditing firms (2.9%), professional associations and federations (2.5%), law firms (1.2%) or standard setters (0.6%). Of all these private institutions, the *German* Cooperative *and Raiffeisen Confederation* (DGRV) has the highest EQM factor (5.5). The smallest proportion of articles (1.2%) relates to authors at state institutions other than universities, such as the SEC (Bricker and Siegel 2016), Bank of Japan (Shibasaki and Toyokura 2020) or the Beijing National Accounting Institute (Dang 2017), while 6.3% do not specify the author's affiliation.

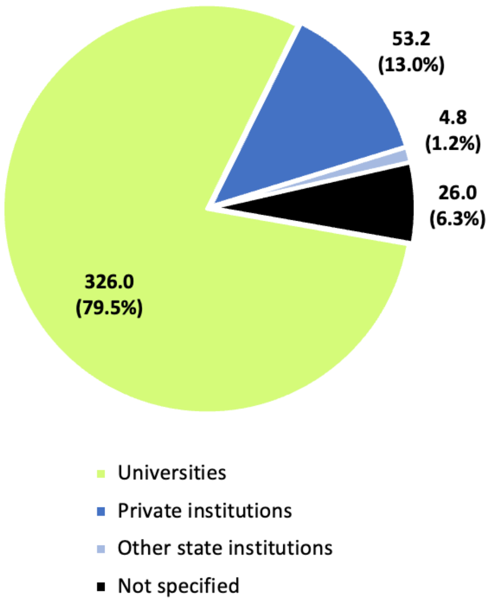

Fig. 3: Institutional affiliation of authors based on EQM factor

3. Journals

The publication media with highest CPYs are solely articles published in academic journals. On average, academic articles have 7.95 CPYs, which is more than twelve times the value compared to professional articles (0.64). The total of 228 academic articles included in the sample were published in 87 different journals. On average, an academic journal published 2.62 articles. The median amounts to 1.00. An above-average number of at least three articles on APMs can be observed in a total of 20 academic journals (see *Fig. 4*). These include academic journals ranked in categories according to the *VHB-JOURQUAL3* ranking, from 'A+' ("excellent and global leading scientific journals in business research") to 'C' ("renowned scientific journals in business research") (VHB 2021). With regard to the 'top 20' academic journals, by far the most articles were published in the *Review of Accounting Studies* (23 articles). The only German-language journal in these 'top 20' journals is *Die Wirtschaftsprüfung* (WPg), which published three articles on APMs and is ranked in category 'C' according to the *VHB-JOURQUAL3* ranking.

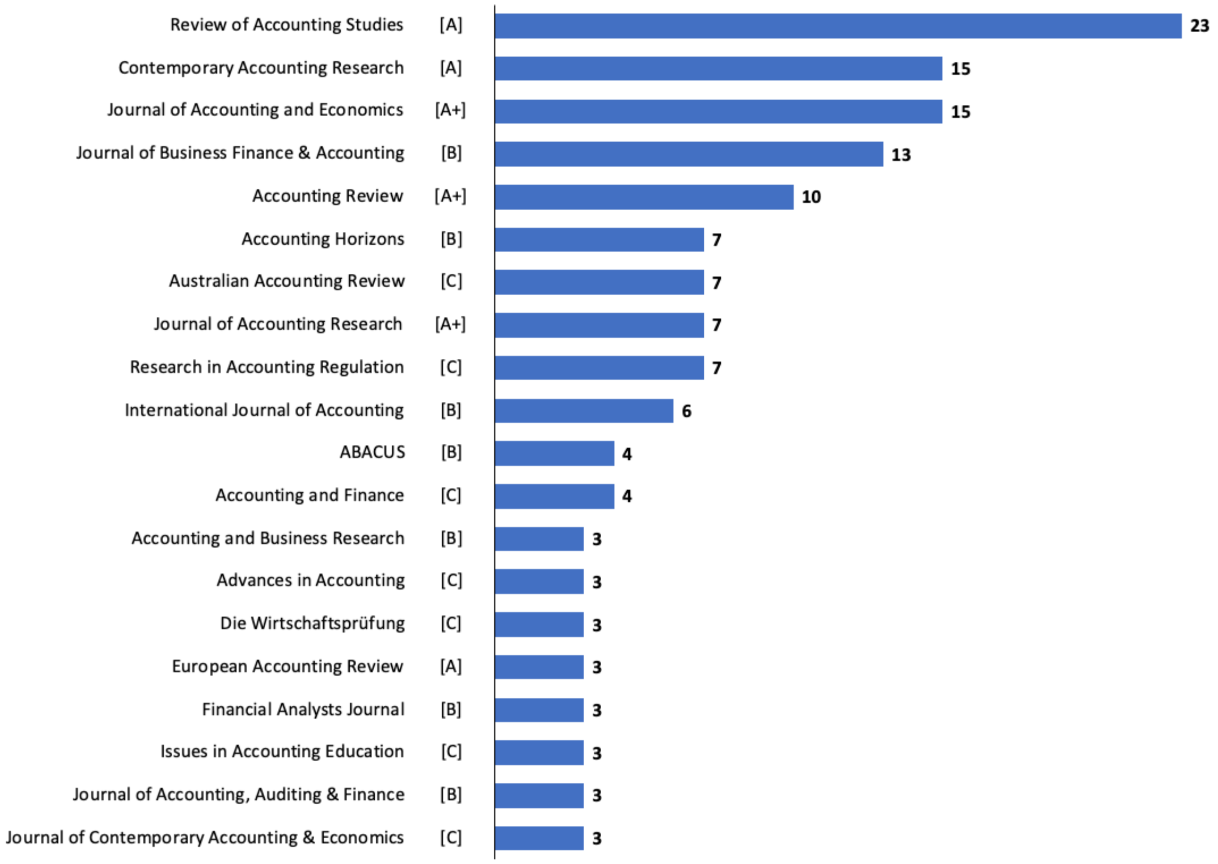

Fig. 4: 'Top 20' academic journals with most articles on APMs above average (2.62)

References

Bricker, Wesley, and Marc Siegel. 2016. Listening to Users on Transition Issues, Non-GAAP Measures, and Disclosures – Remarks from the SEC and FASB. *CPA Journal* 86(7): 28-33.

Dang, Suting. 2017. *Research on Application of Non-GAAP Financial Measures to Overseas Listed Companies*. Conference Paper. 3rd International Conference on Management Science and Innovative Education (MSIE 2017). http://doi.org/10.12783/dtssehs/msie2017/15438. Accessed 17 January 2022.

Guillamon-Saorin, Encarna, Helena Isidro, and Ana Marques. 2020. Reporting of Alternative Performance Measures by European Firms. In *Reporting Non-GAAP Financial Measures*, ed. Nicola Moscariello and Michele Pizzo, 196-223. Newcastle: Cambridge Scholars Publishing.

Heflin, Frank, Charles Hsu, and Qinglu Jin. 2015. Accounting conservatism and Street earnings. *Review of Accounting Studies* 20: 674-709.

Hsu, Charles, Rencheng Wang, and Benjamin C. Whipple. 2021. Non-GAAP earnings and stock price crash risk. *Journal of Accounting and Economics*. https://doi.org/10.1016/j.jacceco.2021.101473.

Merton, Robert K. 1968. The Matthew Effect in Science. *Science* 159: 56-63.

Merton, Robert K. 1988. The Matthew Effect in Science, II: Cumulative Advantage and the Symbolism of Intellectual Property. *Isis* 79: 606-623.

Rosen, Sherwin. 1981. The Economics of Superstars. *The American Economic Review* 71: 845-858.

Serenko, Alexander, and John Dumay. 2015. Citation classics published in knowledge management journals. Part I: articles and their characteristics. *Journal of Knowledge Management* 19: 401-431.

Shibasaki, Yuta, and Chikara Toyokura. 2020. The Disclosure of Non-GAAP Performance Measures and the Adoption of IFRS: Evidence from Japanese Firms' Experience. *Monetary and Economic Studies* 38: 19-54.

VHB. 2021. *VHB-JOURQUAL3*. https://vhbonline.org/en/vhb4you/vhb-jourqual/vhb-jourqual-3. Accessed 17 January 2022.
